# *Chrysomya megacephala* larvae feeding favourably influences manure microbiome, heavy metal stability and greenhouse gas emissions

**DOI:** 10.1111/1751-7915.13253

**Published:** 2018-03-14

**Authors:** Xiaoyun Wang, Wanqiang Wang, Qiao Gao, Xiaoping Wang, Chaoliang Lei, Fen Zhu

**Affiliations:** ^1^ Hubei International Scientific and Technological Cooperation Base of Waste Conversion by Insects Huazhong Agricultural University Wuhan 430070 China; ^2^ Hubei Insect Resources Utilization and Sustainable Pest Management Key Laboratory Huazhong Agricultural University Wuhan 430070 China

## Abstract

*Chrysomya megacephala* is a saprophagous fly whose larvae can compost manure and yield biomass and bio‐fertilizer simultaneously. However, there are concerns for the safety of the composting system, that is risk of diseases spread by way of manure pathogens, residue of harmful metals and emission of greenhouse gases. Microbiota analysis and heavy metal speciation by European Communities Bureau of Reference were evaluated in raw, *C. megacephala*‐composted and natural stacked swine manure to survey pathogenic bacterial changes and mobility of lead and cadmium in manure after *C. megacephala* feeding; the emission rate of CH_4_ and N_2_O from manure during *C. megacephala* composting and natural stacking was also measured. *C. megacephala* composting altered manure microbiota, reduced the risk of pathogenic bacteria and maintained the stability, and microbiota changes might be associated with heavy metal fractions, especially in *Pseudomonas* and *Prevotella*. In addition, *C. megacephala*‐composting significantly reduced the emission rate of CH_4_ and N_2_O in comparing with natural stacking situation and the first two days should be the crucial period for CH_4_ and N_2_O emission measurement for manure treatment by *C. megacephala*. Moreover, OTU26 and Betaproteobacteria were changed after *C. megacephala* composting which might play a role in emission of CH_4_ and N_2_O, respectively.

## Introduction

According to the Food and Agriculture Organization, the production of livestock increases annually at a rapid rate worldwide (http://www.fao.org/home/en/). It was estimated that over 110 billion livestock will be cultivated and slaughtered worldwide by 2050 and the production of pig/swine had reached nearly 1 billion head in 2013 which ranked top fourth among livestock (Ilea, 2009; Zhu and Hiltunen, 2016). It provides the main sources of animal protein in China along with large amounts of manure to be processed (Zheng *et al*., 2014). Therefore, it has long become a problem for developing countries to manage swine manure quickly while combining safety, environmental protection and high efficiency (He *et al*., 2016).

Saprophagous insects could consume swine manure and provide high added‐value products, such as feedstuff, biodiesel and fertilizer (Cickova *et al*., 2015). *Chrysomya megacephala* is a new saprophagous blowfly for transforming kitchen wastes and manure from livestock farms. Larvae of *C. megacephala* are high energy and might be used for providing protein, fat and biodiesel (Li *et al*., 2012). An ideal processing flow was suggested for integration with swine farm; manure transformation of *C. megacephala* would provide win–win profits for both the economy and the environment (Yang and Liu, 2014). However, urgent precautions have been proposed to evaluate potential health risks during the manure management (Committee, 2015), such as the disease‐causing microorganisms, potential heavy metal contamination and greenhouse gas (GHG) emissions.

The pathogenicity of manure has raised concerns for a long time. How bacterial communities, especially for potential pathogenic bacteria, change during composting has always been of interest. Recently, metagenomic 16S rRNA sequencing is a powerful method to explore diversity and structure of microbial communities (Logares *et al*., 2014) which could be applied in diverse ecological and biological niches (Finney *et al*., 2015). In addition, many bacteria are associated with heavy metal absorption, management and greenhouse gas emissions. For example, *Pseudomonas putida* KT2440 could tolerate heavy metals and metalloids (Cánovas *et al*., 2003), and the poultry industry is responsible for methane emission to a certain extent, such as rumen digestion in ruminant (Snelling and Wallace, 2017).

Heavy metals in the manure have generated concern because they cannot be decomposed (Song *et al*., 2014; Lv *et al*., 2016). Direct land application or composted application of heavy metal contaminative manure might lead further pollution to soil. Bioactivity and mobility of heavy metals could be evaluated by sequential extraction methods (Ptistišek *et al*., 2001). The BCR (European Communities Bureau of Reference) method is one of the most commonly used methods for heavy metal speciation (Kede *et al*., 2014; Razek, 2014). Heavy metals are more difficult to be extracted which indicates that they are more settled, less available and have a lower risk to organisms.

Moreover, GHG emissions should be taken into account for a clean manure management system to avoid a secondary pollution. The GHG, carbon dioxide (CO_2_), methane (CH_4_) and nitrous oxide (N_2_O) were key factors for global warming (Solomon *et al*., 2010). According to the FAO livestock holding for only 9% of global CO_2_ emission, however, it represents approximately 35% of CH_4_ and 60% of N_2_O, respectively. Moreover, these two non‐carbon dioxide greenhouse gases (NCGGs) have 23 and 300 times than the global warming potential of CO_2_, respectively (FAO, 2014). The manure management would release CH_4_ and N_2_O during digestion and decomposition, and the production rate could be adjusted by manure storage and handling methods (Paik *et al*., 2016).

Therefore, to evaluate the three possible environment risks during *C. megacephala* manure composting: microbiota changes, the activity of lead (Pb) and cadmium (Cd), the emission rate of methane (CH_4_) and nitrous oxide (N_2_O) during manure handling process was evaluated to assure how *C. megacephala* feeding would influence the pathogenic microbiota of manure; the stability of Pb and Cd and the emission rate of CH_4_ and N_2_O were compared with those of natural stacked manure treatment. These results would promote the utilization of insects to consume organic wastes and provide valuable, available and potential strategies for risk reduction of mobile heavy metals and harmful microbes.

## Results

### *C. megacephala* composting Altered Manure Microbiota

**Figure 1:**
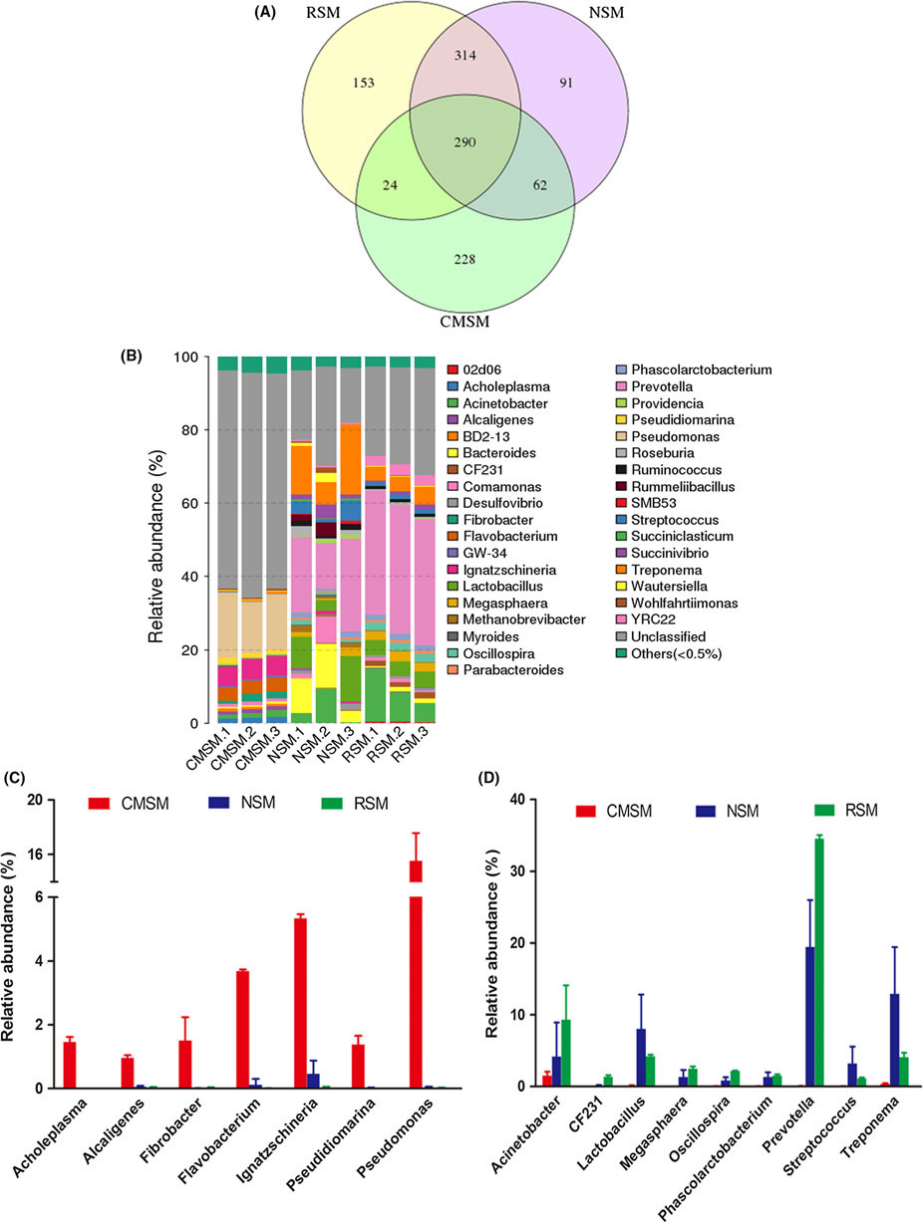
Comparison of swine manure microbiota. A. Venn diagram of shared OTUs of RSM, NSM and CMSM with 97% similarity. B. Microbial composition of RSM, NSM and CMSM at the genus level. The top 37 abundant genera are shown by relative abundance of each bacterial genus within a group. C. The increased genera of CMSM compared with RSM. D. The decreased genera of CMSM compared with RSM. Each bar is the mean with SD from three replicates.

**Figure 2:**
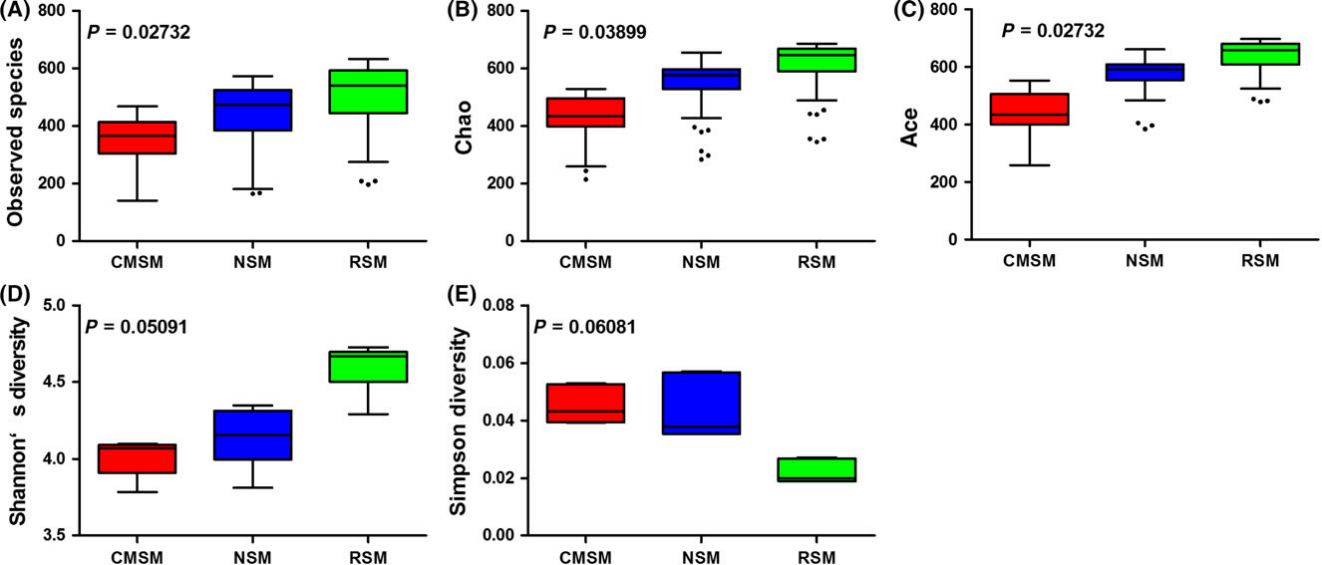
Alpha diversity analysis of RSM, NSM and CMSM. A. Observed species. B. Chao. C. Ace. D. Shannon's diversity. E. Simpson diversity. The five lines of boxplot from bottom to top are the minimum value, the first quartile, median, the third quartile and the maximum value.

**Figure 3:**
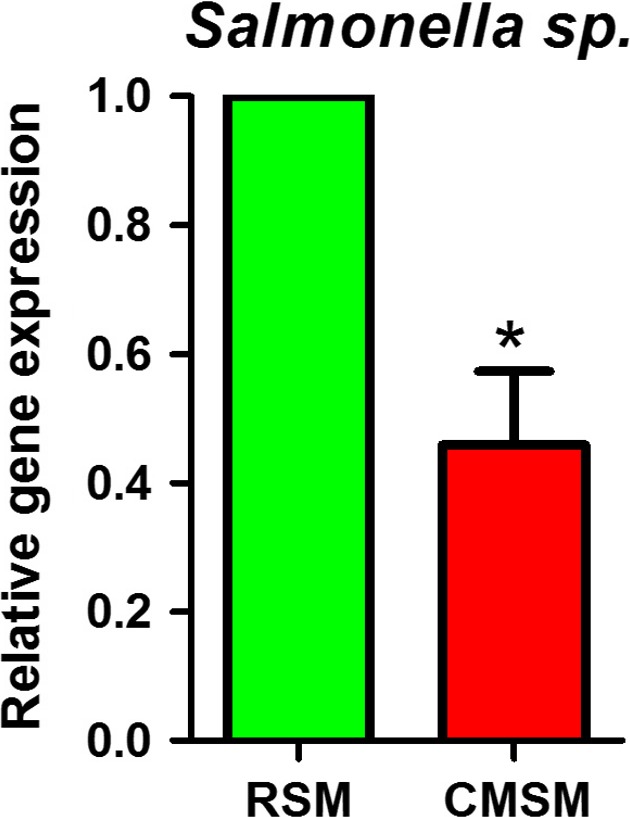
*Salmonella* sp. quantification by PCR. Independent samples test is performed and the asterisk (*) indicates that a significant difference is detected (*P* < 0.05).

On average, approximately 33 000 raw reads per sample were obtained and approximately 32 000 clean reads were obtained with a good read utilization ratio of about 96% per sample. All clean reads were connected to 280 562 tags with a fine connect ratio over 99%. In total, 1162 OTUs were obtained (Table 1, Table S1). First, after *C. megacephala* composting, the count of dominating microorganism changed; 290 OTUs were shared commonly among RSM, NSM and CMSM, and 153, 91 and 228 OTUs were found specifically in RSM, NSM and CMSM, respectively (Fig. 1A, Table S2). In total, 35 known genera were assigned and their relative taxonomic abundance was estimated by a histogram (Fig. 1B). Microbiota of CMSM varied greatly compared with that of RSM according to apparent colour patterns. After deeply digging, the relative abundance of genera *Acholeplasma*,* Alcaligenes*,* Fibrobacter*,* Flavobacterium*,* Ignatzschineria*,* Pseudidiomarina* and *Pseudomonas* increased dramatically in CMSM compared with RSM. Among the increased genera, *Pseudomonas* was the most abundant (Fig. 1C). However, bacterial species of *Pseudomonas* were not well identified in the different abundance analysis at the species level. On the other hand, the relative abundance of genera *Acinetobacter*,* CF231*,* Lactobacillus*,* Megasphaera*,* Oscillospira*,* Phascolarctobacterium*,* Prevotella*,* Streptococcus* and *Treponema* decreased sharply. The relative abundance of *Prevotella* in the RSM decreased from approximately 35% down close to zero (Fig. 1D). Second, after *C. megacephala* composting, species composition of manure altered. Alpha diversity in bacterial communities of RSM, NSM and CMSM was calculated by five methods (Fig. 2). Observed species (*P* = 0.02732), Chao (*P* = 0.03899) and ace (*P* = 0.02732) indicated that a significant difference was observed among the three groups. However, Shannon's diversity (*P* = 0.05091) and Simpson diversity (*P* = 0.06081) indicated that no significant difference was observed. Generally, *C. megacephala* played a role in bacterial community changes during swine manure composting. Beta diversity distance showed similar results, which are listed in Table S3. Common *Salmonella* sp. are faecal indicator bacteria (Mantha *et al*., 2017) and were significantly reduced after *C. megacephala‐*treated swine manure (Fig. 3).

**Table 1:** Sample information and number of sequences obtained

Sample name	Raw reads	Clean reads	Utilization ratio of reads (%)	Tags	Connect ratio (%)	OTUs
RSM.1	33 831	31 558	93.28	31 332	99.28	614
RSM.2	33 718	31 494	93.4	31 278	99.31	633
RSM.3	34 250	31 614	92.3	31 422	99.39	616
NSM.1	33 574	31 454	93.69	31 176	99.12	546
NSM.2	33 254	31 702	95.33	31 459	99.23	573
NSM.3	33 696	31 461	93.37	31 208	99.20	537
CMSM.1	32 455	31 314	96.48	31 083	99.26	388
CMSM.2	32 278	30 993	96.02	30 786	99.33	444
CMSM.3	32 206	31 057	96.43	30 818	99.23	470

### *C. megacephala* composting maintained heavy metal stability

**Figure 4:**
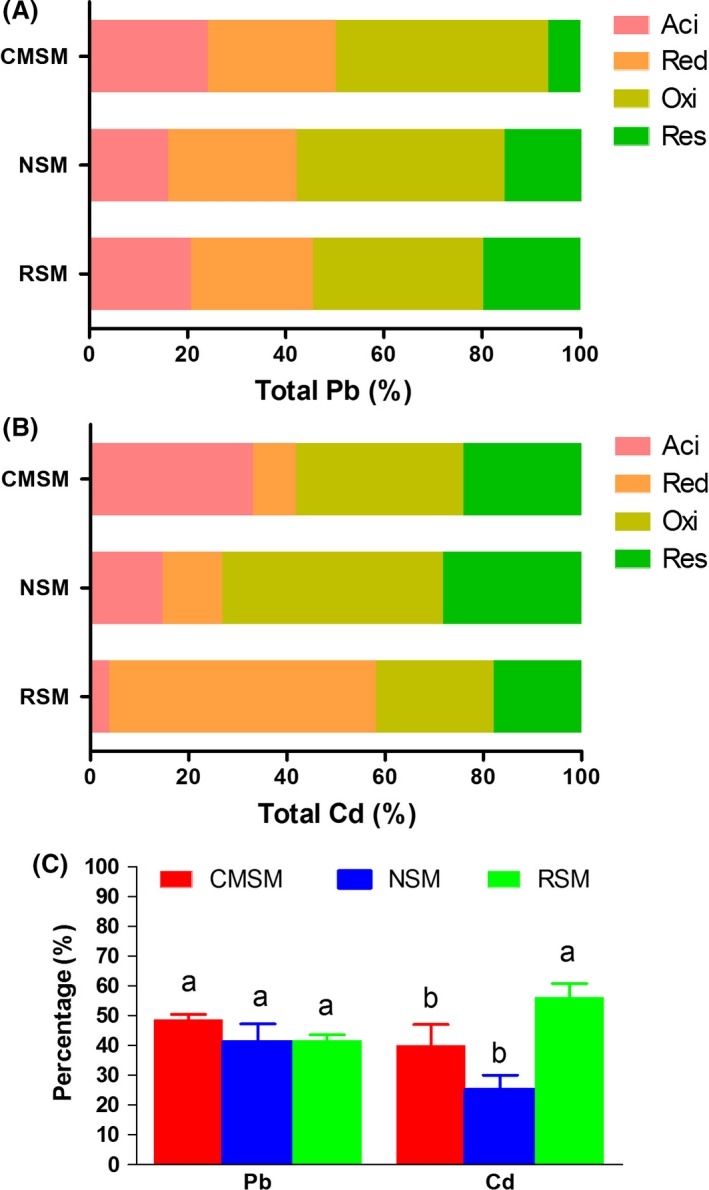
Heavy metal speciation comparison of swine manure. A. Speciation of Pb in RSM, NSM and CMSM. Each bar indicates an average value. Four steps were conducted for the sequential extraction of acid‐soluble fraction‐bound carbonates (Aci), reducible fraction‐bound Fe and Mn oxides (Red), oxidizable fraction‐bound organic matter and sulphides (Oxi) and residual fraction strongly associated with the crystalline structures of the minerals (Res), respectively. B. Speciation of Cd in RSM, NSM and CMSM. Each bar indicates an average value. Abbreviation of Cd speciation is the same as Pb. C. Comparisons of the mobility factor of Pb and Cd in RSM, NSM and CMSM. Each bar indicates a value (mean with SD). The mobility factor (MF) was calculated as the percentage of heavy metal in Aci+Red fractions to the total contents to evaluate the potential mobility of the heavy metals. The LSD test was performed, and different letters indicate that a significant difference is detected (*P* < 0.05).

Fractions of metals did not vary significantly after *C. megacephala* composting. Pb and Cd speciation of RSM, NSM and CMSM was shown as four fractions, and the mobility factor was calculated accordingly. Speciation of Cd changed more than that of Pb (Fig. 4A and B). Although no significant difference was detected in the mobility of Pb, the active fractions of Pb increased to a certain extent, while the mobility of Cd decreased during *C. megacephala* composting and natural stack conditions (Fig. 4C).

**Figure 5:**
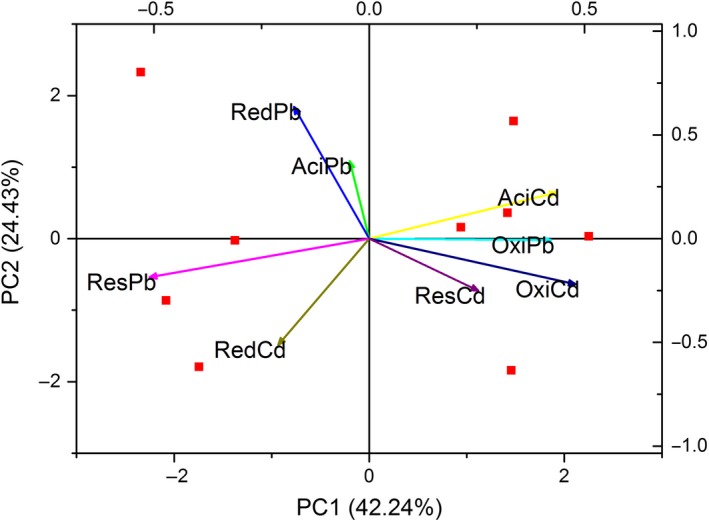
Principal component analysis of swine manure in heavy metal speciation.

**Figure 6:**
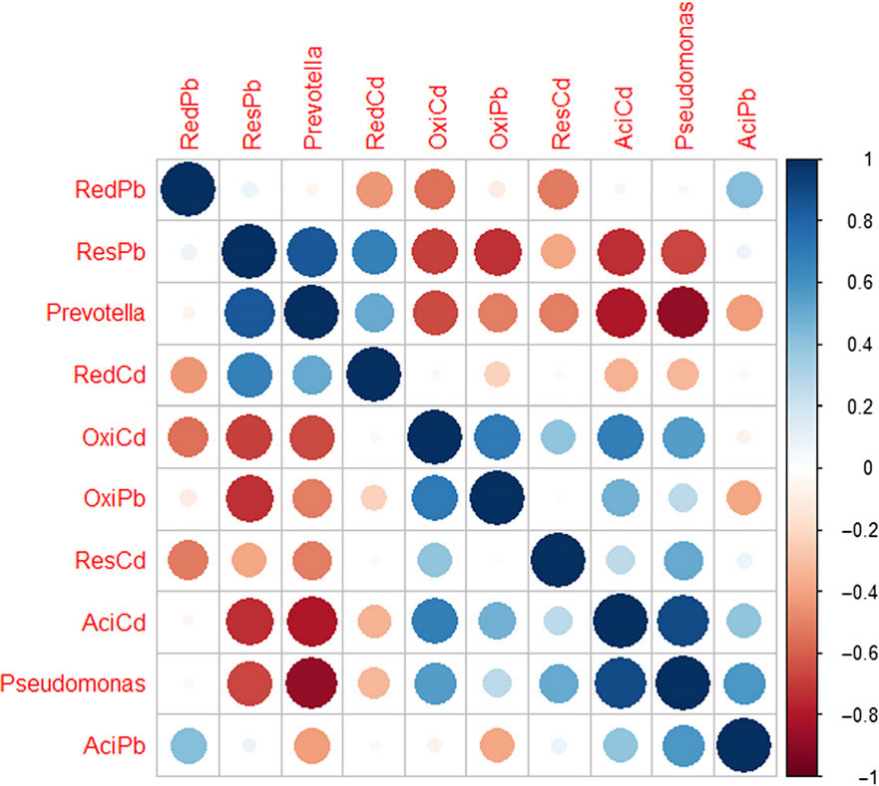
Correlation coefficient matrix of genera *Prevotella*,* Pseudomonas* and heavy metal speciation.

A PCA plot showed that Pb and Cd speciation of NSM and CMSM was similar. Pb and Cd fractions of swine manure were divided into different groups. Cd made a greater contribution than Pb did in the first two axes. *C. megacephala* composting affected the ResCd and OxiCd the most (Fig. 5). The correlation analysis of *Pseudomonas*,* Prevotella* and heavy metal speciation was viewed by a matrix graph (Fig. 6). A significant correlation was detected in *Pseudomonas* and ResPb (*r* = −0.74478, *P* = 0.02132), *Pseudomonas* and AciCd (*r *= 0.87815, *P* = 0.00184), *Pseudomonas* and OxiCd (*r* = 0.77825, *P* = 0.01351), *Prevotella* and ResPb (*r* = 0.81667, *P* = 0.00722), *Prevotella* and AciCd (r = −0.69457, *P* = 0.03786), and *Prevotella* and OxiCd (*r* = −0.66667, *P* = 0.04987). It seems that the correlations between heavy metal speciation and *Pseudomonas* and *Prevotella* were different based on opposite colour view. Moreover, heavy metal fractions ResPb, AciCd and OxiCd are consistent with the results of Fig. 6 and Fig. S2. The genus *Pseudomonas* was not refined to the species level in this study, while the genus *Prevotella* refined to *Prevotella copri* and *Prevotella stercorea*. The correlation matrix graph of heavy metal speciation and *Prevotella copri*/*Prevotella stercorea* are shown in Table S4, 5. A significant correlation was detected in *Prevotella copri* and ResPb (*r* = 0.88333, *P* = 0.00159), *Prevotella copri* and AciCd (*r* = −0.81172, *P* = 0.00789), *Prevotella stercorea* and ResPb (*r* = 0.93234, *P* = 2.48E‐04), *Prevotella stercorea* and AciCd (*r* = −0.92774, *P* = 3.11E‐04), and *Prevotella stercorea* and OxiCd (*r* = −0.74587, *P* = 0.02103). Except for *Prevotella copri* and *Prevotella stercorea*, different abundance of other bacterial species is shown in Table S6.

### *C. megacephala* composting facilitated GHG emission management

**Figure 7:**
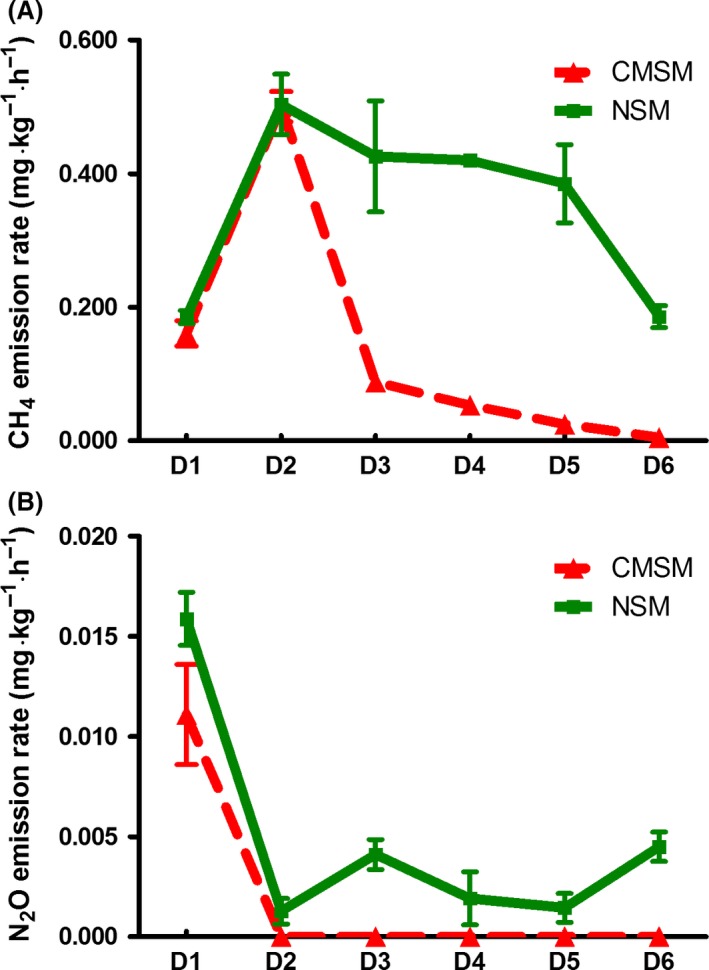
Emission rate of greenhouse gas from swine manure during *C. megacephala* and natural composting. A. Emission rate of CH_4_; B. Emission rate of N_2_O.

The emission of CH_4_ from CMSM and NSM is shown in Fig. 7A. The first 2 days of CH_4_ emissions from CMSM and NSM had a remarkable rise. After that, the CH_4_ emission rate showed different decreasing trends. The emission of CH_4_ from CMSM on day (D) 3 dropped considerably, then kept a decreasing trend and almost reached zero on D6. However, the emission of CH_4_ from NSM kept on a relative high level with a slightly decreasing trend. Moreover, on D6 of NSM, it still had an almost equal rate on D1 with the original manure. The N_2_O emission of CMSM and NSM is shown in Fig. 7B. The emission of N_2_O from both NSM and CMSM happened on D1. Moreover, the emission rate of N_2_O from NSM was almost two times than that of CMSM. On later days, the emission rate from CMSM was undetected indicating that the N_2_O emissions might not happen. Meanwhile, the emission from NSM dropped down to almost zero on D2 and then fluctuated at a relative low rate. In summary, the emission rate of CH_4_ was much higher than that of N_2_O and the emission of CH_4_ and N_2_O was lower in CMSM than in NSM. The first 2 days were the emission peak. Taking the clean production of the *C. megacephala* composting system into consideration, the first 2 days of composting were a critical period to take steps.

### GHG‐Associated manure microbiota

**Figure 8:**
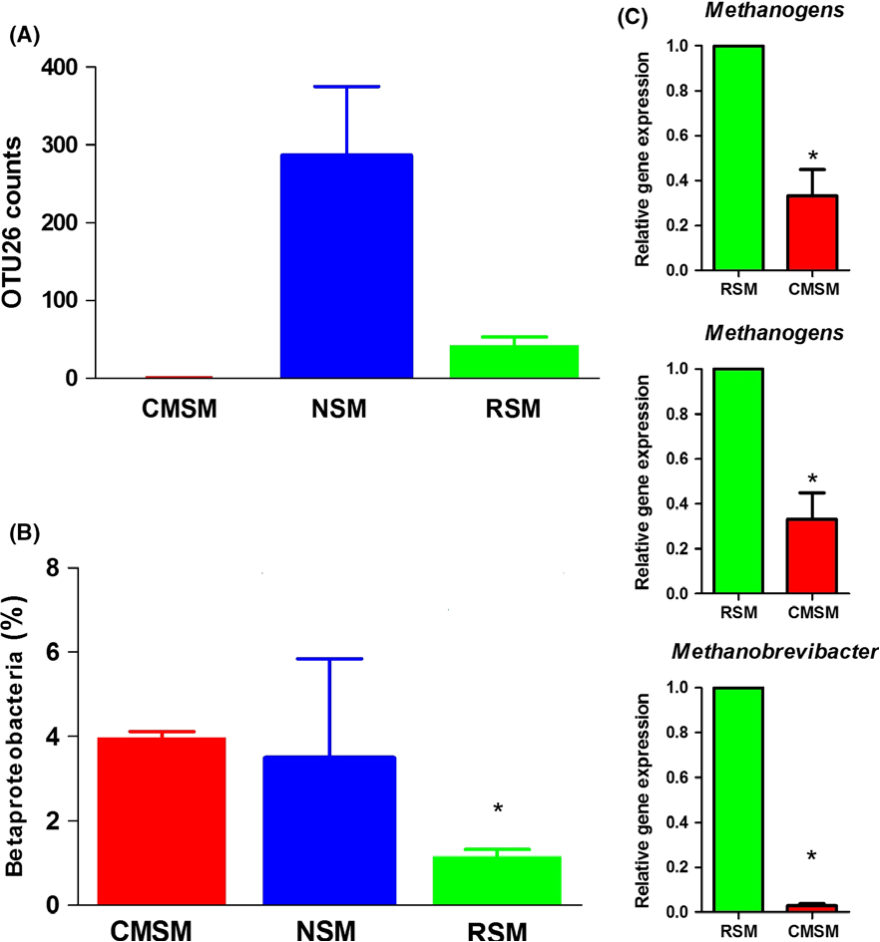
Changes in CH_4_ and N_2_O‐associated bacteria. A**.** The counts of OTU26 in RSM, NSM and CMSM. B. The relative abundance of Betaproteobacteria in RSM, NSM and CMSM. C**.** Methanogenic bacterial quantification by relative gene expression. Independent samples test is performed and the asterisk (*) indicates that a significant difference is detected (*P* < 0.05).

CH_4_ and N_2_O‐associated bacterial changes before and after *C. megacephala* composting are shown in Fig. 8. OTU26, OTU984 and OTU624 were clustered as methanobacteria which belong to Archaea and contribute to metabolize CH_4_. OTU26 counts the most times, and nearly none were detected after *C. megacephala* composting (Fig. 8A). Nitrification and denitrification are two major processes to contribute to N_2_O emissions which take effect under aerobic and anaerobic conditions, respectively (Rogers and Whitman, 1994). Ammonia oxidation is a critical process of nitrification, and ammonia oxidizing bacteria (AOB) are mostly from class Betaproteobacteria (Arp and Stein, 2003). The relative abundance of Betaproteobacteria was significantly increased after *C. megacephala* composting (Fig. 8B). *Methanogens*,* Methanomassiliicoccaceae* and *Methanobrevibacter* are three methanogenic bacteria (Duarte *et al*., 2017), and all of them were detected to have a significantly lower expression in CMSM than RSM which indicated that *C. megacephala* composting might reduce the methane emission by inhibiting methanogenic bacteria (Fig. 8C).

### Sequence deposit

The raw pyrosequencing and Illumina read data for all samples have been deposited to the Sequence Read Archive (SRA) of the National Center for Biotechnology Information (NCBI) under accession number SRP072077.

## Discussion

After *C. megacephala* composting, the bacterial composition of swine manure changed which might have a double effect on manure bacterial‐related disease. The diversity of CMSM was markedly lowered compared with that of RSM. Accordingly, on one side, 19 bacterial species of CMSM were found to be lower than that of RSM. Among them, 10 species reached a significant level. Most of the decreased species were gut microbes. Using genus *Prevotella* as an example, *Prevotella copri* and *Prevotella stercorea* were identified to decrease to near zero in CMSM compared to RSM (Fig. S1). *Prevotella copri* and *Prevotella stercorea* are animal gut microbes that were first isolated from human faeces (Hayashi *et al*., 2007). *Prevotella copri* and *Prevotella stercorea* were related to rheumatoid arthritis and carcinoma in adenoma, respectively (Scher *et al*., 2013; Moreno, 2015; Kasai *et al*., 2016). On the other side, 15 species were identified to be more abundant in CMSM than in RSM. Among them, only *Acholeplasma laidlawii*,* Alcaligenes faecalis* and *Flavobacterium gelidilacus* reached a significant level. *Acholeplasma laidlawii* is a unique mycoplasma species that was widely distributed and related to environmental adaptation, ‘parasite‐host’ system and virulence realization (Lazarev *et al*., 2011; Chernov *et al*., 2012). *Alcaligenes faecalis* even cause peritonitis occasionally (Kahveci *et al*., 2011). The identified *Alcaligenes faecalis* strain MOR02 from *Galleria mellonella* larva is dangerous and pathogenic to larva as a result of toxic protein secretion and nematode cooperation (Quiroz‐Castañeda *et al*., 2015). Increased *Alcaligenes faecalis* in CMSM might originate from excretion or from dead bodies of *C. megacephala*. Notably, *C. megacephala* were well grown in swine manure*. *It could be speculated that *C. megacephala* is not sensitive or that it possesses corresponding strategies to fight back. *Flavobacterium gelidilacus* was isolated from microbial mats in Antarctic lakes and might have a similar function in consuming the high molecular mass fraction of dissolved organic matter (Stefanie *et al*., 2003). Moreover, *Flavobacterium gelidilacus* was not detected in RSM; *Flavobacterium gelidilacus* in NSM was also identified to be much less than in CMSM. Therefore, the feeding and movement of *C. megacephala* larvae might contribute in this respect, and role of *Flavobacterium gelidilacus* is worth further confirmation. *Salmonella sp*. was significantly reduced after *C. megacephala* larvae consuming. *Salmonella sp*. are faecal indicators in wastewater (Mantha *et al*., 2017), which suggested that *C. megacephala* composting might reduce the risk in *Salmonella* Enteritidis, a standout foodborne disease worldwide (Borges *et al*., 2017).

Based on the correlation analysis of *Pseudomonas*,* Prevotella* and heavy metal speciation (Fig. 6), it seems that the correlations between heavy metal speciation and *Pseudomonas* and *Prevotella* were different. Moreover, bacteria related to heavy metal speciation during *C. megacephala* composting were identified for potential application. Bacteria of genus *Pseudomonas* were of interest because of their high resistance to heavy metals and other toxicants (Pardo *et al*., 2003). *Pseudomonas fluorescens, Pseudomonas putida* and *Pseudomonas stutzeri* were reported to have a high resistance to Pb and Zn (Ceylan and Aysel, 2012). *Pseudomonas fluorescens* strain ZY2 isolated from swine wastewater had a cross‐resistance to Pb and some antibiotics (Zhou *et al*., 2015). *Pseudomonas putida* combined with humic acid had a higher Cd sorption; Cd was preferentially bonded to the bacteria in the ternary clay mineral‐humic acid‐bacteria higher affinity composite (Du *et al*., 2016). Bacteria of *Pseudomonas* also performed well in dealing with other heavy metals. For example, *Pseudomonas sp*. JF122, *Pseudomonas gessardii* strain LZ‐E and *Pseudomonas taiwanensis* would be a potential applicant for chromium (Cr) remediation (Huang *et al*., 2016; Majumder *et al*., 2016; Zhou and Chen, 2016). *Prevotella* is a genus of Gram‐negative bacteria, which is closely related to various infections as introduced above (Könönen *et al*., 1998). No studies have shown that *Prevotella* contributes to heavy metal speciation changes during composting. It might be possible that the decrease in *Prevotella* relative abundance might be derived from the increase in other bacteria, for example *Pseudomonas*. There was no definite conclusion on the associations between bacteria and heavy metal speciation.

People are concerned about the safety of manure bio‐fertilizer because of possible metal accumulation and transferring. For example, short‐term fertilization with pig manure‐based compost increased the content of Pb and Cd in soil (Tian *et al*., 2015). Moreover, the Cd content in plants was positively correlated with Cd‐contaminated soils, indicating that risks would occur with Cd transfer (Nookabkaew *et al*., 2016). Heavy metals which are extracted difficultly from substances indicate that they are unlikely to be transferred into environment and might reduce toxic risks in entering organisms. The more difficult to be extracted, more difficult to be transferred and less mobile and lower risky for a heavy metal. According to Fig. 4, Cd in CMSM seems to be easier to migrate from swine manure into plants than Pb because the percentage of ResCd was lower than that of Pb. Similar results were found in the contaminated soils that Cd was more labile than Pb (Ptistišek *et al*., 2001; Kede *et al*., 2014). However, we were pleased to see that *C*. *megacephala* maintained the stability of containing metals in manure which are acceptable in environment. What we have to control when using *C*. *megacephala* to transform manure is the original content of heavy metals because some manure might contain a higher value of heavy metals than permitted (Zhang *et al*., 2012).

We concluded that the first 2 days would be the crucial processing period for GHG emission management which might result from the decrease in methanogenic bacteria. Furthermore, another merit of *C. megacephala* swine manure composting is that it lasted for a short time. In the long term of swine manure composting, the emission peak of CH_4_ occurred immediately after swine manure was piled up, and the N_2_O occurred around the middle stage of the composting period and thus had a long gas control term as well (Fukumoto *et al*., 2003). Centralized control measures of *C. megacephala* swine manure composting could reduce the maintenance of device and energy consumption. The full‐scale bio‐filter with rock wool mixture that used in livestock manure composting might also be used in the construction of *C. megacephala* swine manure composting system (Yasuda *et al*., 2009). Another merit of *C. megacephala* swine manure composting is that it uses relatively less area of soil with shelf production system (Yang and Liu, 2014). Earthworms could also reduce the GHS emission during composting (Lim *et al*., 2016). However, in China, earthworms composting has a relatively longer period and larger land use than *C*. *megacephala* composting because earthworms composting is a continuous system and *C*. *megacephala* composting is batch model (Zhang *et al*., 2013).

Pilot‐scale biodegradation of swine manure via *C. megacephala* larvae for biodiesel production has been reported (Yang and Liu, 2014). The manure processing system in this study also used 6 kg as a treatment tank with more eggs to ensure that the manure was fully transformed. We also used manure from the same farm contained no sawdust with a moisture around 70%. The system was downsized in this study; however, the measured parameters should have reference values as reaction material proportion was not changed. Therefore, relative environmental parameters can help develop environmental protection measures in this pilot scale, especially in microbiological safety, heavy metal and greenhouse gas emission control.

In conclusion, *C. megacephala* composting altered manure microbiota, reduced the risk of pathogenic bacteria and maintained the stability, and microbiota changes might be associated with heavy metal fractions, especially in *Pseudomonas* and *Prevotella*. In addition, during *C. megacephala* composting of manure significantly reduced the emission rate of CH_4_ and N_2_O in comparing with natural stacking situation and the first 2 days should be the crucial period for CH_4_ and N_2_O emission measurement during *C. megacephala* composting. Moreover, OTU26 and Betaproteobacteria were changed after *C. megacephala* composting which might play a role in emission of CH_4_ and N_2_O, respectively.

## Experimental procedures

### Experiment design and swine manure sampling

*Chrysomya megacephala* was provided by Hubei International Cooperation Base for Waste Conversion by Insects, and swine manure was taken from the swine breeding farm of the Huazhong Agricultural University (HZAU). Adults of *C. megacephala* were reared in mesh cages (35 × 35 × 35 cm) with water and sugar, and the cages were kept in a rearing room at 25 ± 3°C under a 13:11‐h light: dark photoperiod. Eggs were first collected in a swine manure gauze bag by putting into cages for 4 h, and then, eggs were separated from the gauze. The manure was well mixed by electric blender as raw swine manure (RSM) and then applied for different treatments: *C. megacephala*‐transformed manure (CMSM) and natural stacked manure (NSM). On average, 6 kg of manure was applied for each treatment in plastic tanks. For CMSM treatment, eggs were loaded on manure in the proportion of 1.5 g eggs per kilogram manure. Before and after the conversion process, RSM, CMSM and NSM were sampled in 1.5‐ml sterile centrifuge tube and stored at −80°C for DNA extraction. Five tubes of different manure types were applied for DNA extraction and combined as one sample to ensure the samples to be representative. RSM, CMSM and NSM manure samples were also sampled, oven dried at 70°C and sieved for heavy metal speciation. During the conversion process, static closed chambers were also applied for measuring the emission rate of CH_4_ and N_2_O. Each experiment was conducted three times.

### DNA extraction, PCR, pyrosequencing and bioinformatics analysis

Genomic DNA of manure samples was extracted by a TIANamp Stool DNA Kit (TIANGEN Biotech Beijing, China: DP328). After concentration and integrity testing, the genomic DNA for individual sample was normalized to 30 ng per PCR. The sequencing for bacterial variable V4 regions of the 16S rDNA gene was performed by BGI Tech (BGI Tech Solutions Co., Ltd., Wuhan, China) on the Illumina MiSeq platform with the 515f/806r primer set (515f: 5′‐GTG CCA GCM GCC GCG GTA A‐3′, 806r: 5′‐XXX XXX GGA CTA CHV GGG TWT CTA AT‐3′). Three replicates were conducted. Raw sequences of all samples were processed accordingly to obtain clean data by filtering out reads with sequencing adapters, N base, poly base and low quality (Fadrosh *et al*., 2014). Clean data of swine manure samples were applied for tag generation by FLASH and operational taxonomic units (OTUs) cluster analysis by USEARCH (Magoč and Salzberg, 2011; Edgar, 2013) to survey bacterial communities in natural composting and *C. megacephala* composting conditions. Then, OTUs classification, alignment of the representative sequence of each OTU, chimera removal, taxonomic assignment and alpha and beta diversity analyses were performed with QIIME (macQIIME 1.7) (Caporaso *et al*., 2010).

### Quantitative PCR validation

SYBR^®^ Premix Ex Taq™ II (Tli RNaseH Plus) was used for quantitative PCR (Cat#: RR820A; Takara, Beijing, China). The reaction system was prepared as SYBR 5 μl; H_2_O 3.6 μl; primer F/R 0.2 μl; DNA template 1 μl (40 ng μl^−1^). The PCR was performed on QuantStudio 6 Flex Real‐Time PCR System (Life Technologies, Waltham, USA) following 40 cycles of 95°C 30 s; 95°C 5 s; 60°C 30 s. Primers of *Methanogens*,* Methanomassiliicoccaceae*,* Methanobrevibacter*,* Salmonella* sp. and *16S* are listed in Table S7. The expression level was calculated by $2^{-\Delta \Delta C_{T}}$ method with 16S as a reference gene.

### Speciation and mobility assessment of heavy metal

The BCR method was applied to the swine manure (Cuong and Obbard, 2006). Four steps were conducted for the sequential extraction of acid‐soluble fraction‐bound carbonates (Aci), reducible fraction‐bound Fe and Mn oxides (Red), oxidizable fraction‐bound organic matter and sulphides (Oxi) and residual fraction strongly associated with the crystalline structures of the minerals (Res), respectively (Ptistišek *et al*., 2001; Cuong and Obbard, 2006). The content of lead (Pb) and cadmium (Cd) was measured with Agilent Technologies 240FS AA (USA). Moreover, the mobility factor (MF) was calculated as the percentage of heavy metal in Aci+Red fractions to the total contents to evaluate the potential mobility of the heavy metals (Lv *et al*., 2016). A permutation test of BCR speciation was conducted by SAS (USA). Principal component analysis (PCA) and correlation analysis were conducted with Origin 9.0. Figures were partly selected from R‐based reports of BGI Tech and partly made by GraphPad Prism 5.0 (GraphPad Software, La Jolla, USA).

### GHG emission rate

The content of CH_4_ and N_2_O was measured with the gas chromatography (GC, Shimadzu GC‐14B) according to the relative peak area of the standard gas (Li *et al*., 2013). The fluxes of CH_4_ and N_2_O were calculated as described by the following equation:

$$F=\rho \times V\times dC/dt\times 273÷\left(273+T\right)$$

where *F* denotes the emission of CH_4_ and N_2_O as mg kg^−1 ^h^−1^; ρ denotes the CH_4_ (0.717 kg m^−3^) or N_2_O (1.978 kg m^−3^) density; dC∕dt indicates the accumulation rate of CH_4_ and N_2_O; V is the volume of the chamber on top; T is the number of mean temperature inside the chamber. The density of the standard gas used in this study for CH_4_ and N_2_O was 1.79 and 0.353 mg l^−1^, respectively. The preceding calculation process was referred from Zhang *et al*. (Zhang *et al*., 2015).

## Conflict of interests

The authors declare that they have no conflict of interests.

## Supporting information


**Fig. S1** Relative abundance of *Prevotella copri* and *Prevotella stercorea* in RSM, NSM and CMSM.

**Fig. S2** Correlation coefficient matrix of heavy metal speciation

**Table S1** Taxonomy of OTUs.

**Table S2** Detailed OTUs of RSM, NSM and CMSM in Venn diagram.

**Table S3** Beta diversity distance of RSM, NSM and CMSM.

**Table S4.** Correlation matrix graph of heavy metal speciation and *Prevotella copri* (Pcop).

**Table S5.** Correlation matrix graph of heavy metal speciation and *Prevotella stercorea* (Pste).

**Table S6.** Different abundance of bacteria species in RSM, NSM and CMSM.

**Table S7.** Primers used for quantitative PCR.
