# Supplementary figures and images for: Chrysomya megacephala larvae feeding favourably influences manure microbiome, heavy metal stability and greenhouse gas emissions
Source: Microb Biotechnol. 2018 Mar 14;11(3):498–509. doi: 10.1111/1751-7915.13253 (PMC5902325; doi:10.1111/1751-7915.13253)

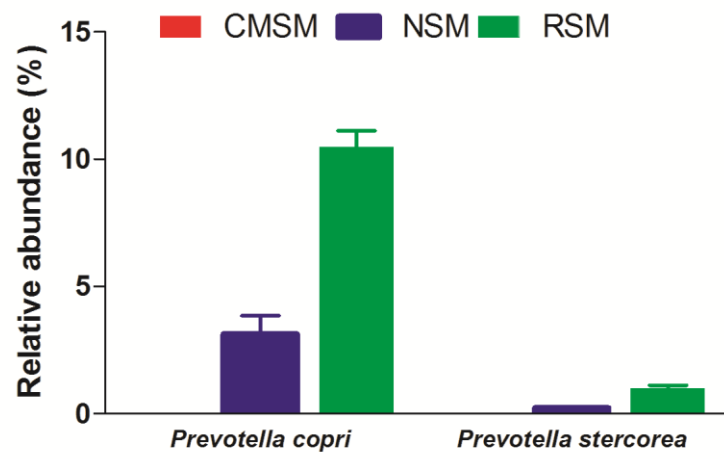

Supplement: Supplementary file 1 — Fig. S1 Relative abundance of Prevotella copri and Prevotella stercorea in RSM, NSM and CMSM. [file MBT2-11-498-s001.pdf]

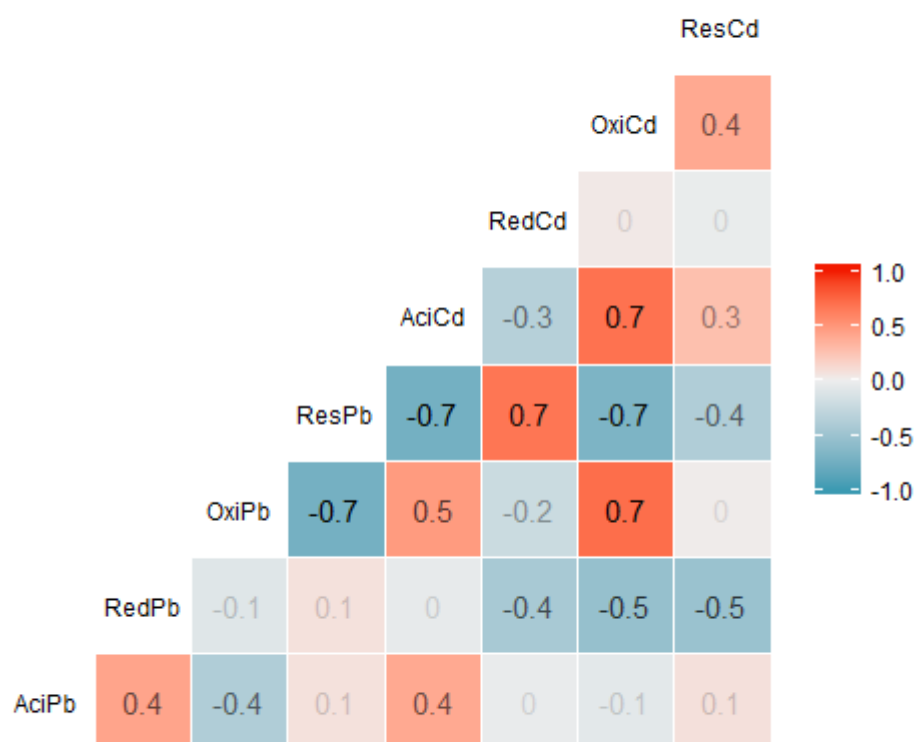

Supplement: Supplementary file 2 — Fig. S2 Correlation coefficient matrix of heavy metal speciation [file MBT2-11-498-s002.pdf]
