# Supplementary material for: Chrysomya megacephala larvae feeding favourably influences manure microbiome, heavy metal stability and greenhouse gas emissions
Source: Microb Biotechnol. 2018 Mar 14;11(3):498–509. doi: 10.1111/1751-7915.13253 (PMC5902325; doi:10.1111/1751-7915.13253)
Supplement: Supplementary file 3 — Table S1 Taxonomy of OTUs. [file MBT2-11-498-s003.pdf]

#OTUId	Abundance	Taxonomy
Otu729	20	Bacteria;Firmicutes;Clostridia;Clostridiales;Ruminococcaceae
Otu1158	85	Bacteria;Proteobacteria;Betaproteobacteria;Burkholderiales;Alcaligenaceae;Alcaligenes;Alcaligenes_faecalis
Otu728	33	Bacteria;Firmicutes;Clostridia;Clostridiales;Ruminococcaceae
Otu937	2	Bacteria;Bacteroidetes;Bacteroidia;Bacteroidales
Otu1081	13	Bacteria;Bacteroidetes;Bacteroidia;Bacteroidales;Prevotellaceae;Prevotella;Prevotella_copri
Otu972	18	Bacteria;Firmicutes;Clostridia;Clostridiales;Ruminococcaceae;Ruminococcus
Otu649	18	Bacteria;Bacteroidetes;Bacteroidia;Bacteroidales;Paraprevotellaceae;Prevotella
Otu573	9	Bacteria;Firmicutes;Clostridia;Clostridiales;Clostridiaceae;Clostridium
Otu985	2	Bacteria;Firmicutes;Clostridia;Clostridiales;Veillonellaceae
Otu714	5	Bacteria;Firmicutes;Clostridia;Clostridiales;Ruminococcaceae;Ruminococcus
Otu828	4	Bacteria;Firmicutes
Otu829	7	Bacteria;Bacteroidetes;Bacteroidia;Bacteroidales
Otu981	6	Bacteria;Bacteroidetes;Bacteroidia;Bacteroidales;S24-7
Otu486	6	Bacteria;Bacteroidetes;Bacteroidia;Bacteroidales;Bacteroidaceae;Bacteroides
Otu983	11	Bacteria;Firmicutes;Clostridia;Clostridiales;Ruminococcaceae
Otu715	3	Bacteria;Firmicutes;Erysipelotrichi;Erysipelotrichales;Erysipelotrichaceae;Erysipelothrix
Otu822	2	Bacteria;Proteobacteria;Deltaproteobacteria;Bdellovibrionales;Bdellovibrionaceae;Bdellovibrio;Bdellovibrio_bacteriovorus
Otu823	2	Bacteria;Firmicutes;Clostridia;Clostridiales;Ruminococcaceae;Clostridium;Clostridium_methylpentosum
Otu820	12	Bacteria;Spirochaetes;Spirochaetes;Spirochaetales;Spirochaetaceae;Treponema
Otu487	24	Bacteria;Tenericutes;Mollicutes;Acholeplasmatales;Acholeplasmataceae;Acholeplasma
Otu826	4	Bacteria;Firmicutes;Bacilli;Bacillales;Bacillaceae
Otu716	9	Bacteria;Firmicutes;Clostridia;Clostridiales
Otu824	160	Bacteria;Firmicutes;Clostridia;Clostridiales;Lachnospiraceae;Roseburia
Otu825	2	Bacteria;Firmicutes;Clostridia;Clostridiales;Ruminococcaceae
Otu488	15	Bacteria;Tenericutes;Mollicutes;Acholeplasmatales;Acholeplasmataceae

Otu717	5	Bacteria;Firmicutes;Clostridia;Clostridiales;Christensenellaceae
Otu489	19	Bacteria;Firmicutes;Clostridia;Clostridiales;Lachnospiraceae
Otu710	12	Bacteria;Firmicutes;Clostridia;Clostridiales;Ruminococcaceae
Otu1137	6	Bacteria;Tenericutes;Mollicutes;RF39
Otu1089	392	Bacteria;Proteobacteria;Gammaproteobacteria;Pseudomonadales;Pseudomonadaceae
Otu723	4	Bacteria;Firmicutes;Clostridia;Clostridiales;Peptococcaceae;rc4-4
Otu711	3	Bacteria;Bacteroidetes;Bacteroidia;Bacteroidales;Porphyromonadaceae;Paludibacter
Otu954	7	Bacteria;Actinobacteria;Actinobacteria;Actinomycetales;Microbacteriaceae
Otu722	8	Bacteria;Firmicutes;Clostridia;Clostridiales;Ruminococcaceae
Otu712	12	Bacteria;Firmicutes;Clostridia;Clostridiales;Lachnospiraceae
Otu725	13	Bacteria;Firmicutes;Clostridia;Clostridiales;Ruminococcaceae
Otu713	5	Bacteria;Firmicutes;Clostridia;Clostridiales;Christensenellaceae
Otu964	6	Bacteria;Firmicutes;Clostridia;Clostridiales;Lachnospiraceae
Otu510	15	Bacteria;Firmicutes;Clostridia;Clostridiales
Otu876	3	Bacteria
Otu727	2	Bacteria;Bacteroidetes;Bacteroidia;Bacteroidales
Otu726	2	Bacteria;Firmicutes;Clostridia;Clostridiales;Tissierellaceae;Tepidimicrobium
Otu766	6	Bacteria;Tenericutes;Mollicutes;Acholeplasmatales;Acholeplasmataceae;Acholeplasma
Otu955	6	Bacteria;Bacteroidetes;Bacteroidia;Bacteroidales;Prevotellaceae;Prevotella
Otu1042	4	Bacteria;Firmicutes;Clostridia;Clostridiales
Otu1043	9	Bacteria;Firmicutes;Clostridia;Clostridiales
Otu529	5	Bacteria;Firmicutes
Otu528	9	Bacteria;Firmicutes;Erysipelotrichi;Erysipelotrichales;Erysipelotrichaceae;Erysipelothrix
Otu1046	2	Bacteria;Acidobacteria;Chloracidobacteria;RB41;Ellin6075
Otu1047	49	Bacteria;Proteobacteria;Gammaproteobacteria;Pseudomonadales;Pseudomonadaceae
Otu1044	2	Bacteria;Firmicutes

Otu1045	3	Bacteria
Otu523	27	Bacteria;Bacteroidetes;Bacteroidia;Bacteroidales;S24-7
Otu522	7	Bacteria;Firmicutes;Clostridia;Clostridiales;Tissierellaceae;Peptoniphilus
Otu521	11	Bacteria;Proteobacteria;Gammaproteobacteria;Pseudomonadales;Pseudomonadaceae
Otu520	10	Bacteria
Otu527	18	Bacteria;Firmicutes;Clostridia;Clostridiales;Clostridiaceae;Clostridium
Otu526	10	Bacteria;Firmicutes;Clostridia;Clostridiales;Acidaminobacteraceae;Guggenheimella
Otu525	21	Bacteria;Firmicutes;Clostridia;Clostridiales;Ruminococcaceae;Ruminococcus
Otu524	18	Bacteria;Bacteroidetes;Flavobacteriia;Flavobacteriales;Flavobacteriaceae;Gelidibacter
Otu1092	7	Bacteria;Firmicutes;Bacilli;Lactobacillales;Aerococcaceae
Otu1076	2	Bacteria;Firmicutes
Otu318	32	Bacteria;Bacteroidetes;Bacteroidia;Bacteroidales;Paraprevotellaceae;Prevotella
Otu319	37	Bacteria;Firmicutes;Clostridia;Clostridiales;Ruminococcaceae;Faecalibacterium
Otu310	127	Bacteria;Firmicutes;Clostridia;Clostridiales
Otu311	31	Bacteria;Firmicutes;Clostridia;Clostridiales;Tissierellaceae
Otu312	25	Bacteria;Firmicutes;Clostridia;Clostridiales;Ruminococcaceae
Otu313	32	Bacteria;Proteobacteria;Gammaproteobacteria;Aeromonadales;Succinivibrionaceae;Succinivibrio
Otu314	50	Bacteria;Firmicutes;Clostridia;Clostridiales;Ruminococcaceae;Oscillospira
Otu315	50	Bacteria;Bacteroidetes;Bacteroidia;Bacteroidales
Otu316	28	Bacteria;Proteobacteria;Gammaproteobacteria;Pseudomonadales;Pseudomonadaceae
Otu317	43	Bacteria;Bacteroidetes;Bacteroidia;Bacteroidales;S24-7
Otu610	42	Bacteria;Firmicutes;Clostridia;Clostridiales;Ruminococcaceae
Otu417	28	Bacteria;Firmicutes;Clostridia;Clostridiales;Christensenellaceae
Otu416	13	Bacteria;Fusobacteria;Fusobacteriia;Fusobacteriales;Fusobacteriaceae;Fusobacterium
Otu415	21	Bacteria;Verrucomicrobia;Verruco-5;WCHB1-41;RFP12
Otu414	21	Bacteria;Firmicutes;Clostridia;Clostridiales;Ruminococcaceae;Oscillospira

Otu413	35	Bacteria;Bacteroidetes;Bacteroidia;Bacteroidales;Porphyromonadaceae;Dysgonomonas
Otu412	12	Bacteria;Firmicutes;AHT28
Otu411	17	Bacteria;Firmicutes;Clostridia;Clostridiales;Lachnospiraceae
Otu410	9	Bacteria;Bacteroidetes;Flavobacteriia;Flavobacteriales;Weeksellaceae
Otu794	2	Bacteria;Firmicutes;Clostridia;Clostridiales;Ruminococcaceae
Otu795	5	Bacteria;Tenericutes;Mollicutes;RF39
Otu796	12	Bacteria;Firmicutes;Clostridia;Clostridiales;Ruminococcaceae
Otu797	8	Bacteria;Firmicutes;Clostridia;Clostridiales;Ruminococcaceae
Otu790	31	Bacteria;Cyanobacteria;4C0d-2;YS2
Otu370	12	Bacteria
Otu419	39	Bacteria;Firmicutes;Clostridia;Clostridiales
Otu418	11	Bacteria;Firmicutes;Clostridia;Clostridiales;Mogibacteriaceae;Mogibacterium
Otu895	182	Bacteria;Firmicutes;Clostridia;Clostridiales;Ruminococcaceae
Otu1036	4	Bacteria;Firmicutes;Clostridia;Clostridiales;Tissierellaceae;GW-34
Otu1035	7	Bacteria;Bacteroidetes;Bacteroidia;Bacteroidales;S24-7
Otu371	16	Bacteria;Tenericutes;Mollicutes;Acholeplasmatales;Acholeplasmataceae;Acholeplasma
Otu1033	3	Bacteria;Firmicutes;Clostridia;Clostridiales;Eubacteriaceae;Anaerofustis
Otu1032	5	Bacteria;Firmicutes;Clostridia;Clostridiales
Otu558	6	Bacteria;Bacteroidetes;Flavobacteriia;Flavobacteriales;Weeksellaceae;Wautersiella
Otu559	15	Bacteria;Firmicutes
Otu556	4	Bacteria;Firmicutes;Bacilli;Haloplasmatales;Haloplasmataceae
Otu557	6	Bacteria;Firmicutes;Clostridia;Clostridiales;Tissierellaceae
Otu554	5	Bacteria;Actinobacteria;Coriobacteriia;Coriobacteriales;Coriobacteriaceae
Otu555	10	Bacteria;Firmicutes;Clostridia;Clostridiales
Otu552	24	Bacteria;Bacteroidetes;Bacteroidia;Bacteroidales;Porphyromonadaceae;Dysgonomonas
Otu553	14	Bacteria;Bacteroidetes;Sphingobacteriia;Sphingobacteriales;Sphingobacteriaceae

Otu550	31	Bacteria;Firmicutes;Clostridia;Clostridiales;Ruminococcaceae;Ruminococcus
Otu551	30	Bacteria;Bacteroidetes;Bacteroidia;Bacteroidales;Prevotellaceae;Prevotella
Otu776	12	Bacteria;Firmicutes;Clostridia;Clostridiales;Lachnospiraceae;Blautia
Otu777	2	Bacteria;Firmicutes;Clostridia;Clostridiales;Lachnospiraceae
Otu774	14	Bacteria;Spirochaetes;Spirochaetes;Sphaerochaetales;Sphaerochaetaceae;Sphaerochaeta
Otu775	6	Bacteria;Firmicutes;Clostridia;Clostridiales;Ruminococcaceae
Otu772	8	Bacteria;Verrucomicrobia;Verruco-5;WCHB1-41;RFP12
Otu773	10	Bacteria;Firmicutes;Clostridia;Clostridiales;Lachnospiraceae;Blautia;Blautia_producta
Otu679	22	Bacteria;Bacteroidetes;Bacteroidia;Bacteroidales;Bacteroidaceae;Bacteroides
Otu678	20	Bacteria;Firmicutes;Erysipelotrichi;Erysipelotrichales;Erysipelotrichaceae;RFN20
Otu677	10	Bacteria;Firmicutes;Clostridia;Clostridiales;Ruminococcaceae
Otu676	7	Bacteria;Actinobacteria;Coriobacteriia;Coriobacteriales;Coriobacteriaceae
Otu675	14	Bacteria;Firmicutes;Clostridia;Clostridiales;Peptostreptococcaceae;Peptostreptococcus
Otu674	18	Bacteria;Proteobacteria;Gammaproteobacteria;Xanthomonadales;Xanthomonadaceae
Otu673	7	Bacteria;Bacteroidetes;Bacteroidia;Bacteroidales;BS11
Otu672	5	Bacteria;Proteobacteria;Betaproteobacteria;Burkholderiales;Alcaligenaceae
Otu671	364	Bacteria;Proteobacteria;Gammaproteobacteria;Pseudomonadales;Pseudomonadaceae
Otu670	11	Bacteria;Firmicutes;Clostridia;Clostridiales;Tissierellaceae;GW-34
Otu58	535	Bacteria;Proteobacteria;Gammaproteobacteria;Alteromonadales;Alteromonadaceae;BD2-13
Otu59	500	Bacteria;Spirochaetes;Spirochaetes;Spirochaetales;Spirochaetaceae;Treponema
Otu986	6	Bacteria;Firmicutes;Clostridia;Clostridiales;Tissierellaceae
Otu389	41	Bacteria;Firmicutes;Clostridia
Otu388	16	Bacteria;Firmicutes;Clostridia;Clostridiales;Ruminococcaceae;Ruminococcus
Otu50	495	Bacteria;Bacteroidetes;Bacteroidia;Bacteroidales;Bacteroidaceae
Otu51	925	Bacteria;Bacteroidetes;Bacteroidia;Bacteroidales;Prevotellaceae;Prevotella
Otu52	1068	Bacteria;Firmicutes;Bacilli;Bacillales;Planococcaceae

Otu53	873	Bacteria;Bacteroidetes;Sphingobacteriia;Sphingobacteriales;Sphingobacteriaceae
Otu54	1636	Bacteria;Firmicutes;Clostridia;Clostridiales;Ruminococcaceae
Otu55	587	Bacteria;Bacteroidetes;Bacteroidia;Bacteroidales
Otu56	755	Bacteria;Bacteroidetes;Bacteroidia;Bacteroidales;Bacteroidaceae;Bacteroides
Otu57	216	Bacteria;Firmicutes;Clostridia;Clostridiales;Ruminococcaceae;Oscillospira
Otu379	16	Bacteria;Firmicutes;Clostridia;Clostridiales;Ruminococcaceae
Otu982	2	Bacteria;Firmicutes;Clostridia;Clostridiales
Otu251	44	Bacteria;Proteobacteria;Gammaproteobacteria;Enterobacteriales;Enterobacteriaceae
Otu250	74	Bacteria;Proteobacteria;Alphaproteobacteria;Sphingomonadales;Sphingomonadaceae;Sphingopyxis
Otu253	36	Bacteria;Bacteroidetes;Bacteroidia;Bacteroidales;Paraprevotellaceae;Prevotella
Otu252	62	Bacteria;Bacteroidetes;Bacteroidia;Bacteroidales;Prevotellaceae;Prevotella
Otu255	90	Bacteria;Firmicutes;Erysipelotrichi;Erysipelotrichales;Erysipelotrichaceae;Erysipelothrix
Otu254	120	Bacteria;Bacteroidetes;Bacteroidia;Bacteroidales;Prevotellaceae;Prevotella
Otu257	38	Bacteria;Bacteroidetes
Otu256	69	Bacteria;Bacteroidetes;Bacteroidia;Bacteroidales;Porphyromonadaceae;Dysgonomonas
Otu259	49	Bacteria;Bacteroidetes;Sphingobacteriia;Sphingobacteriales;Sphingobacteriaceae;Sphingobacterium
Otu258	49	Bacteria;Bacteroidetes;Bacteroidia;Bacteroidales;Bacteroidaceae
Otu709	7	Bacteria;Cyanobacteria;4C0d-2;YS2
Otu708	5	Bacteria;Bacteroidetes;Bacteroidia;Bacteroidales
Otu1139	4	Bacteria;Proteobacteria;Epsilonproteobacteria;Campylobacterales;Helicobacteraceae
Otu1111	3	Bacteria;Firmicutes;Clostridia;Clostridiales
Otu589	6	Bacteria;Firmicutes;AHT28
Otu821	4	Bacteria;Firmicutes;Clostridia;Clostridiales
Otu1034	4	Bacteria;Tenericutes;Mollicutes;RF39
Otu989	2	Bacteria;Firmicutes;Clostridia;Clostridiales;Lachnospiraceae
Otu396	54	Bacteria;Firmicutes;Clostridia;Clostridiales;Lachnospiraceae;Dorea

Otu827	2	Bacteria;Firmicutes;Clostridia;Clostridiales;Tissierellaceae;Tissierella_Soehngenia
Otu1109	4	Bacteria;Bacteroidetes;Bacteroidia;Bacteroidales;Prevotellaceae;Prevotella
Otu1136	42	Bacteria;Firmicutes;Clostridia;Clostridiales;Ruminococcaceae
Otu966	7	Bacteria;Firmicutes;Bacilli;Bacillales;Bacillaceae
Otu29	1210	Bacteria
Otu28	1073	Bacteria;Bacteroidetes;Flavobacteriia;Flavobacteriales;Weeksellaceae;Wautersiella
Otu25	4152	Bacteria;Proteobacteria;Gammaproteobacteria;Pseudomonadales;Moraxellaceae;Acinetobacter
Otu24	2687	Bacteria;Firmicutes;Clostridia;Clostridiales;Veillonellaceae;Megasphaera
Otu27	1107	Bacteria;Fibrobacteres;Fibrobacteria;Fibrobacterales;Fibrobacteraceae;Fibrobacter;Fibrobacter_succinogenes
Otu26	988	Archaea;Euryarchaeota;Methanobacteria;Methanobacteriales;Methanobacteriaceae;Methanobrevibacter
Otu21	1337	Bacteria;Firmicutes;Bacilli;Bacillales;Planococcaceae;Rummeliibacillus
Otu20	2759	Bacteria;Bacteroidetes;Bacteroidia;Bacteroidales;S24-7
Otu23	1771	Bacteria;Bacteroidetes
Otu22	2028	Bacteria;Bacteroidetes;Bacteroidia;Bacteroidales;Marinilabiaceae
Otu174	102	Bacteria;Tenericutes;Mollicutes;Acholeplasmatales;Acholeplasmataceae;Acholeplasma;Acholeplasma_laidlawii
Otu175	116	Bacteria;Firmicutes;Clostridia;Clostridiales;Ruminococcaceae;Oscillospira
Otu176	78	Bacteria;Tenericutes;Mollicutes;Acholeplasmatales;Acholeplasmataceae;Acholeplasma
Otu177	68	Bacteria;Bacteroidetes;Bacteroidia;Bacteroidales;Porphyromonadaceae;Dysgonomonas
Otu170	113	Bacteria;Firmicutes;Clostridia;Clostridiales
Otu171	80	Bacteria;Proteobacteria;Betaproteobacteria;Burkholderiales;Alcaligenaceae
Otu172	136	Bacteria;Proteobacteria;Gammaproteobacteria;Enterobacteriales;Enterobacteriaceae;Escherichia;Escherichia_coli
Otu173	186	Bacteria;Bacteroidetes;Bacteroidia;Bacteroidales;Paraprevotellaceae
Otu1110	11	Bacteria;Firmicutes;Clostridia;Clostridiales;Ruminococcaceae
Otu178	217	Bacteria;Bacteroidetes;Bacteroidia;Bacteroidales;Prevotellaceae;Prevotella
Otu179	147	Bacteria;Firmicutes;Clostridia;Clostridiales;Ruminococcaceae
Otu518	12	Bacteria;Actinobacteria;Coriobacteriia;Coriobacteriales;Coriobacteriaceae

Otu1112	177	Bacteria;Proteobacteria;Betaproteobacteria;Burkholderiales;Comamonadaceae
Otu991	2	Bacteria;Bacteroidetes;Bacteroidia;Bacteroidales;Prevotellaceae;Prevotella
Otu898	10	Bacteria;Tenericutes;Mollicutes;Anaeroplasmatales;Anaeroplasmataceae;Anaeroplasma
Otu908	3	Bacteria;Firmicutes;Clostridia;Clostridiales
Otu789	4	Bacteria;Spirochaetes;Spirochaetes;Spirochaetales;Spirochaetaceae;Treponema
Otu962	3	Bacteria;Firmicutes;Erysipelotrichi;Erysipelotrichales;Erysipelotrichaceae;p-75-a5
Otu435	43	Bacteria;Firmicutes;Clostridia;Clostridiales;Ruminococcaceae
Otu1084	2	Bacteria;Firmicutes;Clostridia;Clostridiales
Otu788	529	Bacteria;Bacteroidetes;Bacteroidia;Bacteroidales;Porphyromonadaceae
Otu1082	2	Bacteria;Proteobacteria;Gammaproteobacteria
Otu1083	2	Bacteria;Verrucomicrobia;Verrucomicrobiae;Verrucomicrobiales;Verrucomicrobiaceae;Luteolibacter
Otu1080	7	Bacteria;Bacteroidetes;Bacteroidia;Bacteroidales;Porphyromonadaceae;Dysgonomonas
Otu434	27	Bacteria;Firmicutes;Erysipelotrichi;Erysipelotrichales;Erysipelotrichaceae;L7A_E11
Otu943	2	Bacteria;Firmicutes;Clostridia;Clostridiales;Ruminococcaceae
Otu437	16	Bacteria;Firmicutes;Clostridia;Clostridiales;Ruminococcaceae
Otu1088	2	Bacteria;Firmicutes;Bacilli
Otu1031	19	Bacteria;Proteobacteria;Betaproteobacteria;Burkholderiales;Alcaligenaceae
Otu875	4	Bacteria;Proteobacteria;Alphaproteobacteria;RF32
Otu874	9	Bacteria;Actinobacteria;Actinobacteria;Actinomycetales;Microbacteriaceae
Otu877	3	Bacteria;Firmicutes;Clostridia;Clostridiales;Lachnospiraceae;Blautia
Otu436	36	Bacteria;Actinobacteria;Actinobacteria;Actinomycetales;Microbacteriaceae;Leucobacter
Otu871	8	Bacteria;Tenericutes;Mollicutes;Acholeplasmatales;Acholeplasmataceae;Acholeplasma;Acholeplasma_laidlawii
Otu870	26	Bacteria;Firmicutes;Clostridia;Clostridiales;Lachnospiraceae;Blautia
Otu873	15	Bacteria;Bacteroidetes;Bacteroidia;Bacteroidales;Bacteroidaceae;Bacteroides
Otu872	17	Bacteria;Firmicutes;Clostridia;Clostridiales;Ruminococcaceae
Otu1106	5	Bacteria;Bacteroidetes;Bacteroidia;Bacteroidales;Bacteroidaceae;Bacteroides;Bacteroides_coprosuis

Otu431	2005	Bacteria;Bacteroidetes;Bacteroidia;Bacteroidales;Prevotellaceae;Prevotella
Otu879	2	Bacteria;Firmicutes;Clostridia;Clostridiales;Ruminococcaceae;Oscillospira
Otu878	2	Bacteria;Firmicutes;Clostridia;Clostridiales;Lachnospiraceae;Dorea
Otu745	8	Bacteria;Firmicutes;Clostridia;Clostridiales;Tissierellaceae;GW-34
Otu430	17	Bacteria;Cyanobacteria;4C0d-2;YS2
Otu926	2	Bacteria
Otu433	10	Bacteria;Bacteroidetes;Bacteroidia;Bacteroidales
Otu707	11	Bacteria;Bacteroidetes;Bacteroidia;Bacteroidales;S24-7
Otu432	39	Bacteria;Cyanobacteria;4C0d-2;YS2
Otu781	12	Bacteria;Verrucomicrobia;Verruco-5;WCHB1-41;RFP12
Otu268	59	Bacteria;Actinobacteria;Actinobacteria;Actinomycetales;Corynebacteriaceae;Corynebacterium
Otu588	19	Bacteria;Firmicutes;Clostridia;Clostridiales;Tissierellaceae
Otu780	24	Bacteria;Firmicutes;Erysipelotrichi;Erysipelotrichales;Erysipelotrichaceae;Erysipelothrix
Otu1147	3	Bacteria;Firmicutes;Clostridia;Clostridiales
Otu581	8	Bacteria;Firmicutes;Clostridia;Clostridiales;Lachnospiraceae;Coprococcus
Otu580	14	Bacteria;Firmicutes;Clostridia;Clostridiales;Clostridiaceae;Clostridium
Otu583	25	Bacteria;Firmicutes;Clostridia;Clostridiales;Lachnospiraceae
Otu582	20	Bacteria;Firmicutes;Clostridia;Clostridiales;Lachnospiraceae
Otu585	3	Bacteria;Proteobacteria;Gammaproteobacteria
Otu584	13	Bacteria;Firmicutes
Otu587	7	Bacteria;Bacteroidetes;Bacteroidia;Bacteroidales;Prevotellaceae
Otu586	14	Bacteria;Firmicutes;Clostridia;Clostridiales;Clostridiaceae;Clostridium
Otu656	12	Bacteria;Firmicutes;Clostridia;Clostridiales;Veillonellaceae;Megasphaera
Otu1059	2	Bacteria;Tenericutes;Mollicutes;RF39
Otu512	40	Bacteria;Firmicutes;Clostridia;Clostridiales
Otu513	11	Bacteria;Firmicutes;Clostridia;Clostridiales

Otu459	24	Bacteria;Firmicutes;Clostridia;Clostridiales;Clostridiaceae;SMB53
Otu458	997	Bacteria;Proteobacteria;Gammaproteobacteria;Aeromonadales;Succinivibrionaceae;Succinivibrio
Otu516	14	Bacteria;Firmicutes;Clostridia;Clostridiales
Otu517	136	Bacteria;Firmicutes;Clostridia;Clostridiales;Ruminococcaceae;Ruminococcus
Otu514	13	Bacteria;Firmicutes;Clostridia;Clostridiales;Lachnospiraceae
Otu515	15	Bacteria;Firmicutes;Clostridia;Clostridiales;Lachnospiraceae
Otu453	27	Bacteria;Firmicutes;Clostridia;Clostridiales;Lachnospiraceae;Coprococcus;Coprococcus_eutactus
Otu452	50	Bacteria;Firmicutes;Clostridia;Clostridiales
Otu451	23	Bacteria;Bacteroidetes;Bacteroidia;Bacteroidales;Marinilabiaceae;Ruminofilibacter;Ruminofilibacter_xylanolyticum
Otu450	20	Bacteria;Actinobacteria;Coriobacteriia;Coriobacteriales;Coriobacteriaceae
Otu457	25	Bacteria;Bacteroidetes;Bacteroidia;Bacteroidales;Porphyromonadaceae;Dysgonomonas
Otu456	87	Bacteria;Firmicutes;Clostridia;Clostridiales
Otu455	28	Bacteria;Firmicutes;Clostridia;Clostridiales;Lachnospiraceae
Otu454	22	Bacteria;Firmicutes;Clostridia;Clostridiales;Lachnospiraceae;Blautia
Otu950	2	Bacteria;Cyanobacteria;4C0d-2;YS2
Otu1079	2	Bacteria;Bacteroidetes;Bacteroidia;Bacteroidales;Prevotellaceae
Otu1078	3	Bacteria;Proteobacteria;Gammaproteobacteria
Otu397	28	Bacteria
Otu1020	8	Bacteria;Actinobacteria;Actinobacteria;Actinomycetales;Corynebacteriaceae;Corynebacterium
Otu1071	2	Bacteria;Bacteroidetes;Bacteroidia;Bacteroidales
Otu1070	2	Bacteria;Bacteroidetes
Otu1077	65	Bacteria;Firmicutes;Clostridia;Clostridiales;Tissierellaceae;Peptoniphilus
Otu1058	12	Bacteria;Firmicutes;Clostridia;Clostridiales;Ruminococcaceae
Otu1075	4	Bacteria;Tenericutes;Mollicutes;RF39
Otu1021	7	Bacteria;Firmicutes;Clostridia;Clostridiales;Lachnospiraceae
Otu228	182	Bacteria;Firmicutes;Clostridia;Clostridiales

Otu229	268	Bacteria;Firmicutes;Clostridia;Clostridiales;Ruminococcaceae
Otu738	2	Bacteria;Firmicutes;Clostridia;Clostridiales;Ruminococcaceae
Otu739	25	Bacteria;Bacteroidetes;Bacteroidia;Bacteroidales;Prevotellaceae;Prevotella
Otu904	4	Bacteria;Firmicutes;Clostridia;Clostridiales;Christensenellaceae
Otu884	2	Bacteria;Bacteroidetes;Bacteroidia;Bacteroidales;S24-7
Otu220	20	Bacteria;Proteobacteria;Betaproteobacteria;Rhodocyclales;Rhodocyclaceae
Otu221	78	Bacteria;Actinobacteria;Actinobacteria;Actinomycetales;Microbacteriaceae;Leucobacter
Otu222	95	Bacteria;Proteobacteria;Deltaproteobacteria;Desulfovibrionales;Desulfovibrionaceae;Desulfovibrio;Desulfovibrio_D168
Otu223	165	Bacteria;Proteobacteria;Gammaproteobacteria
Otu224	91	Bacteria;Firmicutes;Clostridia;Clostridiales;Ruminococcaceae;Ruminococcus
Otu225	82	Bacteria;Bacteroidetes;Bacteroidia;Bacteroidales;Bacteroidaceae;Bacteroides;Bacteroides_coprosuis
Otu226	49	Bacteria;Firmicutes;Clostridia;Clostridiales;Mogibacteriaceae
Otu227	58	Bacteria;Firmicutes;Erysipelotrichi;Erysipelotrichales;Erysipelotrichaceae
Otu1143	58	Bacteria;Bacteroidetes;Bacteroidia;Bacteroidales;Prevotellaceae;Prevotella;Prevotella_copri
Otu511	12	Bacteria;Proteobacteria;Gammaproteobacteria
Otu927	2	Bacteria;Firmicutes;Clostridia;Clostridiales
Otu1141	6	Bacteria;Firmicutes;Erysipelotrichi;Erysipelotrichales;Erysipelotrichaceae
Otu938	67	Bacteria;Bacteroidetes;Bacteroidia;Bacteroidales;Porphyromonadaceae
Otu907	2	Bacteria;Deferribacteres;Deferribacteres;Deferribacterales;Deferribacteraceae;Geovibrio;Geovibrio_thiophilus
Otu1140	7	Bacteria;Tenericutes;Mollicutes;RF39
Otu980	2	Bacteria;Firmicutes;Clostridia;Clostridiales;Clostridiaceae;Clostridium
Otu965	2	Bacteria;Bacteroidetes;Bacteroidia;Bacteroidales
Otu939	2	Bacteria;Firmicutes;Clostridia
Otu1028	2	Bacteria;Bacteroidetes;Bacteroidia;Bacteroidales;Prevotellaceae;Prevotella
Otu1131	2	Bacteria;Firmicutes;Clostridia;Clostridiales;Ruminococcaceae
Otu1029	5	Bacteria;Proteobacteria;Gammaproteobacteria;Enterobacteriales;Enterobacteriaceae

Otu1107	6	Bacteria;Firmicutes;Clostridia;Clostridiales;Lachnospiraceae;Ruminococcus;Ruminococcus_torques
Otu942	2	Bacteria;Tenericutes;Mollicutes;Acholeplasmatales;Acholeplasmataceae;Acholeplasma
Otu1105	17	Bacteria;Firmicutes;Clostridia;Clostridiales;Lachnospiraceae
Otu1104	2	Bacteria;Tenericutes;Mollicutes;RF39
Otu358	54	Bacteria;Bacteroidetes;Flavobacteriia;Flavobacteriales;Weeksellaceae
Otu359	15	Bacteria;Firmicutes;Clostridia;Clostridiales;Lachnospiraceae
Otu428	9	Bacteria;Cyanobacteria;4C0d-2;YS2
Otu429	235	Bacteria;Bacteroidetes;Sphingobacteriia;Sphingobacteriales;Sphingobacteriaceae
Otu354	27	Bacteria;Tenericutes;Mollicutes;RF39
Otu355	32	Bacteria;Bacteroidetes;Bacteroidia;Bacteroidales
Otu356	93	Bacteria;Proteobacteria;Gammaproteobacteria;Alteromonadales;Alteromonadaceae;Candidatus_Endobugula
Otu357	29	Bacteria;Bacteroidetes;Bacteroidia;Bacteroidales;S24-7
Otu350	61	Bacteria;Bacteroidetes;Bacteroidia;Bacteroidales;Prevotellaceae;Prevotella
Otu351	29	Bacteria;Firmicutes;Erysipelotrichi;Erysipelotrichales;Erysipelotrichaceae
Otu352	27	Bacteria;Tenericutes;Mollicutes;RF39
Otu353	26	Bacteria;Firmicutes;Clostridia;Clostridiales;Ruminococcaceae
Otu646	3	Bacteria;Firmicutes;Clostridia;Clostridiales;Ruminococcaceae
Otu647	69	Bacteria;Bacteroidetes;Flavobacteriia;Flavobacteriales;Flavobacteriaceae
Otu644	9	Bacteria;Bacteroidetes;Bacteroidia;Bacteroidales;Porphyromonadaceae
Otu519	12	Bacteria;Cyanobacteria;4C0d-2;YS2
Otu642	7	Bacteria;Bacteroidetes;Bacteroidia;Bacteroidales;S24-7
Otu643	3	Bacteria
Otu129	161	Bacteria;Bacteroidetes;Bacteroidia;Bacteroidales;Bacteroidaceae;Bacteroides
Otu128	325	Bacteria;Firmicutes;Bacilli;Bacillales;Planococcaceae
Otu127	87	Bacteria;Bacteroidetes;Flavobacteriia;Flavobacteriales;Flavobacteriaceae
Otu126	168	Bacteria;Bacteroidetes;Bacteroidia;Bacteroidales;Prevotellaceae;Prevotella

Otu125	352	Bacteria;Bacteroidetes;Bacteroidia;Bacteroidales;Porphyromonadaceae
Otu124	171	Bacteria;Bacteroidetes;Bacteroidia;Bacteroidales;S24-7
Otu123	157	Bacteria;Proteobacteria;Deltaproteobacteria;Bdellovibrionales;Bacteriovoracaceae
Otu122	203	Bacteria;Firmicutes;Clostridia;Clostridiales;Ruminococcaceae
Otu121	179	Bacteria;Proteobacteria;Gammaproteobacteria
Otu120	298	Bacteria;Bacteroidetes;Bacteroidia;Bacteroidales;RF16
Otu61	1945	Bacteria;Bacteroidetes;Bacteroidia;Bacteroidales;Prevotellaceae;Prevotella;Prevotella_copri
Otu60	393	Bacteria;Firmicutes;Clostridia;Clostridiales;Veillonellaceae;Succiniclasticum
Otu63	742	Bacteria;Bacteroidetes;Bacteroidia;Bacteroidales
Otu62	561	Bacteria;Bacteroidetes;Bacteroidia;Bacteroidales
Otu65	843	Bacteria;Proteobacteria;Betaproteobacteria;Burkholderiales;Alcaligenaceae
Otu64	695	Bacteria;Proteobacteria;Betaproteobacteria;Burkholderiales;Alcaligenaceae;Alcaligenes;Alcaligenes_faecalis
Otu67	653	Bacteria;Bacteroidetes;Bacteroidia;Bacteroidales;Prevotellaceae;Prevotella;Prevotella_stercorea
Otu66	801	Bacteria;Bacteroidetes;Flavobacteriia;Flavobacteriales;Weeksellaceae
Otu69	414	Bacteria;Proteobacteria;Betaproteobacteria;Burkholderiales;Alcaligenaceae
Otu68	528	Bacteria;Firmicutes;Clostridia;Clostridiales;Tissierellaceae;GW-34
Otu747	34	Bacteria;Bacteroidetes;Bacteroidia;Bacteroidales;Paraprevotellaceae;Prevotella
Otu906	3	Bacteria;Firmicutes
Otu901	4	Bacteria;Firmicutes;Clostridia;Clostridiales;Lachnospiraceae
Otu900	12	Bacteria;Bacteroidetes;Bacteroidia;Bacteroidales;Prevotellaceae;Prevotella
Otu903	2	Bacteria;Firmicutes;Clostridia;Clostridiales
Otu902	7	Bacteria;Firmicutes;Clostridia;Clostridiales;Ruminococcaceae;Oscillospira
Otu933	2	Bacteria;Actinobacteria;Actinobacteria;Actinomycetales;Mycobacteriaceae;Mycobacterium
Otu947	2	Bacteria;Firmicutes;Clostridia;Clostridiales;Mogibacteriaceae;Mogibacterium
Otu1161	5	Bacteria;Firmicutes;Clostridia;Clostridiales;Lachnospiraceae
Otu946	4	Bacteria;Firmicutes;Clostridia;Clostridiales;Lachnospiraceae

Otu633	10	Bacteria;Firmicutes;Erysipelotrichi;Erysipelotrichales;Erysipelotrichaceae;Catenibacterium
Otu632	57	Bacteria;Bacteroidetes;Bacteroidia;Bacteroidales;Prevotellaceae;Prevotella;Prevotella_copri
Otu631	3	Bacteria;Bacteroidetes;Bacteroidia;Bacteroidales
Otu630	5	Bacteria;Firmicutes;Clostridia;Clostridiales;Ruminococcaceae
Otu637	9	Bacteria;Firmicutes;Clostridia;Clostridiales;Ruminococcaceae;Ruminococcus
Otu636	13	Bacteria;Firmicutes;Clostridia;Clostridiales;Ruminococcaceae
Otu635	18	Bacteria;Verrucomicrobia;Opitutae
Otu634	133	Bacteria;Bacteroidetes;Bacteroidia;Bacteroidales;Prevotellaceae;Prevotella;Prevotella_copri
Otu639	7	Bacteria;Firmicutes;Clostridia;Clostridiales;Lachnospiraceae;Coprococcus
Otu638	17	Bacteria;Tenericutes;Mollicutes;RF39
Otu1146	6	Bacteria;Firmicutes;Clostridia;Clostridiales;Ruminococcaceae;Oscillospira
Otu769	10	Bacteria;Synergistetes;Synergistia;Synergistales;Dethiosulfovibrionaceae
Otu1040	12	Bacteria;Firmicutes;Clostridia;Clostridiales;Ruminococcaceae
Otu768	2	Bacteria;Firmicutes;Clostridia;Clostridiales
Otu791	3	Bacteria;Firmicutes;Clostridia;Clostridiales
Otu1041	337	Bacteria;Bacteroidetes;Bacteroidia;Bacteroidales;Porphyromonadaceae
Otu1072	2	Bacteria;Firmicutes;Clostridia;Clostridiales
Otu839	8	Bacteria;Proteobacteria;Alphaproteobacteria;RF32
Otu838	2	Bacteria;Firmicutes;Clostridia;Clostridiales;Ruminococcaceae;Ruminococcus
Otu831	8	Bacteria;Firmicutes;Clostridia;Clostridiales;Lachnospiraceae;Dorea
Otu830	3	Bacteria;Bacteroidetes;Bacteroidia;Bacteroidales;Porphyromonadaceae
Otu833	2	Bacteria;Firmicutes;Erysipelotrichi;Erysipelotrichales;Erysipelotrichaceae;p-75-a5
Otu832	9	Bacteria;Firmicutes;Clostridia;Clostridiales;Ruminococcaceae
Otu835	13	Bacteria;Cyanobacteria;4C0d-2;YS2
Otu834	3	Bacteria;Firmicutes;Clostridia;Clostridiales;Tissierellaceae
Otu837	2	Bacteria;Firmicutes;Clostridia;Clostridiales;Lachnospiraceae

Otu836	3	Bacteria;Bacteroidetes;Bacteroidia;Bacteroidales;Prevotellaceae;Prevotella
Otu923	460	Bacteria
Otu967	11	Bacteria;Firmicutes;Clostridia;Clostridiales;Lachnospiraceae
Otu1074	2	Bacteria;Bacteroidetes;Bacteroidia;Bacteroidales;Prevotellaceae
Otu1048	21	Bacteria;Firmicutes;Clostridia;Clostridiales;Lachnospiraceae
Otu1049	30	Bacteria;Firmicutes;Clostridia;Clostridiales;Lachnospiraceae
Otu496	36	Bacteria;Tenericutes;Mollicutes;RF39
Otu945	2	Bacteria;Firmicutes;Clostridia;Clostridiales;Lachnospiraceae
Otu885	2	Bacteria;Firmicutes;Clostridia;Clostridiales;Ruminococcaceae;Oscillospira
Otu1085	3	Bacteria;Firmicutes;Clostridia;Clostridiales
Otu922	32	Bacteria;Firmicutes;Clostridia;Clostridiales;Lachnospiraceae
Otu495	16	Bacteria;Actinobacteria;Actinobacteria;Actinomycetales
Otu921	6	Bacteria;Firmicutes;Clostridia;Clostridiales
Otu732	5	Bacteria
Otu733	2	Bacteria;Bacteroidetes
Otu730	56	Bacteria;Bacteroidetes;Bacteroidia;Bacteroidales;Bacteroidaceae;Bacteroides;Bacteroides_coprosuis
Otu1134	14	Bacteria;Bacteroidetes;Sphingobacteriia;Sphingobacteriales;Sphingobacteriaceae;Sphingobacterium
Otu1099	12	Bacteria;Tenericutes;Mollicutes;RF39
Otu731	2	Bacteria;Bacteroidetes;Bacteroidia;Bacteroidales;Prevotellaceae;Prevotella
Otu887	5	Bacteria;Firmicutes;Clostridia;Clostridiales
Otu1098	2	Bacteria;Firmicutes;Clostridia;Clostridiales;Ruminococcaceae;Oscillospira
Otu736	2	Bacteria;Firmicutes;Clostridia;Clostridiales;Tissierellaceae;Sporanaerobacter
Otu721	8	Bacteria;Firmicutes;Clostridia;Clostridiales
Otu1116	71	Bacteria;Firmicutes;Clostridia;Clostridiales;Ruminococcaceae;Ruminococcus
Otu751	10	Bacteria;Bacteroidetes;Bacteroidia;Bacteroidales;Prevotellaceae;Prevotella
Otu737	16	Bacteria;Firmicutes;Clostridia;Clostridiales;Ruminococcaceae

Otu1030	2	Bacteria;Firmicutes
Otu1117	3	Bacteria;Firmicutes;Clostridia;Clostridiales;Lachnospiraceae
Otu734	3	Bacteria;Firmicutes;Clostridia;Clostridiales;Tissierellaceae;Sedimentibacter
Otu988	8	Bacteria;Firmicutes;Clostridia;Clostridiales;Ruminococcaceae;Oscillospira
Otu735	7	Bacteria;Firmicutes;Clostridia;Clostridiales;Veillonellaceae
Otu1159	2	Bacteria;Firmicutes;Bacilli;Lactobacillales;Lactobacillaceae;Lactobacillus
Otu462	16	Bacteria;Firmicutes;Clostridia;Clostridiales;Ruminococcaceae
Otu463	13	Bacteria;Firmicutes;Clostridia;MBA08
Otu460	13	Bacteria;Thermi;Deinococci;Deinococcales;Trueperaceae;B-42
Otu461	15	Bacteria;Bacteroidetes;Flavobacteriia;Flavobacteriales;Flavobacteriaceae
Otu466	17	Bacteria;Firmicutes;Clostridia;Clostridiales;Tissierellaceae;Gallicola
Otu467	682	Bacteria;Bacteroidetes;Bacteroidia;Bacteroidales;Bacteroidaceae;Bacteroides;Bacteroides_coprosuis
Otu464	12	Bacteria;Firmicutes;Clostridia;Clostridiales;Lachnospiraceae;Coprococcus
Otu465	9	Bacteria;Tenericutes;Mollicutes;RF39
Otu783	9	Bacteria;Firmicutes;Clostridia;Clostridiales
Otu782	15	Bacteria;Firmicutes;Erysipelotrichi;Erysipelotrichales;Erysipelotrichaceae;p-75-a5
Otu468	13	Bacteria;Bacteroidetes;Bacteroidia;Bacteroidales;Porphyromonadaceae;Parabacteroides
Otu469	28	Bacteria;Firmicutes;Clostridia;Clostridiales;Ruminococcaceae
Otu787	2	Bacteria;Bacteroidetes;Bacteroidia;Bacteroidales;Prevotellaceae
Otu786	2	Bacteria;Bacteroidetes;Bacteroidia;Bacteroidales;Prevotellaceae;Prevotella
Otu785	3	Bacteria;Bacteroidetes
Otu784	111	Bacteria;Bacteroidetes;Bacteroidia;Bacteroidales;Prevotellaceae;Prevotella;Prevotella_copri
Otu1024	4	Bacteria;Firmicutes;Erysipelotrichi;Erysipelotrichales;Erysipelotrichaceae;Erysipelothrix
Otu1025	3	Bacteria;Actinobacteria;Actinobacteria;Actinomycetales
Otu1026	8	Bacteria;Firmicutes;Clostridia;Clostridiales;Lachnospiraceae
Otu1027	20	Bacteria;Bacteroidetes;Bacteroidia;Bacteroidales;Prevotellaceae;Prevotella

Otu549	15	Bacteria;Bacteroidetes;Sphingobacteriia;Sphingobacteriales;Sphingobacteriaceae
Otu548	14	Bacteria;Proteobacteria;Deltaproteobacteria;GMD14H09
Otu1022	4	Bacteria
Otu1023	7	Bacteria;Firmicutes;Clostridia;Clostridiales;Lachnospiraceae
Otu545	20	Bacteria;Verrucomicrobia;Verrucomicrobiae;Verrucomicrobiales;Verrucomicrobiaceae;Akkermansia
Otu544	80	Bacteria;Proteobacteria;Alphaproteobacteria;Rhodobacterales;Rhodobacteraceae
Otu547	17	Bacteria;Firmicutes;Clostridia;Clostridiales;Lachnospiraceae;Coprococcus;Coprococcus_catus
Otu546	21	Bacteria;Verrucomicrobia;Verrucomicrobiae;Verrucomicrobiales;Verrucomicrobiaceae;Akkermansia;Akkermansia_muciniphila
Otu541	30	Bacteria;Tenericutes;Mollicutes;RF39
Otu540	20	Bacteria;Firmicutes;Clostridia;Clostridiales;Lachnospiraceae
Otu543	13	Bacteria;Firmicutes;Erysipelotrichi;Erysipelotrichales;Erysipelotrichaceae
Otu542	18	Bacteria;Firmicutes;Clostridia;Clostridiales;Tissierellaceae;Tissierella_Soehngenia
Otu765	2	Bacteria;Firmicutes;Clostridia;Clostridiales
Otu764	13	Bacteria;Firmicutes;Clostridia;Clostridiales
Otu608	3	Bacteria;Firmicutes;Clostridia;Clostridiales
Otu609	25	Bacteria;Firmicutes;Clostridia;Clostridiales;Peptostreptococcaceae
Otu761	3	Bacteria;Firmicutes;Clostridia;Clostridiales;Ruminococcaceae;Ruminococcus
Otu760	2	Bacteria;Firmicutes
Otu763	10	Bacteria;Firmicutes;Clostridia;Clostridiales;Lachnospiraceae
Otu762	176	Bacteria;Bacteroidetes;Bacteroidia;Bacteroidales
Otu602	11	Bacteria;Verrucomicrobia;Verruco-5;WCHB1-41;RFP12
Otu603	35	Bacteria;Bacteroidetes;Bacteroidia;Bacteroidales;Prevotellaceae;Prevotella
Otu600	10	Bacteria;Proteobacteria;Gammaproteobacteria
Otu601	13	Bacteria;Firmicutes;Clostridia;Clostridiales;Mogibacteriaceae;Anaerovorax
Otu606	5	Bacteria;Proteobacteria;Deltaproteobacteria;Desulfovibrionales;Desulfovibrionaceae
Otu607	11	Bacteria;Bacteroidetes;Bacteroidia;Bacteroidales;S24-7

Otu604	86	Bacteria;Firmicutes;Clostridia;Clostridiales;Ruminococcaceae
Otu605	3	Bacteria;Firmicutes;Erysipelotrichi;Erysipelotrichales;Erysipelotrichaceae;RFN20
Otu1157	85	Bacteria;Firmicutes;Clostridia;Clostridiales;Tissierellaceae
Otu1097	4	Bacteria;Actinobacteria;Actinobacteria;Bifidobacteriales;Bifidobacteriaceae
Otu1096	10	Bacteria;Proteobacteria;Gammaproteobacteria;Pseudomonadales;Moraxellaceae;Acinetobacter
Otu611	6	Bacteria;Bacteroidetes;Bacteroidia;Bacteroidales;Porphyromonadaceae;Parabacteroides
Otu1130	5	Bacteria;Firmicutes;Clostridia;Clostridiales
Otu307	73	Bacteria;Bacteroidetes;Bacteroidia;Bacteroidales;Paraprevotellaceae;CF231
Otu306	54	Bacteria;Firmicutes;Clostridia;Clostridiales;Christensenellaceae
Otu305	49	Bacteria;Bacteroidetes;Bacteroidia;Bacteroidales
Otu304	49	Bacteria;Firmicutes;Clostridia;Clostridiales
Otu303	32	Bacteria;Firmicutes;Clostridia;Clostridiales;Ruminococcaceae
Otu269	24	Bacteria;Firmicutes;Clostridia;Clostridiales;Ruminococcaceae
Otu301	61	Bacteria;Firmicutes;Clostridia;Clostridiales;Clostridiaceae;Proteiniclasticum
Otu300	44	Bacteria;Firmicutes;Clostridia;Clostridiales;Ruminococcaceae;Ruminococcus
Otu264	56	Bacteria;Firmicutes;Erysipelotrichi;Erysipelotrichales;Erysipelotrichaceae;Erysipelothrix
Otu265	125	Bacteria;Firmicutes;Bacilli;Lactobacillales;Streptococcaceae;Streptococcus
Otu266	92	Bacteria;Bacteroidetes;Bacteroidia;Bacteroidales;Bacteroidaceae;Bacteroides;Bacteroides_coprosuis
Otu267	58	Bacteria;Proteobacteria;Deltaproteobacteria;Desulfovibrionales;Desulfovibrionaceae;Desulfovibrio
Otu260	54	Bacteria;Firmicutes;Clostridia;Clostridiales;Clostridiaceae;Clostridium
Otu261	52	Bacteria;Cyanobacteria;4C0d-2;YS2
Otu262	96	Bacteria;Firmicutes;Bacilli;Lactobacillales;Enterococcaceae;Enterococcus
Otu308	39	Bacteria;Tenericutes;Mollicutes;RF39
Otu116	238	Bacteria;Bacteroidetes;Sphingobacteriia;Sphingobacteriales;Sphingobacteriaceae
Otu117	148	Bacteria;Firmicutes;Bacilli;Lactobacillales;Lactobacillaceae;Lactobacillus
Otu114	283	Bacteria;Bacteroidetes;Bacteroidia;Bacteroidales;Paraprevotellaceae

Otu115	232	Bacteria;Bacteroidetes;Cytophagia;Cytophagales;Cyclobacteriaceae
Otu112	167	Bacteria;Proteobacteria;Deltaproteobacteria;Bdellovibrionales;Bacteriovoracaceae
Otu113	488	Bacteria;Bacteroidetes;Bacteroidia;Bacteroidales;Porphyromonadaceae;Parabacteroides
Otu110	548	Bacteria;Bacteroidetes;Bacteroidia;Bacteroidales;Porphyromonadaceae
Otu111	153	Bacteria;Proteobacteria;Alphaproteobacteria;Rhizobiales;Brucellaceae
Otu118	183	Bacteria;Tenericutes;Mollicutes;Acholeplasmatales;Acholeplasmataceae
Otu119	149	Bacteria;Bacteroidetes;Bacteroidia;Bacteroidales;Bacteroidaceae;Bacteroides;Bacteroides_coprosuis
Otu286	52	Bacteria;Spirochaetes;Spirochaetes;Spirochaetales;Spirochaetaceae;Treponema
Otu287	39	Bacteria;Bacteroidetes;Flavobacteriia;Flavobacteriales;Flavobacteriaceae
Otu284	29	Bacteria;Firmicutes;Bacilli;Bacillales
Otu285	30	Bacteria;Bacteroidetes;Bacteroidia;Bacteroidales;p-2534-18B5
Otu282	61	Bacteria;Bacteroidetes;Sphingobacteriia;Sphingobacteriales;Sphingobacteriaceae
Otu283	66	Bacteria;Bacteroidetes;Bacteroidia;Bacteroidales;Prevotellaceae;Prevotella
Otu280	110	Bacteria;Firmicutes;Clostridia;Clostridiales
Otu281	69	Bacteria;Firmicutes;Erysipelotrichi;Erysipelotrichales;Erysipelotrichaceae;p-75-a5
Otu288	53	Bacteria;Firmicutes;Clostridia;Clostridiales;Ruminococcaceae
Otu289	38	Bacteria;Firmicutes;Bacilli;Lactobacillales;Aerococcaceae;Facklamia
Otu720	3	Bacteria;Bacteroidetes;Bacteroidia;Bacteroidales;Prevotellaceae
Otu756	5	Bacteria;Proteobacteria;Betaproteobacteria;Burkholderiales;Comamonadaceae;Limnohabitans
Otu990	7	Bacteria;Firmicutes;Clostridia;Clostridiales;Ruminococcaceae
Otu38	1204	Bacteria;Proteobacteria
Otu39	872	Bacteria;Proteobacteria;Gammaproteobacteria;Xanthomonadales;Xanthomonadaceae;Wohlfahrtiimonas
Otu36	2237	Bacteria;Proteobacteria;Gammaproteobacteria;Oceanospirillales;Halomonadaceae
Otu37	1043	Bacteria;Proteobacteria;Gammaproteobacteria;Alteromonadales;Idiomarinaceae;Pseudidiomarina
Otu34	1279	Bacteria;Firmicutes;Clostridia;Clostridiales;Lachnospiraceae;Roseburia
Otu35	2016	Bacteria;Firmicutes;Clostridia;Clostridiales;Veillonellaceae;Phascolarctobacterium

Otu32	1200	Bacteria;Bacteroidetes;Bacteroidia;Bacteroidales;Prevotellaceae;Prevotella
Otu33	2141	Bacteria;Bacteroidetes;Bacteroidia;Bacteroidales;Prevotellaceae;Prevotella
Otu30	997	Bacteria;Proteobacteria;Deltaproteobacteria;Desulfovibrionales;Desulfovibrionaceae;Desulfovibrio
Otu31	1497	Bacteria;Bacteroidetes;Bacteroidia;Bacteroidales
Otu163	243	Bacteria;Bacteroidetes;Bacteroidia;Bacteroidales
Otu162	157	Bacteria;Bacteroidetes;Flavobacteriia;Flavobacteriales;Weeksellaceae;Wautersiella
Otu161	447	Bacteria;Firmicutes;Clostridia;Clostridiales;Ruminococcaceae
Otu160	95	Bacteria;Bacteroidetes;Flavobacteriia;Flavobacteriales;Flavobacteriaceae;Myroides;Myroides_odoratimimus
Otu167	121	Bacteria;Firmicutes;Clostridia;Clostridiales;Clostridiaceae
Otu309	150	Bacteria;Firmicutes;Clostridia;Clostridiales
Otu165	115	Bacteria;Proteobacteria;Gammaproteobacteria;Alteromonadales;HTCC2188;HTCC
Otu164	244	Bacteria;Proteobacteria;Gammaproteobacteria;Xanthomonadales;Xanthomonadaceae;Stenotrophomonas
Otu169	88	Bacteria;Bacteroidetes;Flavobacteriia;Flavobacteriales;Cryomorphaceae;Brumimicrobium
Otu263	45	Bacteria;Firmicutes;Clostridia;Clostridiales
Otu1062	13	Bacteria;Bacteroidetes;Bacteroidia;Bacteroidales;Porphyromonadaceae
Otu801	4	Bacteria;Tenericutes;Mollicutes;RF39
Otu1160	4	Bacteria;Bacteroidetes;Bacteroidia;Bacteroidales
Otu1145	3	Bacteria;Firmicutes;Clostridia;Clostridiales;Ruminococcaceae
Otu1129	2	Bacteria;Firmicutes;Clostridia;Clostridiales
Otu998	2	Bacteria
Otu615	10	Bacteria;Firmicutes;Clostridia;Clostridiales;Lachnospiraceae;Blautia
Otu999	5	Bacteria;Firmicutes;Clostridia;Clostridiales;Ruminococcaceae
Otu929	235	Bacteria;Firmicutes;Clostridia;Clostridiales;Ruminococcaceae
Otu758	19	Bacteria;Bacteroidetes;Bacteroidia;Bacteroidales;Prevotellaceae;Prevotella
Otu841	9	Bacteria;Tenericutes;RF3;ML615J-28
Otu842	23	Bacteria;Bacteroidetes;Bacteroidia;Bacteroidales;Prevotellaceae;Prevotella

Otu843	9	Bacteria;Firmicutes
Otu844	2	Bacteria;Bacteroidetes;Sphingobacteriia;Sphingobacteriales;Sphingobacteriaceae;Sphingobacterium
Otu845	70	Bacteria;Firmicutes;Clostridia
Otu846	8	Bacteria;Firmicutes;Clostridia;Clostridiales
Otu847	3	Bacteria;Firmicutes;Erysipelotrichi;Erysipelotrichales;Erysipelotrichaceae
Otu848	4	Bacteria;Firmicutes;Clostridia;Clostridiales
Otu849	2	Bacteria;Bacteroidetes
Otu83	461	Bacteria;Firmicutes;Clostridia;Clostridiales;Ruminococcaceae;Ruminococcus;Ruminococcus_bromii
Otu82	623	Bacteria;Proteobacteria;Gammaproteobacteria;Pseudomonadales;Pseudomonadaceae
Otu81	229	Bacteria;Firmicutes;Clostridia;Clostridiales;Lachnospiraceae;Coprococcus
Otu80	505	Bacteria;Proteobacteria;Gammaproteobacteria;Pseudomonadales;Pseudomonadaceae
Otu87	220	Bacteria;Proteobacteria;Epsilonproteobacteria;Campylobacterales;Campylobacteraceae;Campylobacter
Otu86	113	Bacteria;Spirochaetes;Spirochaetes;Spirochaetales;Spirochaetaceae;Treponema
Otu85	225	Bacteria;Bacteroidetes;Saprospirae;Saprospirales;Chitinophagaceae
Otu84	439	Bacteria;Bacteroidetes;Bacteroidia;Bacteroidales;Paraprevotellaceae;Prevotella
Otu1103	2	Bacteria;Proteobacteria;Alphaproteobacteria;Rhizobiales;Phyllobacteriaceae
Otu89	520	Bacteria;Bacteroidetes;Bacteroidia;Bacteroidales;Prevotellaceae;Prevotella
Otu88	190	Bacteria;Proteobacteria;Alphaproteobacteria;Caulobacterales;Caulobacteraceae;Brevundimonas;Brevundimonas_diminuta
Otu928	6	Bacteria;Proteobacteria;Gammaproteobacteria;Xanthomonadales;Xanthomonadaceae;Luteimonas
Otu759	8	Bacteria;Firmicutes;Clostridia;Clostridiales;Lachnospiraceae;Epulopiscium
Otu1102	2	Bacteria;Cyanobacteria;4C0d-2;YS2
Otu1101	2	Bacteria;Firmicutes;Clostridia;Clostridiales
Otu1149	2	Bacteria;Actinobacteria;Actinobacteria;Actinomycetales;Corynebacteriaceae;Corynebacterium
Otu1100	8	Bacteria;Firmicutes;Clostridia;Clostridiales;Lachnospiraceae;Blautia;Blautia_obeum
Otu1128	3	Bacteria;Firmicutes;Clostridia;Clostridiales;Ruminococcaceae
Otu1011	20	Bacteria;Bacteroidetes;Bacteroidia;Bacteroidales;Porphyromonadaceae;Dysgonomonas

Otu426	31	Bacteria;Bacteroidetes;Bacteroidia;Bacteroidales;S24-7
Otu617	209	Bacteria;Bacteroidetes;Bacteroidia;Bacteroidales;Prevotellaceae;Prevotella;Prevotella_stercorea
Otu427	59	Bacteria;Bacteroidetes;Bacteroidia;Bacteroidales;Prevotellaceae;Prevotella
Otu798	9	Bacteria;Firmicutes;Clostridia;Clostridiales;Dehalobacteriaceae;Dehalobacterium
Otu976	4	Bacteria;Firmicutes;Clostridia;Clostridiales;Ruminococcaceae
Otu424	27	Bacteria;Firmicutes;Clostridia;Clostridiales;Lachnospiraceae;Coprococcus
Otu799	8	Bacteria;Verrucomicrobia;Verruco-5;WCHB1-41;RFP12
Otu501	61	Bacteria;Firmicutes;Clostridia;Clostridiales;Lachnospiraceae
Otu500	14	Bacteria;Firmicutes;Clostridia;Clostridiales;Ruminococcaceae;Oscillospira
Otu503	22	Bacteria;Bacteroidetes;Bacteroidia;Bacteroidales
Otu425	20	Bacteria;Actinobacteria;Coriobacteriia;Coriobacteriales;Coriobacteriaceae
Otu505	21	Bacteria;Tenericutes;Mollicutes;Acholeplasmatales;Acholeplasmataceae;Acholeplasma
Otu504	10	Bacteria;Firmicutes;Erysipelotrichi;Erysipelotrichales;Erysipelotrichaceae
Otu507	6	Bacteria;Firmicutes;Clostridia;Clostridiales
Otu506	12	Bacteria;Firmicutes;Clostridia;Clostridiales;Ruminococcaceae
Otu509	11	Bacteria;Actinobacteria;Coriobacteriia;Coriobacteriales;Coriobacteriaceae
Otu422	11	Bacteria;Bacteroidetes;Flavobacteriia;Flavobacteriales;Flavobacteriaceae;Myroides
Otu1010	12	Bacteria;Firmicutes;Clostridia;Clostridiales;Lachnospiraceae;Coprococcus
Otu423	10	Bacteria;Firmicutes;Clostridia;Clostridiales;Lachnospiraceae
Otu1068	5	Bacteria;Bacteroidetes;Flavobacteriia;Flavobacteriales;Weeksellaceae;Chryseobacterium
Otu1069	11	Bacteria;Proteobacteria;Gammaproteobacteria
Otu420	18	Bacteria;Verrucomicrobia;Verruco-5;WCHB1-41;RFP12
Otu1060	2	Bacteria;Bacteroidetes
Otu1061	2	Bacteria;Bacteroidetes;Bacteroidia;Bacteroidales;Porphyromonadaceae;Dysgonomonas
Otu977	7	Bacteria;Bacteroidetes;Bacteroidia;Bacteroidales;S24-7
Otu421	35	Bacteria;Bacteroidetes;Bacteroidia;Bacteroidales;Paraprevotellaceae;Prevotella

Otu1064	33	Bacteria;Bacteroidetes;Bacteroidia;Bacteroidales;Prevotellaceae;Prevotella;Prevotella_copri
Otu1065	2	Bacteria;Proteobacteria;Gammaproteobacteria;Alteromonadales;Alteromonadaceae;Cellvibrio
Otu1066	2	Bacteria;Firmicutes;Clostridia;Clostridiales;Ruminococcaceae
Otu1067	2	Bacteria;Tenericutes;Mollicutes;RF39
Otu480	44	Bacteria;Firmicutes;Clostridia;Clostridiales;Ruminococcaceae
Otu481	16	Bacteria;Firmicutes;Clostridia;Clostridiales;Lachnospiraceae;Lachnospira
Otu482	53	Bacteria;Firmicutes;Clostridia;Clostridiales;Lachnospiraceae
Otu483	13	Bacteria
Otu484	16	Bacteria;Bacteroidetes;Bacteroidia;Bacteroidales;Paraprevotellaceae;Prevotella
Otu485	10	Bacteria;Firmicutes;Bacilli;Lactobacillales;Aerococcaceae
Otu338	45	Bacteria;Bacteroidetes;Bacteroidia;Bacteroidales;Bacteroidaceae;Bacteroides
Otu339	32	Bacteria;Firmicutes;Clostridia;Clostridiales;Lachnospiraceae
Otu336	30	Bacteria;Firmicutes;Bacilli;Lactobacillales;Aerococcaceae;Facklamia
Otu337	23	Bacteria;Firmicutes;Erysipelotrichi;Erysipelotrichales;Erysipelotrichaceae;Eubacterium
Otu334	36	Bacteria;Firmicutes;Clostridia;Clostridiales;Ruminococcaceae
Otu335	32	Bacteria;Firmicutes;Clostridia;Clostridiales;Lachnospiraceae
Otu332	53	Bacteria;Proteobacteria;Deltaproteobacteria;GMD14H09
Otu333	37	Bacteria;Proteobacteria;Deltaproteobacteria;Desulfovibrionales;Desulfovibrionaceae
Otu330	71	Bacteria;Firmicutes;Clostridia;Clostridiales;Ruminococcaceae;Ruminococcus
Otu331	90	Bacteria;Firmicutes;Bacilli;Lactobacillales;Carnobacteriaceae;Trichococcus
Otu645	24	Bacteria;Firmicutes;Bacilli;Lactobacillales;Leuconostocaceae
Otu792	2	Bacteria;Firmicutes;Clostridia;Clostridiales;Ruminococcaceae
Otu970	2	Bacteria;Firmicutes;Clostridia;Clostridiales
Otu793	11	Bacteria;Firmicutes;Clostridia;Clostridiales;Ruminococcaceae
Otu880	5	Bacteria;Proteobacteria
Otu1037	2	Bacteria;Firmicutes;Clostridia;Clostridiales;Ruminococcaceae;Oscillospira

Otu657	8	Bacteria;Firmicutes;Clostridia;Clostridiales;Mogibacteriaceae;Mogibacterium
Otu640	18	Bacteria;Firmicutes;Clostridia;Clostridiales;Tissierellaceae
Otu641	25	Bacteria;Proteobacteria;Deltaproteobacteria;Desulfovibrionales;Desulfovibrionaceae;Desulfovibrio
Otu1114	5	Bacteria;Firmicutes;Erysipelotrichi;Erysipelotrichales;Erysipelotrichaceae;RFN20
Otu1115	2	Bacteria;Bacteroidetes;Bacteroidia;Bacteroidales;Prevotellaceae;Prevotella
Otu349	25	Bacteria;Firmicutes;Clostridia;Clostridiales;Ruminococcaceae
Otu348	18	Bacteria;Spirochaetes;Spirochaetes;Spirochaetales;Spirochaetaceae;Treponema
Otu439	20	Bacteria;Firmicutes;Clostridia;Clostridiales;Ruminococcaceae;Ruminococcus
Otu438	20	Bacteria;Firmicutes;Clostridia;Clostridiales;Tissierellaceae;Sedimentibacter
Otu971	2	Bacteria;Bacteroidetes;Bacteroidia;Bacteroidales
Otu1113	8	Bacteria;Firmicutes;Clostridia;Clostridiales;Ruminococcaceae;Faecalibacterium;Faecalibacterium_prausnitzii
Otu343	36	Bacteria;Bacteroidetes;Bacteroidia;Bacteroidales;Paraprevotellaceae
Otu342	36	Bacteria;Bacteroidetes;Bacteroidia;Bacteroidales;Prevotellaceae;Prevotella
Otu341	107	Bacteria;Spirochaetes;Spirochaetes;Spirochaetales;Spirochaetaceae;Treponema
Otu340	67	Bacteria;Proteobacteria;Betaproteobacteria;Burkholderiales;Alcaligenaceae;Oligella
Otu347	29	Bacteria;Firmicutes;Clostridia;Clostridiales
Otu346	54	Bacteria;Firmicutes;Clostridia;Clostridiales;Lachnospiraceae
Otu345	46	Bacteria;Bacteroidetes;Bacteroidia;Bacteroidales;Prevotellaceae;Prevotella
Otu344	62	Bacteria;Firmicutes;Clostridia;Clostridiales;Ruminococcaceae
Otu655	14	Bacteria;Firmicutes;Clostridia;Clostridiales
Otu654	4	Bacteria;Tenericutes;Mollicutes;RF39
Otu158	173	Bacteria;Bacteroidetes;Bacteroidia;Bacteroidales;Bacteroidaceae;Bacteroides
Otu159	167	Bacteria;Firmicutes;Clostridia;Clostridiales;Ruminococcaceae
Otu651	24	Bacteria;Bacteroidetes;Bacteroidia;Bacteroidales
Otu650	53	Bacteria;Firmicutes;Clostridia;Clostridiales;Lachnospiraceae
Otu653	15	Bacteria;Firmicutes;Clostridia;Clostridiales;Lachnospiraceae

Otu652	8	Bacteria;Firmicutes;Clostridia;Clostridiales;Lachnospiraceae;Coprococcus
Otu152	173	Bacteria;Proteobacteria;Gammaproteobacteria
Otu153	135	Bacteria;Bacteroidetes;Bacteroidia;Bacteroidales;Prevotellaceae;Prevotella
Otu150	539	Bacteria;Bacteroidetes;Bacteroidia;Bacteroidales
Otu151	77	Bacteria;Proteobacteria;Alphaproteobacteria;Rhodobacterales;Rhodobacteraceae;Paracoccus
Otu156	195	Bacteria;Firmicutes;Bacilli;Lactobacillales;Lactobacillaceae;Lactobacillus
Otu157	188	Bacteria;Bacteroidetes;Bacteroidia;Bacteroidales;S24-7
Otu154	610	Bacteria;Bacteroidetes;Bacteroidia;Bacteroidales;Paraprevotellaceae;Prevotella
Otu155	98	Bacteria
Otu72	376	Bacteria;Firmicutes;Clostridia;Clostridiales;Clostridiaceae;SMB53
Otu73	568	Bacteria;Firmicutes;Clostridia;Clostridiales;Ruminococcaceae;Oscillospira
Otu70	1399	Bacteria;Firmicutes;Bacilli;Lactobacillales;Lactobacillaceae;Lactobacillus;Lactobacillus_reuteri
Otu71	356	Bacteria;Proteobacteria;Gammaproteobacteria;Aeromonadales;Succinivibrionaceae
Otu76	295	Bacteria;Bacteroidetes;Bacteroidia;Bacteroidales;Prevotellaceae;Prevotella
Otu77	716	Bacteria;Firmicutes;Clostridia;Clostridiales;Ruminococcaceae;Oscillospira
Otu74	318	Bacteria;Bacteroidetes;Bacteroidia;Bacteroidales;Porphyromonadaceae
Otu75	516	Bacteria;Proteobacteria;Gammaproteobacteria
Otu930	2	Bacteria;Tenericutes;Mollicutes;Acholeplasmatales;Acholeplasmataceae
Otu563	12	Bacteria;Firmicutes;Clostridia;Clostridiales;Ruminococcaceae
Otu78	288	Bacteria;Tenericutes;Mollicutes;Acholeplasmatales;Acholeplasmataceae;Acholeplasma
Otu79	344	Bacteria;Bacteroidetes;Bacteroidia;Bacteroidales;S24-7
Otu934	7	Bacteria;Firmicutes;Clostridia;Clostridiales;Ruminococcaceae
Otu935	9	Bacteria;Firmicutes;Clostridia;Clostridiales;Tissierellaceae;ph2
Otu936	2	Bacteria;Firmicutes;Clostridia;Clostridiales
Otu562	14	Bacteria;Firmicutes;Clostridia;Clostridiales;Lachnospiraceae;Coprococcus
Otu1135	4	Bacteria;Proteobacteria;Betaproteobacteria;Burkholderiales;Alcaligenaceae;Sutterella

Otu1132	45	Bacteria;Proteobacteria;Gammaproteobacteria;Alteromonadales;Alteromonadaceae;BD2-13
Otu959	11	Bacteria
Otu1162	2	Bacteria;Bacteroidetes;Bacteroidia;Bacteroidales
Otu915	2	Bacteria;Synergistetes;Synergistia;Synergistales;Synergistaceae
Otu1133	5	Bacteria;Firmicutes;Clostridia;Clostridiales;Ruminococcaceae
Otu237	128	Bacteria;Cyanobacteria;4C0d-2;YS2
Otu236	110	Bacteria;Bacteroidetes;Bacteroidia;Bacteroidales;Prevotellaceae
Otu235	44	Bacteria;Firmicutes;Bacilli;Lactobacillales;Enterococcaceae;Vagococcus
Otu234	70	Bacteria;Tenericutes;Mollicutes;Acholeplasmatales;Acholeplasmataceae;Acholeplasma
Otu233	234	Bacteria;Bacteroidetes;Bacteroidia;Bacteroidales;Prevotellaceae;Prevotella
Otu232	80	Bacteria;Spirochaetes;Spirochaetes;Spirochaetales;Spirochaetaceae;Treponema
Otu231	65	Bacteria;Firmicutes;Clostridia;Clostridiales;Lachnospiraceae
Otu230	54	Bacteria;Firmicutes;Clostridia;Clostridiales;Tissierellaceae
Otu909	2	Bacteria;Firmicutes;Clostridia;Clostridiales;Lachnospiraceae
Otu1038	2	Bacteria;Firmicutes;Clostridia;Clostridiales;Ruminococcaceae
Otu378	32	Bacteria;Proteobacteria;Deltaproteobacteria;Desulfovibrionales;Desulfovibrionaceae
Otu239	93	Bacteria;Firmicutes;Clostridia;Clostridiales;Ruminococcaceae
Otu238	188	Bacteria;Bacteroidetes;Flavobacteriia;Flavobacteriales;Weeksellaceae
Otu952	3	Bacteria;Firmicutes;Clostridia;Clostridiales;Ruminococcaceae
Otu953	5	Bacteria;Firmicutes;Clostridia;Clostridiales;Ruminococcaceae
Otu974	9	Bacteria;Tenericutes;Mollicutes;RF39
Otu899	2	Bacteria
Otu804	90	Bacteria;Firmicutes;Clostridia;Clostridiales;Ruminococcaceae
Otu805	2	Bacteria;Firmicutes;Clostridia;Clostridiales;Lachnospiraceae
Otu806	7	Bacteria;Firmicutes;Erysipelotrichi;Erysipelotrichales;Erysipelotrichaceae;Bulleidia
Otu807	3	Bacteria

Otu800	2	Bacteria;Bacteroidetes;Bacteroidia;Bacteroidales;Prevotellaceae;Prevotella
Otu569	17	Bacteria;Firmicutes;Clostridia;Clostridiales;Lachnospiraceae;Coprococcus
Otu802	2	Bacteria;Firmicutes;Clostridia;Clostridiales
Otu803	114	Bacteria;Firmicutes;Clostridia;Clostridiales;Ruminococcaceae
Otu1151	2	Bacteria;Tenericutes;Mollicutes;Acholeplasmatales;Acholeplasmataceae
Otu956	4	Bacteria;Tenericutes;Mollicutes;Acholeplasmatales;Acholeplasmataceae
Otu568	9	Bacteria;Spirochaetes;Spirochaetes;Spirochaetales;Spirochaetaceae;Treponema
Otu808	7	Bacteria;Firmicutes;Clostridia;Clostridiales
Otu809	13	Bacteria;Firmicutes;Clostridia;Clostridiales;Tissierellaceae
Otu957	2	Bacteria;Firmicutes;Clostridia;Clostridiales
Otu1016	41	Bacteria
Otu770	3	Bacteria;Firmicutes;Clostridia;Clostridiales;Ruminococcaceae;Oscillospira
Otu913	23	Bacteria;Cyanobacteria;4C0d-2;YS2
Otu771	120	Bacteria;Proteobacteria;Gammaproteobacteria;Pseudomonadales;Pseudomonadaceae
Otu612	11	Bacteria;Firmicutes;Clostridia;Clostridiales;Veillonellaceae;Dialister
Otu893	2	Bacteria;Bacteroidetes;Bacteroidia;Bacteroidales;Prevotellaceae;Prevotella
Otu881	2	Bacteria
Otu810	4	Bacteria;Proteobacteria;Betaproteobacteria;Burkholderiales
Otu892	8	Bacteria;Firmicutes;Clostridia;Clostridiales
Otu891	2	Bacteria;Bacteroidetes;Bacteroidia;Bacteroidales;Prevotellaceae;Prevotella
Otu1108	2	Bacteria;Firmicutes
Otu166	54	Bacteria;Bacteroidetes;Bacteroidia;Bacteroidales;Porphyromonadaceae;Paludibacter
Otu890	2	Bacteria;Tenericutes;Mollicutes;Acholeplasmatales;Acholeplasmataceae;Acholeplasma
Otu897	5	Bacteria;Firmicutes;Clostridia;Clostridiales;Ruminococcaceae
Otu1009	20	Bacteria;Spirochaetes;Spirochaetes;Spirochaetales;Spirochaetaceae;Treponema
Otu896	2	Bacteria;Bacteroidetes;Bacteroidia;Bacteroidales

Otu1155	9	Bacteria;Firmicutes;Clostridia;Clostridiales;Lachnospiraceae
Otu961	3	Bacteria;Firmicutes;Clostridia;Clostridiales;Tissierellaceae;Gallicola
Otu778	2	Bacteria;Firmicutes
Otu1051	7	Bacteria;Proteobacteria;Alphaproteobacteria;RF32
Otu779	381	Bacteria;Firmicutes;Clostridia;Clostridiales;Ruminococcaceae
Otu1050	3	Bacteria;Firmicutes;Clostridia;Clostridiales
Otu168	73	Bacteria;Proteobacteria
Otu724	28	Bacteria;Bacteroidetes;Bacteroidia;Bacteroidales;Porphyromonadaceae;Dysgonomonas
Otu1138	8	Bacteria;Tenericutes;RF3;ML615J-28
Otu948	7	Bacteria;Firmicutes
Otu8	3600	Bacteria;Bacteroidetes;Bacteroidia;Bacteroidales;Prevotellaceae;Prevotella
Otu9	4538	Bacteria;Bacteroidetes;Flavobacteriia;Flavobacteriales;Weeksellaceae
Otu6	6407	Bacteria;Proteobacteria;Gammaproteobacteria;Pseudomonadales;Moraxellaceae;Acinetobacter
Otu7	3334	Bacteria;Proteobacteria;Betaproteobacteria;Burkholderiales;Comamonadaceae;Comamonas
Otu4	7206	Bacteria;Firmicutes;Bacilli;Lactobacillales;Lactobacillaceae;Lactobacillus
Otu5	4873	Bacteria;Proteobacteria;Gammaproteobacteria;Pseudomonadales;Pseudomonadaceae
Otu2	11643	Bacteria;Proteobacteria;Gammaproteobacteria;Pseudomonadales;Pseudomonadaceae;Pseudomonas
Otu3	4748	Bacteria;Bacteroidetes;Bacteroidia;Bacteroidales;Bacteroidaceae;Bacteroides;Bacteroides_coprosuis
Otu1	10973	Bacteria;Spirochaetes;Spirochaetes;Spirochaetales;Spirochaetaceae;Treponema
Otu387	29	Bacteria;Firmicutes;Clostridia;Clostridiales;Lachnospiraceae;Coprococcus
Otu386	28	Bacteria;Firmicutes;Clostridia;Clostridiales;Clostridiaceae;Clostridium
Otu385	21	Bacteria;Firmicutes;Clostridia;Clostridiales;Peptococcaceae;Niigata-25
Otu941	4	Bacteria;Firmicutes;Clostridia;Clostridiales;Ruminococcaceae;Ruminococcus
Otu471	26	Bacteria;Tenericutes;Mollicutes;Acholeplasmatales;Acholeplasmataceae;Acholeplasma
Otu470	196	Bacteria;Firmicutes;Bacilli;Bacillales;Planococcaceae;Lysinibacillus
Otu473	25	Bacteria;Proteobacteria;Alphaproteobacteria;Rhodobacterales;Rhodobacteraceae;Rhodobacter

Otu384	21	Bacteria;Proteobacteria;Gammaproteobacteria
Otu475	25	Bacteria;Proteobacteria;Gammaproteobacteria;Enterobacteriales;Enterobacteriaceae;Proteus
Otu474	6	Bacteria;Firmicutes;Clostridia;Clostridiales;Mogibacteriaceae
Otu477	17	Bacteria;Spirochaetes;Spirochaetes;Sphaerochaetales;Sphaerochaetaceae;Sphaerochaeta
Otu476	22	Bacteria;Bacteroidetes;Bacteroidia;Bacteroidales
Otu479	16	Bacteria;Firmicutes;Clostridia;Clostridiales;Lachnospiraceae
Otu383	14	Bacteria;Bacteroidetes;Bacteroidia;Bacteroidales;Porphyromonadaceae;Dysgonomonas
Otu382	35	Bacteria;Firmicutes;Erysipelotrichi;Erysipelotrichales;Erysipelotrichaceae;Eubacterium;Eubacterium_biforme
Otu578	18	Bacteria;Bacteroidetes;Bacteroidia;Bacteroidales;Rikenellaceae
Otu579	1821	Bacteria;Bacteroidetes;Bacteroidia;Bacteroidales;Prevotellaceae;Prevotella
Otu1013	6	Bacteria;Firmicutes;Clostridia;Clostridiales;Ruminococcaceae;Oscillospira
Otu1012	8	Bacteria;Firmicutes;Clostridia;Clostridiales
Otu648	10	Bacteria;Firmicutes;Clostridia;Clostridiales;Ruminococcaceae;Butyricicoccus;Butyricicoccus_pullicaecorum
Otu381	31	Bacteria;Firmicutes;Clostridia;Clostridiales;Ruminococcaceae
Otu1017	10	Bacteria;Firmicutes;Clostridia;Clostridiales;Lachnospiraceae
Otu746	2	Bacteria;Firmicutes;Clostridia;Clostridiales;Veillonellaceae;Phascolarctobacterium
Otu570	23	Bacteria;Firmicutes;Clostridia;Clostridiales;Lachnospiraceae;Blautia;Blautia_producta
Otu571	7	Bacteria
Otu572	16	Bacteria;Firmicutes;Clostridia;Clostridiales;Ruminococcaceae
Otu380	40	Bacteria;Firmicutes;Clostridia;Clostridiales;Clostridiaceae;Clostridium
Otu574	15	Bacteria;Firmicutes;Clostridia;Clostridiales;Lachnospiraceae
Otu575	24	Bacteria;Firmicutes;Clostridia;Clostridiales
Otu576	4	Bacteria;Proteobacteria;Alphaproteobacteria;Rhizobiales;Rhizobiaceae;Agrobacterium
Otu577	5	Bacteria;Firmicutes;Clostridia;Clostridiales;Peptococcaceae
Otu619	2249	Bacteria;Bacteroidetes;Bacteroidia;Bacteroidales;Porphyromonadaceae
Otu618	9	Bacteria;Verrucomicrobia;Verruco-5;WCHB1-41;RFP12

Otu752	5	Bacteria;Firmicutes;Clostridia;Clostridiales
Otu744	2	Bacteria;Firmicutes
Otu754	16	Bacteria;Firmicutes;Clostridia;Clostridiales;Mogibacteriaceae
Otu755	17	Bacteria;Firmicutes;Clostridia;Clostridiales
Otu208	72	Bacteria;Bacteroidetes;Bacteroidia;Bacteroidales;Prevotellaceae;Prevotella
Otu209	81	Bacteria;Firmicutes;Clostridia;Clostridiales;Ruminococcaceae
Otu206	153	Bacteria;Firmicutes;Clostridia;Clostridiales;Ruminococcaceae;Faecalibacterium;Faecalibacterium_prausnitzii
Otu207	83	Bacteria;Firmicutes;Bacilli;Lactobacillales;Aerococcaceae;Aerococcus
Otu204	70	Bacteria;Bacteroidetes;Bacteroidia;Bacteroidales;S24-7
Otu205	45	Bacteria;Cyanobacteria;4C0d-2;YS2
Otu202	99	Bacteria;Firmicutes;Clostridia;Clostridiales;Ruminococcaceae
Otu203	113	Bacteria;Firmicutes;Clostridia;Clostridiales;Lachnospiraceae
Otu200	76	Bacteria;Bacteroidetes;Bacteroidia;Bacteroidales;Marinilabiaceae
Otu201	67	Bacteria;Actinobacteria;Coriobacteriia;Coriobacteriales;Coriobacteriaceae
Otu659	4	Bacteria
Otu741	6	Bacteria;Proteobacteria;Alphaproteobacteria;Rhizobiales;Hyphomicrobiaceae;Devosia
Otu767	2	Bacteria;Bacteroidetes;Bacteroidia;Bacteroidales
Otu740	28	Bacteria;Firmicutes;Clostridia;Clostridiales;Ruminococcaceae
Otu620	9	Bacteria;Bacteroidetes;Bacteroidia;Bacteroidales;p-2534-18B5
Otu682	16	Bacteria;Bacteroidetes;Bacteroidia;Bacteroidales;Porphyromonadaceae
Otu683	3	Bacteria;Bacteroidetes;Sphingobacteriia;Sphingobacteriales;Sphingobacteriaceae;Sphingobacterium
Otu680	6	Bacteria
Otu681	44	Bacteria;Proteobacteria;Betaproteobacteria;Burkholderiales;Comamonadaceae;Hylemonella
Otu686	3	Bacteria;Thermi;Deinococci;Deinococcales;Trueperaceae
Otu687	3	Bacteria;Firmicutes;Clostridia;Clostridiales;Lachnospiraceae
Otu684	11	Bacteria;Firmicutes;Clostridia;Clostridiales;Ruminococcaceae;Ruminococcus

Otu685	7	Bacteria;Bacteroidetes;Bacteroidia;Bacteroidales;Prevotellaceae;Prevotella
Otu688	26	Bacteria;Bacteroidetes;Bacteroidia;Bacteroidales;Bacteroidaceae;Bacteroides;Bacteroides_ovatus
Otu689	4	Bacteria;Firmicutes;Clostridia;Clostridiales;Ruminococcaceae
Otu658	6	Bacteria;Elusimicrobia;Elusimicrobia;Elusimicrobiales;Elusimicrobiaceae
Otu372	25	Bacteria;Firmicutes;Clostridia;Clostridiales;Peptococcaceae;Peptococcus
Otu373	15	Bacteria;Firmicutes;Clostridia;Clostridiales;Ruminococcaceae;Butyricicoccus;Butyricicoccus_pullicaecorum
Otu279	38	Bacteria;Proteobacteria;Alphaproteobacteria;Rhizobiales;Hyphomicrobiaceae;Devosia
Otu278	82	Bacteria;Bacteroidetes;Bacteroidia;Bacteroidales
Otu376	34	Bacteria;Firmicutes;Clostridia;Clostridiales;Ruminococcaceae;Ruminococcus
Otu377	58	Bacteria;Firmicutes;Clostridia;Clostridiales;Veillonellaceae
Otu374	28	Bacteria;Bacteroidetes;Bacteroidia;Bacteroidales;p-2534-18B5
Otu375	29	Bacteria;Firmicutes;Clostridia;Clostridiales;Ruminococcaceae
Otu273	54	Bacteria;Firmicutes;Clostridia;Clostridiales;Veillonellaceae;Dialister
Otu272	69	Bacteria;Firmicutes;Clostridia;Clostridiales;Clostridiaceae;Alkaliphilus
Otu271	59	Bacteria;Bacteroidetes;Bacteroidia;Bacteroidales;Paraprevotellaceae;Prevotella
Otu270	63	Bacteria;Firmicutes;Clostridia;Clostridiales
Otu277	81	Bacteria;Firmicutes;Clostridia;Clostridiales;Ruminococcaceae;Faecalibacterium;Faecalibacterium_prausnitzii
Otu276	65	Bacteria
Otu275	107	Bacteria;Proteobacteria;Betaproteobacteria;Burkholderiales;Alcaligenaceae
Otu274	32	Bacteria;Tenericutes;RF3;ML615J-28
Otu105	230	Bacteria;Bacteroidetes;Bacteroidia;Bacteroidales;RF16
Otu104	229	Bacteria;Firmicutes;Clostridia;Clostridiales;Christensenellaceae
Otu107	327	Bacteria;Bacteroidetes;Bacteroidia;Bacteroidales;Prevotellaceae;Prevotella
Otu106	185	Bacteria;Bacteroidetes
Otu101	284	Bacteria;Spirochaetes;Spirochaetes;Spirochaetales;Spirochaetaceae;Treponema
Otu100	1186	Bacteria;Firmicutes;Clostridia;Clostridiales;Ruminococcaceae

Otu103	139	Bacteria;Bacteroidetes
Otu102	191	Bacteria;Proteobacteria;Gammaproteobacteria
Otu894	5	Bacteria;Firmicutes;Clostridia;Clostridiales;Lachnospiraceae
Otu109	165	Bacteria;Proteobacteria;Alphaproteobacteria;Rhizobiales;Phyllobacteriaceae
Otu108	643	Bacteria;Bacteroidetes;Bacteroidia;Bacteroidales
Otu940	8	Bacteria;Firmicutes;Clostridia;Clostridiales;Lachnospiraceae
Otu295	52	Bacteria;Firmicutes;Clostridia;Clostridiales;Clostridiaceae
Otu294	99	Bacteria;Bacteroidetes;Bacteroidia;Bacteroidales;Paraprevotellaceae;CF231
Otu297	30	Bacteria;Proteobacteria;Alphaproteobacteria;Rhodobacterales;Rhodobacteraceae
Otu296	49	Bacteria;Firmicutes;Clostridia;Clostridiales
Otu291	18	Bacteria;Bacteroidetes;Bacteroidia;Bacteroidales;Porphyromonadaceae;Dysgonomonas
Otu290	30	Bacteria;Bacteroidetes;Bacteroidia;Bacteroidales;Paraprevotellaceae
Otu293	51	Bacteria;Proteobacteria;Betaproteobacteria;Rhodocyclales;Rhodocyclaceae
Otu292	36	Bacteria;Proteobacteria;Alphaproteobacteria;Sphingomonadales
Otu299	45	Bacteria;Bacteroidetes;Bacteroidia;Bacteroidales;Paraprevotellaceae
Otu298	65	Bacteria;Bacteroidetes;Bacteroidia;Bacteroidales;Prevotellaceae;Prevotella
Otu703	7	Bacteria;Firmicutes;Clostridia;Clostridiales;Dehalobacteriaceae
Otu702	4	Bacteria;Bacteroidetes
Otu701	9	Bacteria;Firmicutes;Clostridia;Clostridiales;Ruminococcaceae;Ruminococcus
Otu497	14	Bacteria;Proteobacteria;Gammaproteobacteria;Alteromonadales;Alteromonadaceae
Otu700	10	Bacteria;Verrucomicrobia;Verruco-5;WCHB1-41;WCHB1-25
Otu944	7	Bacteria;Bacteroidetes;Bacteroidia;Bacteroidales;Porphyromonadaceae;Paludibacter
Otu198	115	Bacteria;Firmicutes;Erysipelotrichi;Erysipelotrichales;Erysipelotrichaceae;Bulleidia;Bulleidia_p-1630-c5
Otu199	260	Bacteria;Bacteroidetes;Bacteroidia;Bacteroidales;Prevotellaceae;Prevotella
Otu196	160	Bacteria;Firmicutes;Clostridia;Clostridiales;Ruminococcaceae
Otu197	419	Bacteria;Bacteroidetes;Bacteroidia;Bacteroidales;Porphyromonadaceae;Parabacteroides

Otu194	68	Bacteria;Bacteroidetes;Bacteroidia;Bacteroidales;Prevotellaceae;Prevotella
Otu195	95	Bacteria;Planctomycetes;Planctomycetia;Pirellulales;Pirellulaceae
Otu192	75	Bacteria;Proteobacteria;Gammaproteobacteria;Pseudomonadales;Moraxellaceae;Psychrobacter
Otu193	95	Bacteria;Bacteroidetes;Bacteroidia;Bacteroidales;Prevotellaceae;Prevotella
Otu190	66	Bacteria;Bacteroidetes;Flavobacteriia;Flavobacteriales;Cryomorphaceae
Otu191	122	Bacteria;Bacteroidetes;Bacteroidia;Bacteroidales;S24-7
Otu840	9	Bacteria;Firmicutes;Clostridia;Clostridiales;Ruminococcaceae
Otu494	9	Bacteria;Fibrobacteres;Fibrobacteria;Fibrobacterales;Fibrobacteraceae;Fibrobacter
Otu705	4	Bacteria;Firmicutes;Erysipelotrichi;Erysipelotrichales;Erysipelotrichaceae
Otu493	25	Bacteria;Firmicutes;Clostridia;Clostridiales;Ruminococcaceae;Oscillospira
Otu704	3	Bacteria;Firmicutes;Bacilli;Bacillales;Planococcaceae
Otu743	2	Bacteria;Firmicutes;Clostridia;Clostridiales;Lachnospiraceae
Otu492	10	Bacteria;Tenericutes;Mollicutes;RF39
Otu491	14	Bacteria;Tenericutes;Mollicutes;RF39
Otu490	12	Bacteria;Proteobacteria;Alphaproteobacteria;Rhizobiales;Hyphomicrobiaceae;Devosia
Otu996	8	Bacteria;Bacteroidetes;Bacteroidia;Bacteroidales;Porphyromonadaceae;Dysgonomonas
Otu997	3	Bacteria;Firmicutes
Otu994	2	Bacteria;Firmicutes;Clostridia;Clostridiales
Otu995	12	Bacteria;Firmicutes;Clostridia;Clostridiales;Lachnospiraceae
Otu992	4	Bacteria;Cyanobacteria;4C0d-2;YS2
Otu993	7	Bacteria;Proteobacteria;Betaproteobacteria;Burkholderiales;Alcaligenaceae;Sutterella
Otu859	2	Bacteria;Proteobacteria;Gammaproteobacteria
Otu858	6	Bacteria;Firmicutes;Clostridia;Clostridiales
Otu857	2	Bacteria;Proteobacteria;Alphaproteobacteria;Rhizobiales
Otu856	3	Bacteria;Tenericutes;Mollicutes;RF39
Otu855	3	Bacteria;Proteobacteria;Gammaproteobacteria;Pseudomonadales;Pseudomonadaceae

Otu854	5	Bacteria;Actinobacteria;Actinobacteria;Bifidobacteriales;Bifidobacteriaceae;Bifidobacterium
Otu853	5	Bacteria;Firmicutes;Clostridia;Clostridiales
Otu852	2	Bacteria;Bacteroidetes;Cytophagia;Cytophagales
Otu851	16	Bacteria;Cyanobacteria;4C0d-2;YS2
Otu850	2	Bacteria;Firmicutes;Clostridia;Clostridiales;Ruminococcaceae
Otu98	304	Bacteria;Firmicutes;Clostridia;Clostridiales;Tissierellaceae
Otu99	196	Bacteria;Proteobacteria;Betaproteobacteria;Burkholderiales;Oxalobacteraceae;Oxalobacter
Otu960	7	Bacteria;Firmicutes;Clostridia;Clostridiales;Ruminococcaceae
Otu94	167	Bacteria;Proteobacteria;Alphaproteobacteria;Sphingomonadales;Sphingomonadaceae
Otu95	860	Bacteria;Firmicutes;Clostridia;Clostridiales;Ruminococcaceae
Otu96	352	Bacteria;Firmicutes;Clostridia;Clostridiales;Tissierellaceae;Anaerococcus
Otu97	173	Bacteria;Bacteroidetes
Otu90	298	Bacteria;Firmicutes;Clostridia;Clostridiales;Ruminococcaceae
Otu91	204	Bacteria;Proteobacteria;Epsilonproteobacteria;Campylobacterales;Campylobacteraceae;Arcobacter;Arcobacter_cryaerophilus
Otu92	136	Bacteria;Bacteroidetes;Flavobacteriia;Flavobacteriales;Flavobacteriaceae;Myroides
Otu93	386	Bacteria;Bacteroidetes;Bacteroidia;Bacteroidales;S24-7
Otu984	6	Archaea;Euryarchaeota;Methanobacteria;Methanobacteriales;Methanobacteriaceae;Methanobrevibacter
Otu949	4	Bacteria;Firmicutes;Clostridia;Clostridiales
Otu1148	3	Bacteria;Firmicutes;Bacilli;Lactobacillales;Aerococcaceae;Facklamia
Otu1055	2	Bacteria;Bacteroidetes;Bacteroidia;Bacteroidales;Prevotellaceae;Prevotella
Otu1054	3	Bacteria;Actinobacteria;Actinobacteria;Actinomycetales
Otu1057	5	Bacteria
Otu1056	2	Bacteria
Otu538	16	Bacteria;Firmicutes;Clostridia;Clostridiales;Ruminococcaceae
Otu539	20	Bacteria;Bacteroidetes;Bacteroidia;Bacteroidales;Bacteroidaceae;Bacteroides
Otu1053	2	Bacteria;Firmicutes;Clostridia;Clostridiales

Otu1052	3	Bacteria;Firmicutes;Clostridia;Clostridiales
Otu534	20	Bacteria;Firmicutes;Clostridia;Clostridiales;Mogibacteriaceae
Otu535	13	Bacteria;Firmicutes;Clostridia;Clostridiales;Ruminococcaceae
Otu536	18	Bacteria;Fibrobacteres;Fibrobacteria;Fibrobacterales;Fibrobacteraceae;Fibrobacter
Otu537	32	Bacteria;Firmicutes;Clostridia;Clostridiales
Otu530	8	Bacteria;Proteobacteria;Gammaproteobacteria;Thiotrichales;Piscirickettsiaceae
Otu531	21	Bacteria;Spirochaetes;Spirochaetes
Otu532	21	Bacteria;Verrucomicrobia;Verruco-5;WCHB1-41;RFP12
Otu533	20	Bacteria;Bacteroidetes;Bacteroidia;Bacteroidales;Paraprevotellaceae;YRC22
Otu1119	3	Bacteria
Otu973	5	Bacteria;Firmicutes;Clostridia;Clostridiales;Mogibacteriaceae
Otu987	3	Bacteria;Verrucomicrobia;Verruco-5;WCHB1-41;RFP12
Otu329	65	Bacteria;Proteobacteria;Betaproteobacteria;Burkholderiales;Alcaligenaceae
Otu328	42	Bacteria;Firmicutes;Clostridia;Clostridiales;Lachnospiraceae;Butyrivibrio
Otu499	9	Bacteria;Proteobacteria;Alphaproteobacteria;RF32
Otu498	8	Bacteria;Proteobacteria;Gammaproteobacteria;Alteromonadales;Alteromonadaceae;Cellvibrio
Otu325	24	Bacteria;Verrucomicrobia;Verruco-5;WCHB1-41;RFP12
Otu324	58	Bacteria;Bacteroidetes;Bacteroidia;Bacteroidales;Bacteroidaceae
Otu327	28	Bacteria;Tenericutes;RF3;ML615J-28
Otu326	66	Bacteria;Firmicutes;Clostridia;Clostridiales
Otu321	24	Bacteria;Firmicutes;Clostridia;Clostridiales;Tissierellaceae
Otu320	46	Bacteria;Firmicutes;Clostridia;Clostridiales;Lachnospiraceae
Otu323	76	Bacteria;Firmicutes;Clostridia;Clostridiales;Ruminococcaceae;Oscillospira
Otu322	60	Bacteria;Bacteroidetes;Bacteroidia;Bacteroidales
Otu975	6	Bacteria;Firmicutes;Clostridia;Clostridiales;Ruminococcaceae
Otu302	44	Bacteria;Verrucomicrobia;Verruco-5;WCHB1-41;RFP12

Otu958	4	Bacteria;Tenericutes;Mollicutes;RF39
Otu742	3	Bacteria;Firmicutes;Clostridia;Clostridiales;Tissierellaceae
Otu1118	3	Bacteria;Firmicutes;Clostridia;Clostridiales;Ruminococcaceae
Otu408	34	Bacteria;Firmicutes;Clostridia;Clostridiales;Caldicoprobacteraceae;Caldicoprobacter
Otu409	34	Bacteria;Firmicutes;Clostridia;Clostridiales;Lachnospiraceae;Oribacterium
Otu1123	2	Bacteria;Proteobacteria
Otu1122	2	Bacteria;Tenericutes;Mollicutes;RF39
Otu1125	2	Bacteria;Firmicutes;Clostridia;Clostridiales;Ruminococcaceae
Otu1124	7	Bacteria;Firmicutes;Clostridia;Clostridiales;Ruminococcaceae;Oscillospira
Otu1127	2	Bacteria;Proteobacteria;Betaproteobacteria;Burkholderiales;Burkholderiaceae;Salinispora;Salinispora_tropica
Otu1126	2	Bacteria;Firmicutes;Clostridia;Clostridiales;Ruminococcaceae
Otu400	28	Bacteria;Firmicutes;Clostridia;Clostridiales;Ruminococcaceae;Ruminococcus
Otu401	26	Bacteria;Bacteroidetes;Bacteroidia;Bacteroidales;Paraprevotellaceae;Prevotella
Otu402	36	Bacteria;Bacteroidetes;Bacteroidia;Bacteroidales;Bacteroidaceae;Bacteroides
Otu403	17	Bacteria;Firmicutes;Clostridia;Clostridiales
Otu404	20	Bacteria;Bacteroidetes;Bacteroidia;Bacteroidales;S24-7
Otu405	48	Bacteria;Proteobacteria;Betaproteobacteria;Burkholderiales
Otu406	42	Bacteria;Proteobacteria;Betaproteobacteria;Burkholderiales;Alcaligenaceae
Otu407	27	Bacteria;Firmicutes;Clostridia;Clostridiales;Ruminococcaceae;Faecalibacterium;Faecalibacterium_prausnitzii
Otu149	361	Bacteria;Firmicutes;Clostridia;Clostridiales;Veillonellaceae;Mitsuokella
Otu148	88	Bacteria;Firmicutes;Clostridia;Clostridiales;Tissierellaceae;Tissierella_Soehngenia
Otu662	17	Bacteria;Firmicutes;Clostridia;Clostridiales;Christensenellaceae
Otu663	10	Bacteria;Bacteroidetes;Bacteroidia;Bacteroidales;Bacteroidaceae;Bacteroides
Otu664	13	Bacteria;Firmicutes;Clostridia;Clostridiales;Ruminococcaceae;Oscillospira
Otu665	23	Bacteria;Firmicutes;Clostridia;Clostridiales;Christensenellaceae
Otu666	5	Bacteria;Firmicutes;Clostridia;Clostridiales;Lachnospiraceae

Otu667	16	Bacteria;Firmicutes;Clostridia;Clostridiales;Lachnospiraceae;Blautia
Otu141	352	Bacteria;Firmicutes;Clostridia;Clostridiales;Clostridiaceae;02d06
Otu140	94	Bacteria;Firmicutes;Clostridia;Clostridiales;Clostridiaceae;Clostridium;Clostridium_tetani
Otu143	101	Bacteria;Firmicutes;Clostridia;Clostridiales;Veillonellaceae
Otu142	176	Bacteria;Bacteroidetes;Bacteroidia;Bacteroidales
Otu145	107	Bacteria;Bacteroidetes;Flavobacteriia;Flavobacteriales;Flavobacteriaceae;Myroides;Myroides_odoratimimus
Otu144	177	Bacteria;Firmicutes;Clostridia;Clostridiales;Veillonellaceae;Anaerovibrio
Otu147	66	Bacteria;Firmicutes;Clostridia;Clostridiales;Veillonellaceae;Selenomonas;Selenomonas_lacticifex
Otu146	168	Bacteria;Tenericutes;Mollicutes;Acholeplasmatales;Acholeplasmataceae;Acholeplasma
Otu390	24	Bacteria;Firmicutes;Clostridia;Clostridiales
Otu391	21	Bacteria;Firmicutes;Clostridia;Clostridiales;Lachnospiraceae;Lachnospira
Otu392	26	Bacteria;Firmicutes;Clostridia;Clostridiales;Ruminococcaceae
Otu393	23	Bacteria;Bacteroidetes;Bacteroidia;Bacteroidales
Otu394	24	Bacteria;Firmicutes;Clostridia;Clostridiales;Christensenellaceae
Otu395	16	Bacteria;Bacteroidetes;Bacteroidia;Bacteroidales;Porphyromonadaceae;Porphyromonas
Otu49	1397	Bacteria;Bacteroidetes;Bacteroidia;Bacteroidales;Prevotellaceae;Prevotella
Otu48	737	Bacteria;Tenericutes;Mollicutes;Acholeplasmatales;Acholeplasmataceae
Otu47	900	Bacteria;Bacteroidetes;Bacteroidia;Bacteroidales;Paraprevotellaceae;CF231
Otu46	608	Bacteria;Proteobacteria;Gammaproteobacteria;Enterobacteriales;Enterobacteriaceae;Providencia
Otu45	1197	Bacteria;Firmicutes;Clostridia;Clostridiales;Lachnospiraceae
Otu44	504	Bacteria;Bacteroidetes;Flavobacteriia;Flavobacteriales;Flavobacteriaceae
Otu43	835	Bacteria;Bacteroidetes
Otu42	623	Bacteria;Bacteroidetes;Bacteroidia;Bacteroidales;Prevotellaceae;Prevotella
Otu41	1246	Bacteria;Bacteroidetes;Bacteroidia;Bacteroidales;Prevotellaceae;Prevotella
Otu40	1529	Bacteria;Firmicutes;Clostridia;Clostridiales;Christensenellaceae
Otu1121	2	Bacteria;Firmicutes;Clostridia;Clostridiales

Otu1120	3	Bacteria;Firmicutes;Clostridia;Clostridiales;Lachnospiraceae;Clostridium
Otu1144	5	Bacteria;Firmicutes;Clostridia;Clostridiales;Ruminococcaceae
Otu242	21	Bacteria;Proteobacteria;Gammaproteobacteria;Xanthomonadales;Xanthomonadaceae
Otu243	122	Bacteria;Bacteroidetes;Bacteroidia;Bacteroidales;Prevotellaceae;Prevotella
Otu240	80	Bacteria;Proteobacteria;Gammaproteobacteria;Xanthomonadales;Xanthomonadaceae
Otu241	77	Bacteria;Spirochaetes;Spirochaetes;Spirochaetales;Spirochaetaceae;Treponema
Otu246	43	Bacteria;Firmicutes;Clostridia;Clostridiales;Lachnospiraceae
Otu247	48	Bacteria;Proteobacteria;Alphaproteobacteria;Sphingomonadales;Sphingomonadaceae
Otu244	87	Bacteria;Firmicutes;Clostridia;Clostridiales;Clostridiaceae;Clostridium;Clostridium_butyricum
Otu245	45	Bacteria;Firmicutes;Clostridia;Clostridiales;Ruminococcaceae
Otu248	58	Bacteria;Tenericutes;Mollicutes;Acholeplasmatales;Acholeplasmataceae;Acholeplasma
Otu249	127	Bacteria;Firmicutes;Clostridia;Clostridiales;Ruminococcaceae
Otu718	123	Bacteria;Bacteroidetes;Bacteroidia;Bacteroidales;Porphyromonadaceae
Otu719	4	Bacteria;Firmicutes;Clostridia;Clostridiales;Ruminococcaceae
Otu1142	3	Bacteria;Firmicutes;Clostridia;Clostridiales;Tissierellaceae
Otu813	9	Bacteria;Firmicutes;Clostridia;Clostridiales
Otu812	6	Bacteria;Firmicutes;Clostridia;Clostridiales
Otu811	2	Bacteria;Firmicutes;Clostridia;Clostridiales
Otu472	14	Bacteria;Tenericutes;Mollicutes;RF39
Otu817	5	Bacteria;Bacteroidetes;Bacteroidia;Bacteroidales;Prevotellaceae;Prevotella
Otu816	5	Bacteria;Proteobacteria;Deltaproteobacteria;Desulfovibrionales;Desulfovibrionaceae
Otu815	5	Bacteria;Proteobacteria;Deltaproteobacteria;Bdellovibrionales;Bacteriovoracaceae
Otu814	8	Bacteria;Tenericutes;Mollicutes;RF39
Otu819	12	Bacteria;Bacteroidetes;Flavobacteriia;Flavobacteriales;Flavobacteriaceae
Otu818	3	Bacteria
Otu1154	4	Bacteria;Firmicutes;Bacilli;Lactobacillales;Aerococcaceae

Otu1039	5	Bacteria;Firmicutes;Clostridia;Clostridiales
Otu1153	2	Bacteria;Firmicutes
Otu963	6	Bacteria;Bacteroidetes;Bacteroidia;Bacteroidales
Otu478	22	Bacteria;Firmicutes;Clostridia;Clostridiales;Ruminococcaceae
Otu1150	32	Bacteria;Firmicutes;Clostridia;Clostridiales
Otu660	3	Bacteria;Firmicutes
Otu918	7	Bacteria;Firmicutes;Clostridia;Clostridiales
Otu661	9	Bacteria;Firmicutes;Clostridia;Clostridiales;Lachnospiraceae
Otu919	10	Bacteria;Firmicutes;Clostridia;Clostridiales;Lachnospiraceae
Otu925	3	Bacteria;Bacteroidetes
Otu924	31	Bacteria;Bacteroidetes
Otu888	4	Bacteria;Firmicutes;Clostridia;Clostridiales;EtOH8
Otu889	9	Bacteria;Bacteroidetes;Flavobacteriia;Flavobacteriales;Flavobacteriaceae;Aequorivita
Otu866	159	Bacteria;Proteobacteria;Gammaproteobacteria;Pseudomonadales;Moraxellaceae;Acinetobacter
Otu867	25	Bacteria;Cyanobacteria;4C0d-2;YS2
Otu864	3	Bacteria;Firmicutes;Clostridia;Clostridiales
Otu865	3	Bacteria;Bacteroidetes;Bacteroidia;Bacteroidales;Prevotellaceae;Prevotella
Otu862	3	Bacteria;Firmicutes;Clostridia;Clostridiales
Otu863	2	Bacteria
Otu860	7	Bacteria;Firmicutes;Clostridia;Clostridiales;Ruminococcaceae
Otu861	2	Bacteria;Actinobacteria;Actinobacteria;Actinomycetales;Microbacteriaceae;Pseudoclavibacter;Pseudoclavibacter_bifida
Otu1091	3	Bacteria;Bacteroidetes;Flavobacteriia;Flavobacteriales;Flavobacteriaceae
Otu912	3	Bacteria;Firmicutes;Clostridia;Clostridiales;Lachnospiraceae;Coprococcus
Otu1093	4	Bacteria;Firmicutes;Clostridia;Clostridiales;Lachnospiraceae
Otu920	2	Bacteria;Proteobacteria;Betaproteobacteria;Burkholderiales;Alcaligenaceae
Otu1095	5	Bacteria;Firmicutes;Clostridia;Clostridiales;Ruminococcaceae

Otu1094	53	Bacteria;Proteobacteria;Gammaproteobacteria
Otu868	11	Bacteria;Firmicutes;Clostridia;Clostridiales
Otu869	6	Bacteria;Bacteroidetes
Otu1073	2	Bacteria;Bacteroidetes;Bacteroidia;Bacteroidales
Otu668	60	Bacteria;Firmicutes;Clostridia;Clostridiales;Christensenellaceae
Otu669	4	Bacteria;Firmicutes;Clostridia;Clostridiales;Ruminococcaceae
Otu1015	7	Bacteria;Proteobacteria;Betaproteobacteria;Burkholderiales;Alcaligenaceae;Sutterella
Otu886	2	Bacteria;Bacteroidetes;Bacteroidia;Bacteroidales;Porphyromonadaceae;Dysgonomonas
Otu1014	2	Bacteria;Actinobacteria;Coriobacteriia;Coriobacteriales;Coriobacteriaceae
Otu598	9	Bacteria;Verrucomicrobia;Verruco-5;WCHB1-41;RFP12
Otu599	4	Bacteria;Firmicutes;Clostridia;Clostridiales;Tissierellaceae;Sedimentibacter
Otu1090	4	Bacteria;Bacteroidetes;Bacteroidia;Bacteroidales;Bacteroidaceae;Bacteroides
Otu592	3780	Bacteria;Bacteroidetes;Bacteroidia;Bacteroidales;Prevotellaceae;Prevotella;Prevotella_copri
Otu593	3	Bacteria;Proteobacteria;Gammaproteobacteria
Otu590	10	Bacteria;Verrucomicrobia;Verruco-5;WCHB1-41;RFP12
Otu591	11	Bacteria;Firmicutes;Clostridia;Clostridiales;Mogibacteriaceae
Otu596	7	Bacteria;Firmicutes;Erysipelotrichi;Erysipelotrichales;Erysipelotrichaceae;RFN20
Otu597	4	Bacteria;Bacteroidetes;Bacteroidia;Bacteroidales;Bacteroidaceae;Bacteroides;Bacteroides_uniformis
Otu594	3	Bacteria;Proteobacteria;Alphaproteobacteria
Otu595	7	Bacteria;Bacteroidetes;Bacteroidia;Bacteroidales
Otu1019	2	Bacteria;Thermi;Deinococci;Deinococcales;Trueperaceae;B-42
Otu502	11	Bacteria;Tenericutes;Mollicutes;Acholeplasmatales;Acholeplasmataceae;Acholeplasma
Otu882	2	Bacteria;Firmicutes;Clostridia;Clostridiales
Otu931	3	Bacteria
Otu1018	9	Bacteria;Verrucomicrobia;Verrucomicrobiae;Verrucomicrobiales;Verrucomicrobiaceae;Akkermansia
Otu883	3	Bacteria;Firmicutes;Clostridia;Clostridiales;Ruminococcaceae

Otu1152	2	Bacteria;Firmicutes;Clostridia;Clostridiales;Ruminococcaceae
Otu567	5	Bacteria;Verrucomicrobia;Verruco-5;WCHB1-41;RFP12
Otu566	12	Bacteria;Bacteroidetes;Sphingobacteriia;Sphingobacteriales;Sphingobacteriaceae;Sphingobacterium
Otu565	9	Bacteria;Firmicutes;Clostridia;Clostridiales
Otu564	10	Bacteria;Firmicutes;Clostridia;Clostridiales;Tissierellaceae;Peptoniphilus
Otu448	25	Bacteria;Firmicutes;Clostridia;Clostridiales;Lachnospiraceae
Otu449	9	Bacteria;Tenericutes;Mollicutes;RF39
Otu561	46	Bacteria;Firmicutes;Clostridia;Clostridiales
Otu560	16	Bacteria;Spirochaetes;Spirochaetes;Spirochaetales;Spirochaetaceae;Treponema
Otu444	12	Bacteria;Firmicutes;Clostridia;Clostridiales;Ruminococcaceae
Otu445	17	Bacteria;Firmicutes;Clostridia;Clostridiales;Christensenellaceae
Otu446	19	Bacteria;Firmicutes;Clostridia;Clostridiales
Otu447	20	Bacteria;Firmicutes;Clostridia;Clostridiales;Ruminococcaceae
Otu440	20	Bacteria;Firmicutes;Clostridia;Clostridiales
Otu441	14	Bacteria;Bacteroidetes;Bacteroidia;Bacteroidales
Otu442	45	Bacteria;Firmicutes;Clostridia;Clostridiales;Veillonellaceae;Phascolarctobacterium
Otu443	18	Bacteria;Firmicutes;Bacilli;Turicibacterales;Turicibacteraceae;Turicibacter
Otu932	3	Bacteria;Cyanobacteria;4C0d-2;YS2
Otu1156	4	Bacteria;Firmicutes;Clostridia;Clostridiales;Clostridiaceae;Clostridium
Otu1008	5	Bacteria;Bacteroidetes;Bacteroidia;Bacteroidales;Porphyromonadaceae;Dysgonomonas
Otu508	11	Bacteria;Bacteroidetes;Bacteroidia;Bacteroidales;Paraprevotellaceae;CF231
Otu1006	2	Bacteria;Bacteroidetes;Bacteroidia;Bacteroidales;Porphyromonadaceae;Parabacteroides
Otu1007	2	Bacteria;Firmicutes;Clostridia;Clostridiales
Otu1004	3	Bacteria;Firmicutes;Clostridia;Clostridiales;Ruminococcaceae
Otu1005	5	Bacteria;Firmicutes;Clostridia;Clostridiales
Otu1002	3	Bacteria;Firmicutes;Clostridia;Clostridiales;Ruminococcaceae

Otu1003	14	Bacteria;Bacteroidetes;Bacteroidia;Bacteroidales;Prevotellaceae;Prevotella
Otu1000	6	Bacteria;Proteobacteria;Gammaproteobacteria;Aeromonadales;Aeromonadaceae
Otu1001	90	Bacteria;Proteobacteria;Alphaproteobacteria;Rhizobiales
Otu750	2	Bacteria;Firmicutes;Clostridia;Clostridiales
Otu969	2	Bacteria;Firmicutes;Clostridia;Clostridiales;Ruminococcaceae
Otu968	4	Bacteria;Firmicutes;Clostridia;Clostridiales;Ruminococcaceae
Otu219	208	Bacteria;Firmicutes;Clostridia;Clostridiales;Ruminococcaceae
Otu218	104	Bacteria;Firmicutes;Clostridia;Clostridiales;Ruminococcaceae;Ruminococcus
Otu749	2	Bacteria;Firmicutes;Clostridia;Clostridiales
Otu748	4	Bacteria;Bacteroidetes;Bacteroidia;Bacteroidales;Prevotellaceae;Prevotella
Otu215	81	Bacteria;Tenericutes;Mollicutes;Acholeplasmatales;Acholeplasmataceae;Acholeplasma
Otu214	72	Bacteria;Firmicutes;Clostridia;Clostridiales;Lachnospiraceae
Otu217	61	Bacteria;Firmicutes;Clostridia;Clostridiales;Lachnospiraceae
Otu216	100	Bacteria;Verrucomicrobia;Verruco-5;WCHB1-41;RFP12
Otu211	62	Bacteria;Firmicutes;Clostridia;Clostridiales;Christensenellaceae
Otu210	154	Bacteria;Bacteroidetes;Bacteroidia;Bacteroidales;S24-7
Otu213	50	Bacteria;Firmicutes;Bacilli;Bacillales;Paenibacillaceae;Paenibacillus
Otu212	97	Bacteria;Firmicutes;Clostridia;Clostridiales;Ruminococcaceae;Ruminococcus
Otu398	109	Bacteria;Firmicutes;Clostridia;Clostridiales;Mogibacteriaceae
Otu753	16	Bacteria;Bacteroidetes;Bacteroidia;Bacteroidales;Prevotellaceae;Prevotella;Prevotella_copri
Otu905	4	Bacteria;Firmicutes;Clostridia;Clostridiales;Lachnospiraceae;Coprococcus
Otu399	51	Bacteria;Firmicutes;Clostridia;Clostridiales
Otu978	8	Bacteria;Firmicutes;Clostridia;Clostridiales
Otu979	2	Bacteria;Firmicutes;Clostridia;Clostridiales;Lachnospiraceae
Otu691	5	Bacteria;Actinobacteria;Coriobacteriia;Coriobacteriales;Coriobacteriaceae;Slackia
Otu690	19	Bacteria;Firmicutes;Clostridia;Clostridiales;Ruminococcaceae

Otu693	13	Bacteria;Bacteroidetes;Bacteroidia;Bacteroidales
Otu692	8	Bacteria;Firmicutes;Bacilli;Lactobacillales;Lactobacillaceae;Lactobacillus;Lactobacillus_mucosae
Otu695	4	Bacteria;Tenericutes;Mollicutes;RF39
Otu694	3	Bacteria;Elusimicrobia;Elusimicrobia;Elusimicrobiales;Elusimicrobiaceae
Otu697	16	Bacteria;Proteobacteria;Betaproteobacteria;Burkholderiales;Alcaligenaceae;Sutterella
Otu696	12	Bacteria;Fibrobacteres;Fibrobacteria;Fibrobacterales;Fibrobacteraceae;Fibrobacter;Fibrobacter_succinogenes
Otu699	7	Bacteria;Proteobacteria;Gammaproteobacteria;Enterobacteriales;Enterobacteriaceae
Otu698	6	Bacteria;Proteobacteria;Betaproteobacteria;Burkholderiales;Alcaligenaceae
Otu757	2	Bacteria;Firmicutes;Clostridia;Clostridiales;Ruminococcaceae
Otu361	16	Bacteria;Tenericutes;Mollicutes;Acholeplasmatales;Acholeplasmataceae;Acholeplasma
Otu360	145	Bacteria;Firmicutes;Clostridia;Clostridiales;Tissierellaceae
Otu363	14	Bacteria;Firmicutes;Clostridia;Clostridiales;Clostridiaceae;Clostridium
Otu362	32	Bacteria;Tenericutes;Mollicutes;RF39
Otu365	60	Bacteria;Firmicutes;Clostridia;Clostridiales;Ruminococcaceae;Oscillospira
Otu364	96	Bacteria;Firmicutes;Clostridia;Clostridiales;Ruminococcaceae;Oscillospira
Otu367	77	Bacteria;Firmicutes;Clostridia;Clostridiales;Lachnospiraceae;Anaerostipes
Otu366	27	Bacteria;Proteobacteria;Betaproteobacteria;Rhodocyclales;Rhodocyclaceae;Thauera
Otu369	34	Bacteria;Bacteroidetes;Flavobacteriia;Flavobacteriales;Weeksellaceae
Otu368	27	Bacteria;Bacteroidetes;Bacteroidia;Bacteroidales;S24-7
Otu613	3	Bacteria;Spirochaetes;Spirochaetes;Sphaerochaetales;Sphaerochaetaceae;Sphaerochaeta
Otu621	9	Bacteria;Firmicutes;Clostridia;Clostridiales;Mogibacteriaceae
Otu130	113	Bacteria;Tenericutes;Mollicutes;Acholeplasmatales;Acholeplasmataceae;Acholeplasma
Otu131	122	Bacteria;Proteobacteria;Gammaproteobacteria;Thiotrichales;Piscirickettsiaceae;Methylophaga
Otu132	158	Bacteria;Proteobacteria;Gammaproteobacteria
Otu133	158	Bacteria;Firmicutes;Clostridia;Clostridiales;Ruminococcaceae;Oscillospira
Otu134	123	Bacteria;Bacteroidetes;Bacteroidia;Bacteroidales;Paraprevotellaceae;Prevotella

Otu135	113	Bacteria;Bacteroidetes;Bacteroidia;Bacteroidales
Otu136	160	Bacteria;Bacteroidetes;Flavobacteriia;Flavobacteriales;Flavobacteriaceae
Otu137	266	Bacteria;Spirochaetes;Spirochaetes;Spirochaetales;Spirochaetaceae;Treponema
Otu138	79	Bacteria;Firmicutes;Clostridia;Clostridiales
Otu139	95	Bacteria;Proteobacteria;Gammaproteobacteria;Aeromonadales;Succinivibrionaceae
Otu614	15	Bacteria;Firmicutes;Clostridia;Clostridiales;Clostridiaceae
Otu14	4482	Bacteria;Bacteroidetes;Bacteroidia;Bacteroidales;Porphyromonadaceae
Otu15	4842	Bacteria;Bacteroidetes;Bacteroidia;Bacteroidales;Prevotellaceae;Prevotella
Otu16	3495	Bacteria;Bacteroidetes;Bacteroidia;Bacteroidales;Prevotellaceae;Prevotella;Prevotella_copri
Otu17	2411	Bacteria;Bacteroidetes;Bacteroidia;Bacteroidales;Paraprevotellaceae;YRC22
Otu10	4362	Bacteria;Proteobacteria;Gammaproteobacteria;Xanthomonadales;Xanthomonadaceae;Ignatzschineria
Otu11	3562	Bacteria;Bacteroidetes;Bacteroidia;Bacteroidales;Prevotellaceae;Prevotella
Otu12	3064	Bacteria;Firmicutes;Bacilli;Lactobacillales;Streptococcaceae;Streptococcus;Streptococcus_luteciae
Otu13	2875	Bacteria;Bacteroidetes;Sphingobacteriia;Sphingobacteriales;Sphingobacteriaceae
Otu916	3	Bacteria;Firmicutes;Clostridia;Clostridiales;Ruminococcaceae
Otu917	2	Bacteria;Firmicutes
Otu914	195	Bacteria;Firmicutes;Clostridia;Clostridiales;Ruminococcaceae
Otu616	3	Bacteria;Firmicutes
Otu18	2849	Bacteria;Bacteroidetes;Flavobacteriia;Flavobacteriales;Flavobacteriaceae;Flavobacterium;Flavobacterium_gelidilacus
Otu19	1101	Bacteria;Proteobacteria;Gammaproteobacteria;Aeromonadales;Succinivibrionaceae;Succinivibrio
Otu910	5	Bacteria;Firmicutes;Clostridia;Clostridiales;Ruminococcaceae
Otu911	2	Bacteria;Bacteroidetes;Bacteroidia;Bacteroidales
Otu1086	2	Bacteria
Otu706	19	Bacteria;Actinobacteria;Coriobacteriia;Coriobacteriales;Coriobacteriaceae
Otu1063	44	Bacteria;Firmicutes;Bacilli;Lactobacillales;Lactobacillaceae;Lactobacillus
Otu951	14	Bacteria;Firmicutes;Clostridia;Clostridiales;Ruminococcaceae

Otu1087	6	Bacteria;Firmicutes;Clostridia;Clostridiales;Ruminococcaceae
Otu628	7	Bacteria;Firmicutes;Clostridia;Clostridiales
Otu629	15	Bacteria;Proteobacteria;Alphaproteobacteria;Sphingomonadales
Otu624	12	Archaea;Euryarchaeota;Thermoplasmata;E2;Methanomassiliicoccaceae;vadinCA11
Otu625	6	Bacteria;Proteobacteria;Gammaproteobacteria;Oceanospirillales;Halomonadaceae;Halomonas
Otu626	9	Bacteria;Firmicutes;Clostridia;Clostridiales;Ruminococcaceae
Otu627	3	Bacteria;Firmicutes;Clostridia;Clostridiales
Otu189	71	Bacteria;Firmicutes;Clostridia;Clostridiales;Tissierellaceae;Peptoniphilus
Otu188	171	Bacteria;Firmicutes;Clostridia;Clostridiales;Ruminococcaceae;Ruminococcus
Otu622	17	Bacteria;Firmicutes;Clostridia;Clostridiales;Tissierellaceae
Otu623	5	Bacteria;Cyanobacteria;4C0d-2;YS2
Otu185	120	Bacteria;Bacteroidetes;Bacteroidia;Bacteroidales
Otu184	168	Bacteria;Spirochaetes;Spirochaetes;Spirochaetales;Spirochaetaceae;Treponema
Otu187	138	Bacteria;Firmicutes;Clostridia;Clostridiales;Lachnospiraceae
Otu186	150	Bacteria;Firmicutes;Bacilli;Lactobacillales;Aerococcaceae;Facklamia
Otu181	118	Bacteria;Bacteroidetes;Bacteroidia;Bacteroidales;S24-7
Otu180	68	Bacteria;Bacteroidetes;Bacteroidia;Bacteroidales;Porphyromonadaceae
Otu183	121	Bacteria;Firmicutes;Clostridia;Clostridiales;Tissierellaceae;Sedimentibacter
Otu182	1850	Bacteria;Proteobacteria;Gammaproteobacteria;Pseudomonadales;Pseudomonadaceae
