# Supplementary material for: Chrysomya megacephala larvae feeding favourably influences manure microbiome, heavy metal stability and greenhouse gas emissions
Source: Microb Biotechnol. 2018 Mar 14;11(3):498–509. doi: 10.1111/1751-7915.13253 (PMC5902325; doi:10.1111/1751-7915.13253)
Supplement: Supplementary file 4 — Table S2 Detailed OTUs of RSM, NSM and CMSM in Venn diagram. [file MBT2-11-498-s004.pdf]

group shareOTU_num/uniuqeOTU_num OTU_ID

RSM-vs-NSM604

Otu1,Otu10,Otu100,Otu1003,Otu1008,Otu1009,Otu101,Otu1010,Otu1012,Otu1018,Otu1026,Otu1027,Otu1029,Otu1034,Otu104,Otu1040,Otu1042,Otu1043,Otu1049,Otu105,Otu1051,Otu1057,Otu1058,Otu1063,Otu1064,Otu107,Otu1075,Otu1077,Otu108,Otu1080,Otu1081,Otu1090,Otu1099,Otu11,Otu1100,Otu1105,Otu1107,Otu1110,Otu1112,Otu1113,Otu1114,Otu1116,Otu1124,Otu1128,Otu113,Otu1130,Otu1133,Otu1136,Otu1137,Otu1139,Otu114,Otu1140,Otu1143,Otu1144,Otu1150,Otu1155,Otu1158,Otu1160,Otu119,Otu12,Otu120,Otu122,Otu124,Otu125,Otu126,Otu128,Otu129,Otu133,Otu134,Otu137,Otu139,Otu14,Otu141,Otu142,Otu143,Otu144,Otu145,Otu147,Otu148,Otu149,Otu15,Otu150,Otu153,Otu154,Otu156,Otu157,Otu158,Otu159,Otu16,Otu160,Otu161,Otu162,Otu163,Otu164,Otu167,Otu17,Otu170,Otu172,Otu173,Otu175,Otu177,Otu178,Otu179,Otu18,Otu181,Otu182,Otu184,Otu185,Otu186,Otu187,Otu188,Otu19,Otu191,Otu192,Otu193,Otu195,Otu196,Otu197,Otu198,Otu199,Otu2,Otu20,Otu201,Otu202,Otu203,Otu204,Otu205,Otu206,Otu207,Otu208,Otu209,Otu210,Otu211,Otu212,Otu214,Otu216,Otu218,Otu219,Otu22,Otu222,Otu224,Otu225,Otu228,Otu229,Otu230,Otu231,Otu232,Otu233,Otu235,Otu236,Otu237,Otu239,Otu24,Otu240,Otu243,Otu244,Otu246,Otu249,Otu25,Otu251,Otu252,Otu253,Otu254,Otu255,Otu256,Otu258,Otu259,Otu26,Otu261,Otu262,Otu263,Otu264,Otu265,Otu266,Otu267,Otu268,Otu271,Otu273,Otu277,Otu278,Otu28,Otu280,Otu281,Otu283,Otu286,Otu287,Otu288,Otu289,Otu294,Otu295,Otu296,Otu298,Otu299,Otu3,Otu30,Otu300,Otu301,Otu302,Otu303,Otu304,Otu305,Otu306,Otu307,Otu308,Otu309,Otu31,Otu310,Otu313,Otu314,Otu315,Otu317,Otu318,Otu319,Otu32,Otu320,Otu321,Otu322,Otu323,Otu324,Otu325,Otu326,Otu328,Otu33,Otu330,Otu331,Otu333,Otu334,Otu336,Otu337,Otu338,Otu339,Otu34,Otu341,Otu342,Otu343,Otu344,Otu345,Otu346,Otu347,Otu348,Otu349,Otu35,Otu352,Otu353,Otu354,Otu355,Otu357,Otu360,Otu362,Otu364,Otu365,Otu367,Otu368,Otu372,Otu374,Otu376,Otu377,Otu378,Otu379,Otu382,Otu383,Otu387,Otu389,Otu39,Otu390,Otu392,Otu393,Otu394,Otu396,Otu397,Otu398,Otu399,Otu4,Otu40,Otu400,Otu401,Otu402,Otu403,Otu404,Otu407,Otu409,Otu41,Otu411,Otu414,Otu415,Otu417,Otu418,Otu419,Otu42,Otu420,Otu421,Otu423,Otu424,Otu426,Otu427,Otu428,Otu430,Otu431,Otu432,Otu434,Otu435,Otu439,Otu44,Otu440,Otu442,Otu443,Otu445,Otu446,Otu447,Otu448,Otu449,Otu45,Otu450,Otu452,Otu453,Otu454,Otu455,Otu456,Otu458,Otu459,Otu46,Otu464,Otu465,Otu466,Otu467,Otu469,Otu47,Otu470,Otu472,Otu474,Otu475,Otu476,Otu478,Otu480,Otu481,Otu482,Otu485,Otu49,Otu491,Otu492,Otu493,Otu496,Otu499,Otu5,Otu50,Otu501,Otu503,Otu504,Otu506,Otu507,Otu508,Otu509,Otu51,Otu512,Otu514,Otu515,Otu516,Otu517,Otu52,Otu523,Otu525,Otu527,Otu531,Otu532,Otu533,Otu534,Otu535,Otu536,Otu537,Otu539,Otu54,Otu540,Otu541,Otu543,Otu544,Otu545,Otu546,Otu547,Otu55,Otu550,Otu551,Otu555,Otu558,Otu559,Otu56,Otu560,Otu561,Otu563,Otu569,Otu57,Otu570,Otu572,Otu575,Otu578,Otu579,Otu580,Otu581,Otu582,Otu583,Otu584,Otu586,Otu588,Otu59,Otu590,Otu592,Otu597,Otu6,Otu60,Otu602,Otu603,Otu604,Otu607,Otu609,Otu61,Otu610,Otu612,Otu615,Otu617,Otu619,Otu62,Otu622,Otu623,Otu624,Otu625,Otu63,Otu632,Otu633,Otu634,Otu637,Otu638,Otu64,Otu641,Otu642,Otu645,Otu649,Otu65,Otu650,Otu651,Otu653,Otu654,Otu655,Otu656,Otu657,Otu661,Otu662,Otu664,Otu665,Otu667,Otu668,Otu67,Otu670,Otu674,Otu675,Otu676,Otu677,Otu679,Otu68,Otu680,Otu688,Otu689,Otu69,Otu690,Otu692,Otu693,Otu695,Otu697,Otu699,Otu7,Otu70,Otu700,Otu703,Otu706,Otu707,Otu71,Otu710,Otu712,Otu713,Otu716,Otu717,Otu719,Otu72,Otu721,Otu722,Otu725,Otu72

9,Otu73,Otu730,Otu732,Otu735,Otu737,Otu740,Otu748,Otu753,Otu754,Otu755,Otu758,Otu759,Otu76,Otu762,Otu763,Otu764,Otu769,Otu77,Otu772,Otu773,Otu774,Otu776,Otu779,Otu781,Otu782,Otu783,Otu784,Otu79,Otu790,Otu791,Otu793,Otu795,Otu796,Otu798,Otu799,Otu8,Otu80,Otu803,Otu804,Otu808,Otu812,Otu813,Otu814,Otu816,Otu817,Otu819,Otu82,Otu820,Otu824,Otu83,Otu831,Otu832,Otu835,Otu836,Otu839,Otu84,Otu841,Otu842,Otu845,Otu846,Otu848,Otu851,Otu853,Otu856,Otu86,Otu860,Otu862,Otu866,Otu867,Otu868,Otu87,Otu870,Otu872,Otu873,Otu887,Otu89,Otu894,Otu895,Otu897,Otu898,Otu90,Otu900,Otu901,Otu906,Otu91,Otu910,Otu913,Otu914,Otu919,Otu92,Otu921,Otu922,Otu929,Otu93,Otu934,Otu935,Otu940,Otu95,Otu951,Otu959,Otu96,Otu960,Otu963,Otu967,Otu968,Otu972,Otu974,Otu975,Otu977,Otu978,Otu981,Otu983,Otu984,Otu988,Otu99,Otu990,Otu992,Otu993,Otu999

RSM-vs-CMSM 314

Otu1,Otu10,Otu100,Otu101,Otu1021,Otu1022,Otu1027,Otu104,Otu105,Otu1063,Otu107,Otu108,Otu1080,Otu1099,Otu11,Otu1100,Otu1107,Otu1112,Otu113,Otu1134,Otu114,Otu1140,Otu1158,Otu1161,Otu119,Otu12,Otu120,Otu121,Otu122,Otu125,Otu126,Otu128,Otu129,Otu137,Otu139,Otu14,Otu141,Otu142,Otu145,Otu148,Otu15,Otu150,Otu151,Otu154,Otu156,Otu157,Otu159,Otu16,Otu160,Otu161,Otu162,Otu163,Otu164,Otu165,Otu167,Otu17,Otu170,Otu172,Otu175,Otu177,Otu18,Otu182,Otu184,Otu185,Otu186,Otu187,Otu19,Otu191,Otu192,Otu196,Otu197,Otu199,Otu2,Otu20,Otu201,Otu202,Otu203,Otu204,Otu206,Otu207,Otu209,Otu211,Otu212,Otu218,Otu219,Otu22,Otu222,Otu228,Otu229,Otu23,Otu230,Otu231,Otu232,Otu234,Otu235,Otu237,Otu239,Otu24,Otu240,Otu244,Otu249,Otu25,Otu253,Otu254,Otu255,Otu256,Otu258,Otu259,Otu26,Otu262,Otu263,Otu264,Otu266,Otu267,Otu268,Otu277,Otu28,Otu280,Otu281,Otu286,Otu289,Otu294,Otu296,Otu3,Otu300,Otu301,Otu305,Otu308,Otu309,Otu31,Otu310,Otu315,Otu32,Otu321,Otu324,Otu33,Otu331,Otu333,Otu336,Otu338,Otu339,Otu34,Otu341,Otu346,Otu348,Otu35,Otu352,Otu354,Otu360,Otu364,Otu365,Otu367,Otu368,Otu374,Otu377,Otu378,Otu383,Otu387,Otu389,Otu39,Otu390,Otu392,Otu393,Otu394,Otu397,Otu398,Otu399,Otu4,Otu40,Otu400,Otu402,Otu409,Otu41,Otu417,Otu419,Otu42,Otu428,Otu431,Otu44,Otu441,Otu442,Otu45,Otu450,Otu452,Otu455,Otu456,Otu458,Otu459,Otu46,Otu462,Otu466,Otu467,Otu47,Otu470,Otu475,Otu480,Otu482,Otu489,Otu493,Otu496,Otu5,Otu50,Otu500,Otu512,Otu517,Otu52,Otu533,Otu534,Otu535,Otu536,Otu537,Otu539,Otu54,Otu541,Otu542,Otu544,Otu546,Otu55,Otu550,Otu56,Otu560,Otu561,Otu57,Otu570,Otu579,Otu582,Otu584,Otu588,Otu59,Otu598,Otu6,Otu604,Otu606,Otu610,Otu619,Otu62,Otu624,Otu625,Otu63,Otu634,Otu637,Otu64,Otu65,Otu650,Otu653,Otu657,Otu663,Otu664,Otu668,Otu670,Otu671,Otu674,Otu675,Otu679,Otu68,Otu688,Otu69,Otu692,Otu699,Otu7,Otu70,Otu700,Otu706,Otu709,Otu71,Otu72,Otu729,Otu73,Otu737,Otu740,Otu761,Otu769,Otu77,Otu773,Otu776,Otu779,Otu782,Otu790,Otu80,Otu803,Otu804,Otu808,Otu809,Otu812,Otu819,Otu82,Otu820,Otu824,Otu831,Otu835,Otu845,Otu858,Otu866,Otu87,Otu870,Otu873,Otu875,Otu89,Otu898,Otu90,Otu91,Otu914,Otu916,Otu919,Otu92,Otu922,Otu929,Otu93,Otu935,Otu940,Otu95,Otu96,Otu984,Otu99

NSM-vs-CMSM 352

Otu1,Otu10,Otu100,Otu1001,Otu101,Otu1020,Otu1027,Otu103,Otu104,Otu1041,Otu105,Otu1063,Otu107,Otu108,Otu1080,Otu1091,Otu1099,Otu11,Otu110,Otu1100,Otu1107,Otu111,Otu1112,Otu113,Otu1132,Otu114,Otu1140,Otu1141,Otu1156,Otu1157,Otu1158,Otu116,Otu117,Otu119,Otu12,Otu120,Otu122,Otu125,Otu126,Otu127,Otu128,Otu129,Otu13,Otu13

7,Otu139,Otu14,Otu141,Otu142,Otu145,Otu148,Otu15,Otu150,Otu154,Otu156,Otu157,Otu159,Otu16,Otu160,Otu161,Otu162,Otu163,Otu164,Otu167,Otu17,Otu170,Otu171,Otu172,Otu175,Otu177,Otu18,Otu180,Otu182,Otu184,Otu185,Otu186,Otu187,Otu19,Otu191,Otu192,Otu196,Otu197,Otu199,Otu2,Otu20,Otu201,Otu202,Otu203,Otu204,Otu206,Otu207,Otu209,Otu21,Otu211,Otu212,Otu217,Otu218,Otu219,Otu22,Otu221,Otu222,Otu226,Otu228,Otu229,Otu230,Otu231,Otu232,Otu235,Otu237,Otu239,Otu24,Otu240,Otu244,Otu249,Otu25,Otu253,Otu254,Otu255,Otu256,Otu258,Otu259,Otu26,Otu260,Otu262,Otu263,Otu264,Otu266,Otu267,Otu268,Otu270,Otu277,Otu28,Otu280,Otu281,Otu286,Otu289,Otu29,Otu293,Otu294,Otu296,Otu3,Otu300,Otu301,Otu305,Otu308,Otu309,Otu31,Otu310,Otu315,Otu316,Otu32,Otu321,Otu324,Otu33,Otu331,Otu333,Otu336,Otu338,Otu339,Otu34,Otu341,Otu346,Otu348,Otu35,Otu351,Otu352,Otu354,Otu36,Otu360,Otu364,Otu365,Otu367,Otu368,Otu37,Otu374,Otu377,Otu378,Otu38,Otu380,Otu383,Otu387,Otu389,Otu39,Otu390,Otu392,Otu393,Otu394,Otu397,Otu398,Otu399,Otu4,Otu40,Otu400,Otu402,Otu405,Otu409,Otu41,Otu413,Otu417,Otu419,Otu42,Otu428,Otu431,Otu436,Otu44,Otu442,Otu45,Otu450,Otu452,Otu455,Otu456,Otu457,Otu458,Otu459,Otu46,Otu466,Otu467,Otu468,Otu47,Otu470,Otu475,Otu480,Otu482,Otu486,Otu493,Otu495,Otu496,Otu5,Otu50,Otu512,Otu517,Otu52,Otu533,Otu534,Otu535,Otu536,Otu537,Otu539,Otu54,Otu541,Otu544,Otu546,Otu55,Otu550,Otu552,Otu56,Otu560,Otu561,Otu564,Otu566,Otu57,Otu570,Otu573,Otu576,Otu579,Otu58,Otu582,Otu584,Otu588,Otu59,Otu6,Otu604,Otu610,Otu619,Otu62,Otu624,Otu625,Otu63,Otu634,Otu637,Otu64,Otu644,Otu65,Otu650,Otu653,Otu657,Otu66,Otu664,Otu668,Otu670,Otu672,Otu674,Otu675,Otu679,Otu68,Otu681,Otu688,Otu69,Otu692,Otu696,Otu699,Otu7,Otu70,Otu700,Otu706,Otu71,Otu718,Otu72,Otu729,Otu73,Otu737,Otu74,Otu740,Otu745,Otu75,Otu756,Otu769,Otu77,Otu773,Otu776,Otu779,Otu782,Otu790,Otu80,Otu803,Otu804,Otu808,Otu812,Otu815,Otu819,Otu82,Otu820,Otu821,Otu824,Otu829,Otu831,Otu835,Otu845,Otu85,Otu866,Otu87,Otu870,Otu873,Otu88,Otu89,Otu898,Otu9,Otu90,Otu91,Otu914,Otu919,Otu92,Otu922,Otu929,Otu93,Otu935,Otu940,Otu95,Otu952,Otu96,Otu984,Otu99,Otu996

RSM-vs-NSM-vs-CMSM 290

Otu1,Otu10,Otu100,Otu101,Otu1027,Otu104,Otu105,Otu1063,Otu107,Otu108,Otu1080,Otu1099,Otu11,Otu1100,Otu1107,Otu1112,Otu113,Otu114,Otu1140,Otu1158,Otu119,Otu12,Otu120,Otu122,Otu125,Otu126,Otu128,Otu129,Otu137,Otu139,Otu14,Otu141,Otu142,Otu145,Otu148,Otu15,Otu150,Otu154,Otu156,Otu157,Otu159,Otu16,Otu160,Otu161,Otu162,Otu163,Otu164,Otu167,Otu17,Otu170,Otu172,Otu175,Otu177,Otu18,Otu182,Otu184,Otu185,Otu186,Otu187,Otu19,Otu191,Otu192,Otu196,Otu197,Otu199,Otu2,Otu20,Otu201,Otu202,Otu203,Otu204,Otu206,Otu207,Otu209,Otu211,Otu212,Otu218,Otu219,Otu22,Otu222,Otu228,Otu229,Otu230,Otu231,Otu232,Otu235,Otu237,Otu239,Otu24,Otu240,Otu244,Otu249,Otu25,Otu253,Otu254,Otu255,Otu256,Otu258,Otu259,Otu26,Otu262,Otu263,Otu264,Otu266,Otu267,Otu268,Otu277,Otu28,Otu280,Otu281,Otu286,Otu289,Otu294,Otu296,Otu3,Otu300,Otu301,Otu305,Otu308,Otu309,Otu31,Otu310,Otu315,Otu32,Otu321,Otu324,Otu33,Otu331,Otu333,Otu336,Otu338,Otu339,Otu34,Otu341,Otu346,Otu348,Otu35,Otu352,Otu354,Otu360,Otu364,Otu365,Otu367,Otu368,Otu374,Otu377,Otu378,Otu383,Otu387,Otu389,Otu39,Otu390,Otu392,Otu393,Otu394,Otu397,Otu398,Otu399,Otu4,Otu40,Otu400,Otu402,Otu409,Otu41,Otu417,Otu419,Otu42,Otu428,Otu431,Otu44,Otu442,Otu45,Otu450,Otu452,Otu455,Otu456,Otu458,Otu459,Otu46,Otu466,Otu467,Otu47,Otu470,Otu475,Otu480,Otu482,Otu493,Otu496,Otu5,Otu50,Otu512,Otu517,Otu52,Otu533,Otu534,Otu535,Otu536,Otu537,Otu539,Otu5

4,Otu541,Otu544,Otu546,Otu55,Otu550,Otu56,Otu560,Otu561,Otu57,Otu570,Otu579,Otu582,Otu584,Otu588,Otu59,Otu6,Otu604,Otu610,Otu619,Otu62,Otu624,Otu625,Otu63,Otu634,Otu637,Otu64,Otu65,Otu650,Otu653,Otu657,Otu664,Otu668,Otu670,Otu674,Otu675,Otu679,Otu68,Otu688,Otu69,Otu692,Otu699,Otu7,Otu70,Otu700,Otu706,Otu71,Otu72,Otu729,Otu73,Otu737,Otu740,Otu769,Otu77,Otu773,Otu776,Otu779,Otu782,Otu790,Otu80,Otu803,Otu804,Otu808,Otu812,Otu819,Otu82,Otu820,Otu824,Otu831,Otu835,Otu845,Otu866,Otu87,Otu870,Otu873,Otu89,Otu898,Otu90,Otu91,Otu914,Otu919,Otu92,Otu922,Otu929,Otu93,Otu935,Otu940,Otu95,Otu96,Otu984,Otu99

RSM153

Otu1000,Otu1002,Otu1004,Otu1007,Otu1013,Otu1015,Otu1017,Otu1028,Otu1035,Otu1037,Otu1038,Otu1039,Otu1044,Otu1052,Otu1055,Otu1059,Otu1066,Otu1068,Otu1071,Otu1073,Otu1074,Otu1084,Otu1085,Otu1092,Otu1095,Otu1104,Otu1109,Otu1111,Otu1115,Otu1118,Otu1122,Otu1129,Otu1135,Otu1145,Otu1146,Otu1147,Otu1152,Otu1159,Otu290,Otu335,Otu373,Otu375,Otu381,Otu388,Otu391,Otu444,Otu483,Otu484,Otu494,Otu513,Otu520,Otu521,Otu538,Otu548,Otu565,Otu568,Otu574,Otu587,Otu591,Otu594,Otu595,Otu605,Otu608,Otu611,Otu613,Otu616,Otu618,Otu621,Otu628,Otu630,Otu639,Otu643,Otu648,Otu652,Otu658,Otu666,Otu669,Otu673,Otu684,Otu685,Otu694,Otu701,Otu708,Otu715,Otu720,Otu723,Otu731,Otu733,Otu738,Otu739,Otu743,Otu744,Otu746,Otu747,Otu749,Otu751,Otu752,Otu757,Otu765,Otu768,Otu770,Otu775,Otu777,Otu794,Otu797,Otu800,Otu802,Otu805,Otu806,Otu807,Otu810,Otu825,Otu840,Otu847,Otu863,Otu864,Otu865,Otu877,Otu879,Otu880,Otu881,Otu884,Otu893,Otu903,Otu908,Otu912,Otu917,Otu918,Otu926,Otu927,Otu936,Otu939,Otu941,Otu943,Otu944,Otu945,Otu946,Otu949,Otu957,Otu964,Otu966,Otu969,Otu971,Otu976,Otu979,Otu980,Otu985,Otu987,Otu989,Otu991,Otu994,Otu995,Otu997

NSM 91

Otu1006,Otu1014,Otu1023,Otu1030,Otu1033,Otu1045,Otu1050,Otu1067,Otu1072,Otu1079,Otu1086,Otu1093,Otu1097,Otu1098,Otu1101,Otu1102,Otu1106,Otu1108,Otu1121,Otu1127,Otu1131,Otu1148,Otu1149,Otu1153,Otu1154,Otu140,Otu189,Otu194,Otu269,Otu291,Otu350,Otu422,Otu425,Otu518,Otu519,Otu522,Otu554,Otu562,Otu567,Otu596,Otu627,Otu631,Otu646,Otu659,Otu660,Otu683,Otu687,Otu691,Otu704,Otu714,Otu727,Otu736,Otu750,Otu760,Otu767,Otu778,Otu786,Otu787,Otu789,Otu792,Otu801,Otu811,Otu818,Otu823,Otu833,Otu837,Otu838,Otu854,Otu876,Otu882,Otu885,Otu891,Otu896,Otu904,Otu905,Otu909,Otu911,Otu915,Otu931,Otu932,Otu937,Otu948,Otu950,Otu953,Otu955,Otu958,Otu961,Otu962,Otu970,Otu973,Otu998

CMSM 228

Otu1005,Otu1011,Otu1016,Otu1019,Otu102,Otu1024,Otu1025,Otu1031,Otu1032,Otu1036,Otu1046,Otu1047,Otu1048,Otu1053,Otu1054,Otu1056,Otu106,Otu1060,Otu1061,Otu1062,Otu1065,Otu1069,Otu1070,Otu1076,Otu1078,Otu1082,Otu1083,Otu1087,Otu1088,Otu1089,Otu109,Otu1094,Otu1096,Otu1103,Otu1117,Otu1119,Otu112,Otu1120,Otu1123,Otu1125,Otu1126,Otu1138,Otu1142,Otu115,Otu1151,Otu1162,Otu118,Otu123,Otu130,Otu131,Otu132,Otu135,Otu136,Otu138,Otu146,Otu152,Otu155,Otu166,Otu168,Otu169,Otu174,Otu176,Otu183,Otu190,Otu200,Otu213,Otu215,Otu220,Otu223,Otu227,Otu238,Otu241,Otu242,Otu245,Otu247,Otu248,Otu250,Otu257,Otu27,Otu272,Otu274,Otu275,Otu276,Otu279,Otu282,Otu284,Otu285,Otu292,Otu297,Otu311,Otu312,Otu327,Otu329,Otu332,Otu340,Otu356,Otu358,Otu359,Otu361,Otu363,Otu366,Otu369,Otu370,Otu371,Otu384,Otu385,Otu386,Otu395,

Otu406,Otu408,Otu410,Otu412,Otu416,Otu429,Otu43,Otu433,Otu437,Otu438,Otu451,Otu460,Otu461,Otu463,Otu471,Otu473,Otu477,Otu479,Otu48,Otu487,Otu488,Otu490,Otu497,Otu498,Otu502,Otu505,Otu510,Otu511,Otu524,Otu526,Otu528,Otu529,Otu53,Otu530,Otu549,Otu553,Otu556,Otu557,Otu571,Otu577,Otu585,Otu589,Otu593,Otu599,Otu600,Otu601,Otu614,Otu620,Otu626,Otu629,Otu635,Otu636,Otu640,Otu647,Otu678,Otu682,Otu686,Otu698,Otu702,Otu705,Otu711,Otu724,Otu726,Otu728,Otu734,Otu741,Otu742,Otu766,Otu771,Otu78,Otu780,Otu785,Otu788,Otu81,Otu822,Otu826,Otu827,Otu828,Otu830,Otu834,Otu843,Otu844,Otu849,Otu850,Otu852,Otu855,Otu857,Otu859,Otu861,Otu869,Otu871,Otu874,Otu878,Otu883,Otu886,Otu888,Otu889,Otu890,Otu892,Otu899,Otu902,Otu907,Otu920,Otu923,Otu924,Otu925,Otu928,Otu930,Otu933,Otu938,Otu94,Otu942,Otu947,Otu954,Otu956,Otu965,Otu97,Otu98,Otu982,Otu986
