# Supplementary material for: Chrysomya megacephala larvae feeding favourably influences manure microbiome, heavy metal stability and greenhouse gas emissions
Source: Microb Biotechnol. 2018 Mar 14;11(3):498–509. doi: 10.1111/1751-7915.13253 (PMC5902325; doi:10.1111/1751-7915.13253)
Supplement: Supplementary file 5 — Table S3 Beta diversity distance of RSM, NSM and CMSM. [file MBT2-11-498-s005.pdf]

Table S3a bray_curtis_Beta_diversity

	RSM.1	RSM.2	RSM.3	NSM.1	NSM.2	NSM.3	CMSM.1	CMSM.2	CMSM.3
RSM.1	0	0.219592	0.222028	0.361017	0.350972	0.36266	0.697348	0.651782	0.650443
RSM.2	0.219592	0	0.20277	0.311927	0.337101	0.323823	0.699341	0.638246	0.641079
RSM.3	0.222028	0.20277	0	0.357514	0.358416	0.350342	0.706488	0.653162	0.653962
NSM.1	0.361017	0.311927	0.357514	0	0.312442	0.245427	0.685705	0.644827	0.631275
NSM.2	0.350972	0.337101	0.358416	0.312442	0	0.327079	0.635498	0.585024	0.585052
NSM.3	0.36266	0.323823	0.350342	0.245427	0.327079	0	0.689934	0.646197	0.643487
CMSM.1	0.697348	0.699341	0.706488	0.685705	0.635498	0.689934	0	0.290299	0.305048
CMSM.2	0.651782	0.638246	0.653162	0.644827	0.585024	0.646197	0.290299	0	0.318386
CMSM.3	0.650443	0.641079	0.653962	0.631275	0.585052	0.643487	0.305048	0.318386	0

Table S3b eighted_unifrac_Beta_diversity

	RSM.1	RSM.2	RSM.3	NSM.1	NSM.2	NSM.3	CMSM.1	CMSM.2	CMSM.3
RSM.1	0	0.080547	0.115246	0.314052	0.342793	0.352133	0.601852	0.587717	0.580195
RSM.2	0.080547	0	0.066211	0.298737	0.371242	0.345608	0.618601	0.602909	0.595324
RSM.3	0.115246	0.066211	0	0.30456	0.40272	0.340769	0.6376	0.622402	0.61514
NSM.1	0.314052	0.298737	0.30456	0	0.300168	0.140051	0.615331	0.596394	0.591735
NSM.2	0.342793	0.371242	0.40272	0.300168	0	0.393612	0.481844	0.463359	0.456958
NSM.3	0.352133	0.345608	0.340769	0.140051	0.393612	0	0.677944	0.659787	0.659011
CMSM.1	0.601852	0.618601	0.6376	0.615331	0.481844	0.677944	0	0.051049	0.057274
CMSM.2	0.587717	0.602909	0.622402	0.596394	0.463359	0.659787	0.051049	0	0.03333
CMSM.3	0.580195	0.595324	0.61514	0.591735	0.456958	0.659011	0.057274	0.03333	0

Table S3c unweighted_unifrac

	RSM.1	RSM.2	RSM.3	NSM.1	NSM.2	NSM.3	CMSM.1	CMSM.2	CMSM.3
RSM.1	0	0.219592	0.222028	0.361017	0.350972	0.36266	0.697348	0.651782	0.650443
RSM.2	0.219592	0	0.20277	0.311927	0.337101	0.323823	0.699341	0.638246	0.641079

RSM.3	0.222028	0.20277	0	0.357514	0.358416	0.350342	0.706488	0.653162	0.653962
NSM.1	0.361017	0.311927	0.357514	0	0.312442	0.245427	0.685705	0.644827	0.631275
NSM.2	0.350972	0.337101	0.358416	0.312442	0	0.327079	0.635498	0.585024	0.585052
NSM.3	0.36266	0.323823	0.350342	0.245427	0.327079	0	0.689934	0.646197	0.643487
CMSM.1	0.697348	0.699341	0.706488	0.685705	0.635498	0.689934	0	0.290299	0.305048
CMSM.2	0.651782	0.638246	0.653162	0.644827	0.585024	0.646197	0.290299	0	0.318386
CMSM.3	0.650443	0.641079	0.653962	0.631275	0.585052	0.643487	0.305048	0.318386	0
