# Supplementary material for: Chrysomya megacephala larvae feeding favourably influences manure microbiome, heavy metal stability and greenhouse gas emissions
Source: Microb Biotechnol. 2018 Mar 14;11(3):498–509. doi: 10.1111/1751-7915.13253 (PMC5902325; doi:10.1111/1751-7915.13253)
Supplement: Supplementary file 6 — Table S4. Correlation matrix graph of heavy metal speciation and Prevotella copri (Pcop). [file MBT2-11-498-s006.docx]

**Table S4** Correlation matrix graph of heavy metal speciation and Prevotella copri (Pcop)
