# Supplementary material for: Chrysomya megacephala larvae feeding favourably influences manure microbiome, heavy metal stability and greenhouse gas emissions
Source: Microb Biotechnol. 2018 Mar 14;11(3):498–509. doi: 10.1111/1751-7915.13253 (PMC5902325; doi:10.1111/1751-7915.13253)
Supplement: Supplementary file 8 — Table S6. Different abundance of bacteria species in RSM, NSM and CMSM. [file MBT2-11-498-s008.docx]

Table S6

| Species.NSM-CMSM.differentially abundant | |  |  |  |  |  |  |  |
| --- | --- | --- | --- | --- | --- | --- | --- | --- |
| Species | mean(CMSM) | variance(CMSM) | std.err(CMSM) | mean(NSM) | variance(NSM) | std.err(NSM) | p-vlaue | FDR |
| Acholeplasma_laidlawii | 0.14694 | 1.50E-05 | 0.022095 | 0 | 0 | 0 | 0.014063 | 0.130083 |
| Akkermansia_muciniphila | 0.009373 | 1.00E-06 | 0.004859 | 0.010676 | 2.00E-06 | 0.008738 | 0.896312 | 0.94753 |
| Alcaligenes_faecalis | 0.960375 | 7.90E-05 | 0.051172 | 0.055222 | 1.30E-05 | 0.020985 | 0.003625 | 0.067062 |
| Arcobacter_cryaerophilus | 0.22103 | 9.00E-05 | 0.054836 | 0.010665 | 3.00E-06 | 0.010665 | 0.039438 | 0.132655 |
| Bacteroides_coprosuis | 0.19853 | 1.70E-05 | 0.024011 | 6.979102 | 0.155533 | 2.276934 | 0.06425 | 0.147728 |
| Bacteroides_ovatus | 0.00263 | 0 | 0.00263 | 0.028247 | 2.00E-06 | 0.008247 | 0.067875 | 0.147728 |
| Bacteroides_uniformis | 0 | 0 | 0 | 0.004103 | 1.00E-06 | 0.004103 | 0.347187 | 0.458783 |
| Bdellovibrio_bacteriovorus | 0.00263 | 0 | 0.00263 | 0 | 0 | 0 | 0.500003 | 0.578128 |
| Blautia_obeum | 0.002659 | 0 | 0.00133 | 0.001333 | 0 | 0.001333 | 1 | 1 |
| Blautia_producta | 0.017492 | 1.00E-06 | 0.004956 | 0.017547 | 0 | 0.001486 | 0.959375 | 0.986024 |
| Brevundimonas_diminuta | 0.230466 | 1.00E-04 | 0.057747 | 0.022706 | 7.00E-06 | 0.015366 | 0.046687 | 0.132878 |
| Bulleidia_p-1630-c5 | 0 | 0 | 0 | 0.093404 | 2.90E-05 | 0.030984 | 0.060625 | 0.147728 |
| Clostridium_butyricum | 0.001315 | 0 | 0.001315 | 0.067407 | 2.80E-05 | 0.030333 | 0.092063 | 0.154833 |
| Clostridium_methylpentosum | 0 | 0 | 0 | 0.002666 | 0 | 0.002666 | 0.248198 | 0.340123 |
| Clostridium_tetani | 0 | 0 | 0 | 0.127413 | 6.50E-05 | 0.046573 | 0.075 | 0.151304 |
| Coprococcus_catus | 0 | 0 | 0 | 0.00802 | 1.00E-06 | 0.004618 | 0.117375 | 0.180953 |
| Coprococcus_eutactus | 0 | 0 | 0 | 0.006722 | 0 | 0.001309 | 0.024938 | 0.131815 |
| Desulfovibrio_D168 | 0.001344 | 0 | 0.001344 | 0.070127 | 2.10E-05 | 0.026447 | 0.078625 | 0.151304 |
| Escherichia_coli | 0.039093 | 9.00E-06 | 0.017465 | 0.067918 | 3.50E-05 | 0.034255 | 0.477375 | 0.578128 |
| Eubacterium_biforme | 0 | 0 | 0 | 0.029702 | 1.00E-06 | 0.004954 | 0.021313 | 0.13143 |
| Faecalibacterium_prausnitzii | 0.002672 | 0 | 0.001336 | 0.136451 | 1.40E-05 | 0.021303 | 0.017687 | 0.130884 |
| Fibrobacter_succinogenes | 1.502404 | 0.005412 | 0.42475 | 0.002666 | 0 | 0.002666 | 0.043062 | 0.132775 |
| Flavobacterium_gelidilacus | 3.683779 | 3.30E-05 | 0.033052 | 0.122926 | 0.000316 | 0.10263 | 0 | 0 |
| Geovibrio_thiophilus | 0.002688 | 0 | 0.002688 | 0 | 0 | 0 | 0.500003 | 0.578128 |
| Lactobacillus_mucosae | 0.003945 | 0 | 0.003945 | 0.005423 | 0 | 0.002712 | 0.74625 | 0.836705 |
| Lactobacillus_reuteri | 0.10167 | 0 | 0.00258 | 1.095319 | 0.002705 | 0.300251 | 0.0535 | 0.141393 |
| Myroides_odoratimimus | 0.100122 | 0.000233 | 0.088129 | 0.127569 | 0.000162 | 0.073574 | 0.815875 | 0.887864 |
| Prevotella_copri | 0.005402 | 0 | 0.002701 | 3.128826 | 0.015657 | 0.722416 | 0.032188 | 0.132328 |
| Prevotella_stercorea | 0 | 0 | 0 | 0.227122 | 7.50E-05 | 0.050069 | 0.028563 | 0.132104 |
| Pseudoclavibacter_bifida | 0.002688 | 0 | 0.002688 | 0 | 0 | 0 | 0.500003 | 0.578128 |
| Ruminococcus_bromii | 0 | 0 | 0 | 0.512022 | 0.000525 | 0.132241 | 0.035812 | 0.132504 |
| Ruminococcus_torques | 0.00536 | 0 | 0.001353 | 0.001333 | 0 | 0.001333 | 0.098875 | 0.15906 |
| Ruminofilibacter_xylanolyticum | 0.031022 | 5.00E-06 | 0.013014 | 0 | 0 | 0 | 0.08225 | 0.151304 |
| Salinispora_tropica | 0 | 0 | 0 | 0.002736 | 0 | 0.002736 | 0.248198 | 0.340123 |
| Selenomonas_lacticifex | 0 | 0 | 0 | 0.088456 | 9.20E-05 | 0.055262 | 0.17025 | 0.25197 |
| Streptococcus_luteciae | 0.04949 | 1.00E-06 | 0.004632 | 3.159619 | 0.055013 | 1.354166 | 0.085875 | 0.151304 |
| Unclassified | 92.67488 | 0.004505 | 0.387513 | 83.786551 | 0.015158 | 0.710826 | 0.00725 | 0.089417 |

Table S4b

| Species.RSM-CMSM.differentially abundant | |  |  |  |  |  |  |  |
| --- | --- | --- | --- | --- | --- | --- | --- | --- |
| Species | mean(CMSM) | variance(CMSM) | std.err(CMSM) | mean(RSM) | variance(RSM) | std.err(RSM) | p-vlaue | FDR |
| Acholeplasma_laidlawii | 0.14694 | 1.50E-05 | 0.022095 | 0 | 0 | 0 | 0.029032 | 0.101612 |
| Akkermansia_muciniphila | 0.009373 | 1.00E-06 | 0.004859 | 0.008623 | 2.00E-06 | 0.008623 | 0.925645 | 0.925645 |
| Alcaligenes_faecalis | 0.960375 | 7.90E-05 | 0.051172 | 0.031414 | 4.00E-06 | 0.012138 | 0.003226 | 0.056455 |
| Arcobacter_cryaerophilus | 0.22103 | 9.00E-05 | 0.054836 | 0.044337 | 2.00E-06 | 0.007593 | 0.067194 | 0.121887 |
| Bacteroides_coprosuis | 0.19853 | 1.70E-05 | 0.024011 | 0.69481 | 0.001056 | 0.187609 | 0.070419 | 0.121887 |
| Bacteroides_ovatus | 0.00263 | 0 | 0.00263 | 0.004312 | 1.00E-06 | 0.004312 | 0.797839 | 0.821305 |
| Bacteroides_uniformis | 0 | 0 | 0 | 0.001437 | 0 | 0.001437 | 0.483513 | 0.55959 |
| Bdellovibrio_bacteriovorus | 0.00263 | 0 | 0.00263 | 0 | 0 | 0 | 0.50054 | 0.55959 |
| Blautia_obeum | 0.002659 | 0 | 0.00133 | 0.007141 | 0 | 0.001415 | 0.080097 | 0.121887 |
| Blautia_producta | 0.017492 | 1.00E-06 | 0.004956 | 0.01 | 0 | 0.003762 | 0.241548 | 0.325161 |
| Brevundimonas_diminuta | 0.230466 | 1.00E-04 | 0.057747 | 0 | 0 | 0 | 0.057742 | 0.118549 |
| Bulleidia_p-1630-c5 | 0 | 0 | 0 | 0.065782 | 6.00E-06 | 0.013661 | 0.038452 | 0.112152 |
| Butyricicoccus_pullicaecorum | 0 | 0 | 0 | 0.035741 | 2.00E-06 | 0.007547 | 0.041677 | 0.112207 |
| Clostridium_butyricum | 0.001315 | 0 | 0.001315 | 0.051462 | 1.00E-06 | 0.004245 | 0.009677 | 0.084674 |
| Coprococcus_catus | 0 | 0 | 0 | 0.015726 | 0 | 0.003773 | 0.054516 | 0.118549 |
| Coprococcus_eutactus | 0 | 0 | 0 | 0.031455 | 1.00E-06 | 0.005151 | 0.032258 | 0.102639 |
| Desulfovibrio_D168 | 0.001344 | 0 | 0.001344 | 0.060121 | 6.00E-06 | 0.013969 | 0.05129 | 0.118549 |
| Escherichia_coli | 0.039093 | 9.00E-06 | 0.017465 | 0.081574 | 1.10E-05 | 0.018916 | 0.127871 | 0.179019 |
| Eubacterium_biforme | 0 | 0 | 0 | 0.018622 | 2.00E-06 | 0.007608 | 0.073645 | 0.121887 |
| Faecalibacterium_prausnitzii | 0.002672 | 0 | 0.001336 | 0.237444 | 1.70E-05 | 0.024049 | 0.012903 | 0.090321 |
| Fibrobacter_succinogenes | 1.502404 | 0.005412 | 0.42475 | 0 | 0 | 0 | 0.060968 | 0.118549 |
| Flavobacterium_gelidilacus | 3.683779 | 3.30E-05 | 0.033052 | 0.007148 | 0 | 0.003774 | 0 | 0 |
| Geovibrio_thiophilus | 0.002688 | 0 | 0.002688 | 0 | 0 | 0 | 0.50054 | 0.55959 |
| Lactobacillus_mucosae | 0.003945 | 0 | 0.003945 | 0.001425 | 0 | 0.001425 | 0.527613 | 0.55959 |
| Lactobacillus_reuteri | 0.10167 | 0 | 0.00258 | 0.732328 | 0.000224 | 0.086415 | 0.025806 | 0.100357 |
| Myroides_odoratimimus | 0.100122 | 0.000233 | 0.088129 | 0.044297 | 6.00E-06 | 0.014249 | 0.524387 | 0.55959 |
| Prevotella_copri | 0.005402 | 0 | 0.002701 | 10.465323 | 0.012684 | 0.650233 | 0.006452 | 0.075273 |
| Prevotella_stercorea | 0 | 0 | 0 | 0.992823 | 0.000443 | 0.121579 | 0.022581 | 0.098792 |
| Pseudoclavibacter_bifida | 0.002688 | 0 | 0.002688 | 0 | 0 | 0 | 0.50054 | 0.55959 |
| Ruminococcus_bromii | 0 | 0 | 0 | 0.11731 | 2.20E-05 | 0.027339 | 0.048065 | 0.118549 |
| Ruminococcus_torques | 0.00536 | 0 | 0.001353 | 0.001425 | 0 | 0.001425 | 0.095968 | 0.139953 |
| Ruminofilibacter_xylanolyticum | 0.031022 | 5.00E-06 | 0.013014 | 0 | 0 | 0 | 0.076871 | 0.121887 |
| Selenomonas_lacticifex | 0 | 0 | 0 | 0.001437 | 0 | 0.001437 | 0.483513 | 0.55959 |
| Streptococcus_luteciae | 0.04949 | 1.00E-06 | 0.004632 | 0.983832 | 0.000384 | 0.113138 | 0.019355 | 0.096775 |
| Unclassified | 92.67488 | 0.004505 | 0.387513 | 85.252653 | 0.013108 | 0.661016 | 0.016129 | 0.094086 |

Table S4c

| Species.RSM-NSM.differentially abundant | | | |  |  |  |  |  |
| --- | --- | --- | --- | --- | --- | --- | --- | --- |
| Species | mean(NSM) | variance(NSM) | std.err(NSM) | mean(RSM) | variance(RSM) | std.err(RSM) | p-vlaue | FDR |
| Akkermansia_muciniphila | 0.010676 | 2.00E-06 | 0.008738 | 0.008623 | 2.00E-06 | 0.008623 | 0.8291 | 0.855009 |
| Alcaligenes_faecalis | 0.055222 | 1.30E-05 | 0.020985 | 0.031414 | 4.00E-06 | 0.012138 | 0.375367 | 0.53857 |
| Arcobacter_cryaerophilus | 0.010665 | 3.00E-06 | 0.010665 | 0.044337 | 2.00E-06 | 0.007593 | 0.072 | 0.216 |
| Bacteroides_coprosuis | 6.979102 | 0.155533 | 2.276934 | 0.69481 | 0.001056 | 0.187609 | 0.050933 | 0.200933 |
| Bacteroides_ovatus | 0.028247 | 2.00E-06 | 0.008247 | 0.004312 | 1.00E-06 | 0.004312 | 0.068133 | 0.216 |
| Bacteroides_uniformis | 0.004103 | 1.00E-06 | 0.004103 | 0.001437 | 0 | 0.001437 | 0.553467 | 0.6523 |
| Blautia_obeum | 0.001333 | 0 | 0.001333 | 0.007141 | 0 | 0.001415 | 0.035933 | 0.197631 |
| Blautia_producta | 0.017547 | 0 | 0.001486 | 0.01 | 0 | 0.003762 | 0.112667 | 0.309834 |
| Brevundimonas_diminuta | 0.022706 | 7.00E-06 | 0.015366 | 0 | 0 | 0 | 0.203 | 0.418688 |
| Bulleidia_p-1630-c5 | 0.093404 | 2.90E-05 | 0.030984 | 0.065782 | 6.00E-06 | 0.013661 | 0.4725 | 0.611634 |
| Butyricicoccus_pullicaecorum | 0 | 0 | 0 | 0.035741 | 2.00E-06 | 0.007547 | 0.0148 | 0.154003 |
| Clostridium_butyricum | 0.067407 | 2.80E-05 | 0.030333 | 0.051462 | 1.00E-06 | 0.004245 | 0.6299 | 0.716783 |
| Clostridium_methylpentosum | 0.002666 | 0 | 0.002666 | 0 | 0 | 0 | 0.500428 | 0.611634 |
| Clostridium_tetani | 0.127413 | 6.50E-05 | 0.046573 | 0 | 0 | 0 | 0.0548 | 0.200933 |
| Coprococcus_catus | 0.00802 | 1.00E-06 | 0.004618 | 0.015726 | 0 | 0.003773 | 0.277767 | 0.49295 |
| Coprococcus_eutactus | 0.006722 | 0 | 0.001309 | 0.031455 | 1.00E-06 | 0.005151 | 0.018667 | 0.154003 |
| Desulfovibrio_D168 | 0.070127 | 2.10E-05 | 0.026447 | 0.060121 | 6.00E-06 | 0.013969 | 0.744733 | 0.79278 |
| Escherichia_coli | 0.067918 | 3.50E-05 | 0.034255 | 0.081574 | 1.10E-05 | 0.018916 | 0.734567 | 0.79278 |
| Eubacterium_biforme | 0.029702 | 1.00E-06 | 0.004954 | 0.018622 | 2.00E-06 | 0.007608 | 0.291567 | 0.49295 |
| Faecalibacterium_prausnitzii | 0.136451 | 1.40E-05 | 0.021303 | 0.237444 | 1.70E-05 | 0.024049 | 0.0288 | 0.19008 |
| Fibrobacter_succinogenes | 0.002666 | 0 | 0.002666 | 0 | 0 | 0 | 0.500428 | 0.611634 |
| Flavobacterium_gelidilacus | 0.122926 | 0.000316 | 0.10263 | 0.007148 | 0 | 0.003774 | 0.3217 | 0.49295 |
| Lactobacillus_mucosae | 0.005423 | 0 | 0.002712 | 0.001425 | 0 | 0.001425 | 0.257433 | 0.49295 |
| Lactobacillus_reuteri | 1.095319 | 0.002705 | 0.300251 | 0.732328 | 0.000224 | 0.086415 | 0.3144 | 0.49295 |
| Myroides_odoratimimus | 0.127569 | 0.000162 | 0.073574 | 0.044297 | 6.00E-06 | 0.014249 | 0.328633 | 0.49295 |
| Prevotella_copri | 3.128826 | 0.015657 | 0.722416 | 10.46532 | 0.012684 | 0.650233 | 0 | 0 |
| Prevotella_stercorea | 0.227122 | 7.50E-05 | 0.050069 | 0.992823 | 0.000443 | 0.121579 | 0.003867 | 0.063806 |
| Ruminococcus_bromii | 0.512022 | 0.000525 | 0.132241 | 0.11731 | 2.20E-05 | 0.027339 | 0.043433 | 0.200933 |
| Ruminococcus_torques | 0.001333 | 0 | 0.001333 | 0.001425 | 0 | 0.001425 | 1 | 1 |
| Salinispora_tropica | 0.002736 | 0 | 0.002736 | 0 | 0 | 0 | 0.500428 | 0.611634 |
| Selenomonas_lacticifex | 0.088456 | 9.20E-05 | 0.055262 | 0.001437 | 0 | 0.001437 | 0.162367 | 0.382722 |
| Streptococcus_luteciae | 3.159619 | 0.055013 | 1.354166 | 0.983832 | 0.000384 | 0.113138 | 0.1585 | 0.382722 |
| Unclassified | 83.78655 | 0.015158 | 0.710826 | 85.25265 | 0.013108 | 0.661016 | 0.199133 | 0.418688 |
