# Supplementary material for: Chrysomya megacephala larvae feeding favourably influences manure microbiome, heavy metal stability and greenhouse gas emissions
Source: Microb Biotechnol. 2018 Mar 14;11(3):498–509. doi: 10.1111/1751-7915.13253 (PMC5902325; doi:10.1111/1751-7915.13253)
Supplement: Supplementary file 9 — Table S7. Primers used for quantitative PCR. [file MBT2-11-498-s009.docx]

**Table S7 Primers used for quantitative PCR.**

| **Target** | **Primer sequence (5´→3´)** | **References** |
| --- | --- | --- |
| Methanogens-Forward | TTCGGTGGATCDCARAGRGC | Duarte et al. 2017 |
| Methanogens-Reverse | GBARGTCGWAWCCGTAGAATCC |  |
| Methanomassiliicoccaceae-Forward | GAAGCCCTRGGTCGCAAA | Duarte et al. 2017 |
| Methanomassiliicoccaceae-Reverse | TACTCCCCAAGTRGCMGACTT |  |
| Methanobrevibacter-Forward | CCTCCGCAATGTGAGAAATCGC | Duarte et al. 2017 |
| Methanobrevibacter-Reverse | TCWCCAGCAATTCCCACAGTT |  |
| *Salmonella* sp.-Forward | CGTTTCCTGCGGTACTGTTAATT | Lee et al. 2006 |
| *Salmonella* sp.-Reverse | AGACGGCTGGTACTGATCGATAA |  |
| 16S-Forward | CGGTGAATACGTTCYCGG | Gaze et al. 2011 |
| 16S-Reverse | GGWTACCTTGTTACGACTT |  |

**References**

Duarte, A.C., Durmic, Z., Vercoe, P.E. and Chaves, A.V., 2017. Dose-response effects of dietary pequi oil on fermentation characteristics and microbial population using a rumen simulation technique (Rusitec). Anaerobe. 48, 59-65.

Gaze, W.H., Zhang, L., Abdouslam, N.A., Hawkey, P.M., Calvo-Bado, L., Royle, J., Brown, H., Davis, S., Kay, P. and Boxall, A.B., 2011. Impacts of anthropogenic activity on the ecology of class 1 integrons and integron-associated genes in the environment. The ISME Journal 5, 1253-1261.

Lee, D.Y., Shannon, K. and Beaudette, L.A., 2006. Detection of bacterial pathogens in municipal wastewater using an oligonucleotide microarray and real-time quantitative PCR. Journal of Microbiological Methods 65, 453-467.
